# Machine learning designs new GCGR/GLP-1R dual agonists with enhanced biological potency

**DOI:** 10.1038/s41557-024-01532-x

**Published:** 2024-05-16

**Authors:** Anna M. Puszkarska, Bruck Taddese, Jefferson Revell, Graeme Davies, Joss Field, David C. Hornigold, Andrew Buchanan, Tristan J. Vaughan, Lucy J. Colwell

**Affiliations:** 1 Yusuf Hamied Department of Chemistry, University of Cambridge, Cambridge, UK; 2 Discovery Sciences, R&D, AstraZeneca, Cambridge, UK; 3 Research and Early Development, Cardiovascular, Renal and Metabolism (CVRM), BioPharmaceuticals R&D, AstraZeneca, Cambridge, UK; 4 Biologics Engineering, Oncology R&D, AstraZeneca, Cambridge, UK; 5 Google DeepMind, Cambridge, MA USA; 6 Present Address: Biologics Engineering, Oncology R&D, AstraZeneca, Cambridge, UK; 7 Present Address: Biologics Center (NBC) at the Novartis Institute for BioMedical Research (NIBR), Basel, Switzerland; 8 Present Address: Immunocore Ltd., Abingdon, UK

**Keywords:** Machine learning, Recombinant peptide therapy, Computational chemistry

## Abstract

Several peptide dual agonists of the human glucagon receptor (GCGR) and the glucagon-like peptide-1 receptor (GLP-1R) are in development for the treatment of type 2 diabetes, obesity and their associated complications. Candidates must have high potency at both receptors, but it is unclear whether the limited experimental data available can be used to train models that accurately predict the activity at both receptors of new peptide variants. Here we use peptide sequence data labelled with in vitro potency at human GCGR and GLP-1R to train several models, including a deep multi-task neural-network model using multiple loss optimization. Model-guided sequence optimization was used to design three groups of peptide variants, with distinct ranges of predicted dual activity. We found that three of the model-designed sequences are potent dual agonists with superior biological activity. With our designs we were able to achieve up to sevenfold potency improvement at both receptors simultaneously compared to the best dual-agonist in the training set.

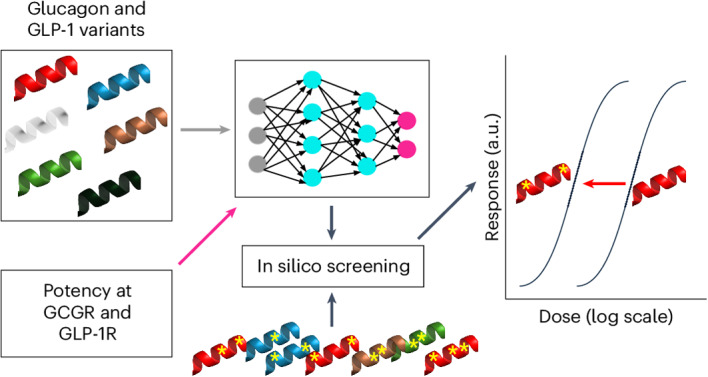

## Abstract (web summary)

Engineering new ligands that specifically target multiple G protein-coupled receptors with desired activity profiles requires time-consuming and expensive cycles of design-make-test-analyse work. Now it has been shown that limited experimental data can be used to train sophisticated machine learning models to accurately predict the activity of previously uncharacterized peptide ligand variants.

## Main

Peptide hormones signal through cell membrane receptors to communicate and regulate a myriad of physiological processes, including energy metabolism, growth, sleep and blood pressure. Helical peptides that are known to play key roles in maintaining metabolic homeostasis include, among others, glucagon (GCG) and glucagon-like peptide-1 (GLP-1), which signal and agonise the G-protein-coupled receptors (GPCR) GCGR and GLP-1R, respectively^1^. GLP-1R agonists have been shown to lower blood glucose, inhibit food intake and substantially reduce body weight^2^. Chemical analogues of GLP-1 are currently approved for the treatment of type 2 diabetes (T2D) and obesity^3,4^. Moreover, peptide analogues that are unimolecular co-agonists of both GLP-1R and GCGR are currently in clinical development for the treatment of T2D, obesity and non-alcoholic steatohepatitis (NASH)^2,3,5–7^.

Several studies have been carried out with the goal of deriving new class B GPCR targeting agents^8–13^. The design of high-potency unimolecular GCGR/GLP-1R dual agonists has revolved around substitutions in the mid and C-terminal segments of proposed analogues^5,14–16^. Determination of the receptor–ligand co-crystal structures and mutational studies have led to a two-step model of peptide binding and receptor activation^17–20^. The mechanism is thought to involve binding of the peptide C terminus to the receptor extracellular domain, followed by insertion of the peptide N terminus into the pocket formed by the transmembrane helices and extracellular loops, instigating receptor activation and signalling^19^.

However, despite recent progress in understanding the mechanisms of receptor activation, the relationship between peptide sequence and functional activity is not fully understood. Engineering new peptides with desired selectivity profiles requires time-consuming and expensive cycles of design-make-test-analyse (DMTA) work. To address this problem, we propose that existing experimental data can be used to train machine learning (ML) models that are able to extrapolate in sequence space to accurately predict the activity of novel multi-specific peptide analogues. Recently, several efforts have been focused on the development of ML models for peptide^21,22^ and small-molecule^23,24^ design, demonstrating the success of these approaches in de novo prediction of functional molecules.

In this Article we use a set of 125 experimentally characterized glucagon and GLP-1 peptide analogues to train models that capture the relationship between peptide sequence and agonism or receptor activation at both the GCGR and GLP-1R. The degree of receptor activation is typically reported as the peptide concentration required for a response to reach 50% of its maximal value, known as the half-maximal effective concentration (EC_50_). A lower EC_50_ indicates a higher peptide potency, or ability to agonise receptor signalling. In this Article, the EC_50_ values at human GCGR and GLP-1R for cyclic adenosine monophosphate (cAMP) second messenger signalling were set as the optimization targets. The experimental EC_50_ values were generated using in vitro cell-based activity assays.

Our models assume that the biological activity of a peptide is dictated by its primary sequence, which we represent using simple one-hot encoding^25–27^. We compared several different regression model architectures, evaluating model performance using held-out test sequences that were distinct from those used to train the model. Surprisingly, given the limited amount of training data, we found that an ensemble of multi-task convolutional neural-network (CNN) models that simultaneously predict potency at both GCGR and GLP-1R provides significantly better performance against GLP-1R, whereas performance differences against GCGR were largely not significant. To prospectively test the ability of this model to design new peptides with specific activity, we used a simple optimization strategy to design 15 peptides, five in each of three different activity profiles: selective potency at GCGR, selective potency at GLP-1R or high-potency at both receptors.

## Results

### Training data

**Fig. 1:**
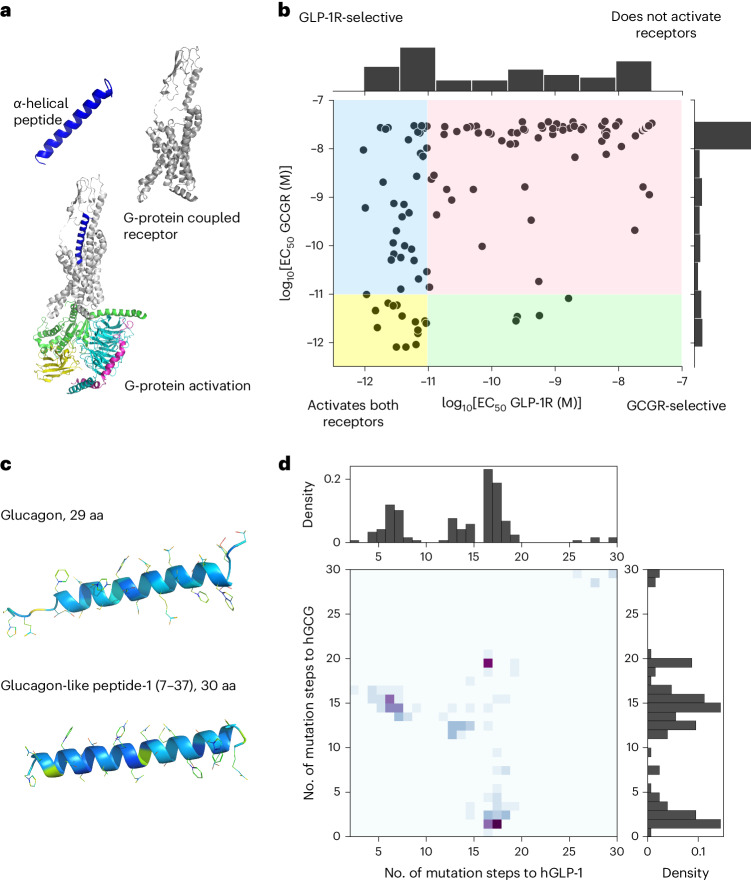
Building models that predict peptide potency at both GCGR and GLP-1R. **a**, The α-helical peptide (top left) binds to the extracellular domain of the GPC receptor (top right), inducing a change in receptor conformation that in turn binds to the G-protein at the cytoplasmic interface (bottom) (PDB 5VAI). **b**, Sequence variants (125 examples) are divided into four categories based on their activities: (1) strongly activates both receptors (yellow area), selectively activates (2) GCGR (green) or (3) GLP-1R (blue), or (4) activates neither receptor (pink). The activity threshold of −11 (that is, 10 pM) is chosen as being within tenfold of the native ligand activity for each receptor. **c**, Structures of human glucagon and glucagon-like peptide-1 (7-37) (PDB 1GCN and 6X18). **d**, Histogram showing the joint distribution of the number of mutations to the natural ligands for the training dataset, as well as the marginal mutation distribution for each reference molecule. aa, amino acids. Source data

We used experimental potency measurements (EC_50_) at both GCGR and GLP-1R for a total of 125 peptide variants to fit models that relate peptide sequences to in vitro activity against each receptor. Figure 1b visualizes these data together with four activity regions representing the possible potency measurements at hGCG and hGLP-1 for dual-agonist peptides. We note that variants with high potency at GCGR are under-represented in this dataset (14.4%) compared with peptides that are strongly potent at GLP-1R (37.6%). In particular, only four peptide variants selectively activated GCGR (3.2%, region marked in green). In contrast, several peptides with high potency at GLP-1R are in fact GLP-1R-selective (25.6% of all training-set examples). Dual agonists comprise 11.2% of the training set, and the best representative has potencies of log_10_(EC_50_) = −12.08 (0.83 pM) and −11.50 (3.19 pM) at hGCGR and hGLP-1R, respectively. Notably, nearly 60% of training-set examples are inactive at both receptors.

The glucagon peptide contains 29 amino acids, and bioactive GLP-1 contains 30 amino acids terminating in an amide or 31 amino acids terminating in an acid. Overall, the native hGCG and hGLP-1 peptide sequences differ at 15 of 29 positions. The addition of a C-terminal amide to glucagon together with modification of position 14 has previously been shown to stabilize the C-terminal helix and enhance GLP-1R binding while maintaining activity against GCGR^14,20^.

Our training dataset contains examples with as few as two and as many as 20 modifications from the wild-type human glucagon sequence, with some examples containing mutations drawn from the wild-type human GLP-1 sequence. In Fig. 1d, a histogram shows the distribution of the number of mutations across the training data. The average distance of a training set sequence to hGCG is 13.4 with a standard deviation of 5.7, and on average there are 11 point mutations to hGLP-1 with a standard deviation of 7.24. Moreover, at least 26 of 125 training samples are variants of GLP-1 in which between one and five amino acids have been deleted, resulting in no significant activity at either GCGR or GLP-1R. A further four variants have deletions in the last part of the C-terminal region; however, these analogues do retain potency at GLP-1R. In contrast, a variant with five mutations from glucagon, none of which are present in GLP-1, activates both receptors, while many variants with some GLP-1 chimera mutations and some other mutations report various potencies at both receptors. It has previously been observed that the GLP-1R does not readily distinguish the N-terminal regions of these two hormones^16^, although our data contain examples where a single mutation in the N-terminal region of GLP-1 abrogates GLP-1R activity, suggesting additional nuances in the landscape relating peptide sequence to functional activity.

**Fig. 2:**
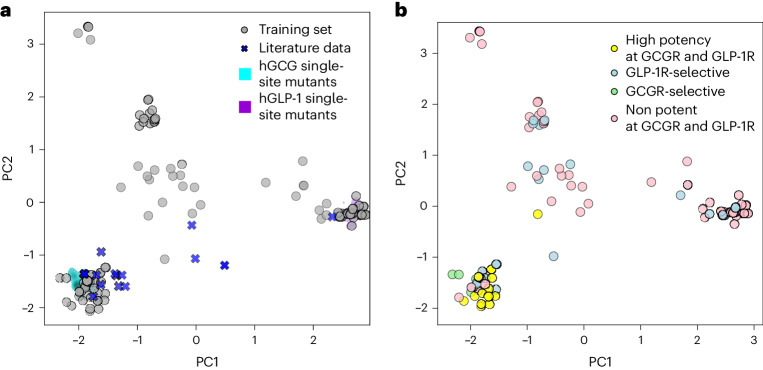
Low-dimensional representation of the training data. **a**, Projection of the 125 training set sequences (grey) and 19 independent sequences from the literature (blue)^15^ onto the first (PC1, *x* axis) and second (PC2, *y* axis) principal components of the covariance matrix of the training set. All possible single-site mutants of human glucagon (cyan) and human GLP-1 (purple) are included as a reference to facilitate evaluation of sequence diversity across the training set samples. **b**, Projected training set sequences coloured by their potency category, following the colour scheme introduced in Fig. 1b. Source data

To describe the distribution of the training data in terms of sequence similarity, we used principal component analysis (PCA). Figure 2 shows the training data projected into the two-dimensional (2D) space determined by the first and second principal components. Here, the covariance matrix *C*^*L*21 × *L*21^ has been determined for the array of one-hot-encoded training-set sequences. We also projected the complete sets of possible hGCG and hGLP-1 single point mutants into this space, to provide a sense of scale. This data projection reveals that the training set contains subsets of sequences that are a few mutations apart from hGCG (Fig. 2a, bottom left) and from hGLP-1 (Fig. 2a, bottom right). In addition, a number of sequence variants are roughly equidistant to both wild-type peptides (Fig. 2a, central region). Figure 2b shows that close hGLP-1 analogues tend to be inactive at both receptors, or to exclusively activate hGLP-1R. Similarly, sequences that are hGCGR-selective exhibit close similarity to human glucagon. Surprisingly, these data suggest that high peptide potency against both receptors might require close sequence homology to glucagon, suggesting that the hGCG receptor might tolerate ligand mutations to a lower extent than the hGLP-1 receptor.

### Model training and evaluation

We fit a set of supervised regression models using sixfold cross-validation by dividing the data into 105 training sequences, 10 validation sequences and 10 held-out test sequences for each fold to tune the model hyperparameters (Methods and Table 1). We then compared the performance of these models to a neural-network model that included both convolutional and fully connected layers, as described in the Methods and with the results summarized in Table 1, Supplementary Table 1 and Supplementary Fig. 1. To identify optimal hyperparameters for our deep model and for the baseline models, we used the same sixfold cross-validation scheme (Methods). After tuning the model hyperparameters, we then retrained the deep models using 120 samples for training, keeping five samples as a validation set with which to monitor performance on unseen data during training. All other models were retrained on the entire dataset.

**Table 1:** Model performance evaluation using sixfold cross-validation to split the AstraZeneca experimental data and model validation using measurements for 19 peptides from Day et al.^15^ that are distinct from any of the sequences contained in the AstraZeneca dataset (sequences shown in Extended Data Table 1)

Models	Cross-validation on training data		Validation on held-out literature data^15^	
	R.m.s.e. GCGR	R.m.s.e. GLP-1R	R.m.s.e. GCGR	R.m.s.e. GLP-1R
Ridge	0.63 ± 0.06	0.75 ± 0.10	1.12 ± NA	0.63 ± NA
SVR	0.60 ± 0.04	0.81 ± 0.07	1.09 ± NA	0.45 ± NA
GPR	0.69 ± 0.17	0.88 ± 0.23	1.25 ± NA	**0.25** ± **NA**
Random forest	0.62 ± 0.04	0.77 ± 0.06	0.98 ± 0.05	0.61 ± 0.06
NN single-task	0.67 ± 0.07	0.78 ± 0.07	0.92 ± 0.18	0.41 ± 0.11
NN multi-task	0.68 ± 0.06	0.78 ± 0.05	1.04 ± 0.08	0.73 ± 0.36
NN multi-task ensemble	**0.59** ± **0.05**	**0.68** ± **0.04**	**0.86** ± **0.02**	0.53 ± 0.08

Other model performance metrics are presented in Supplementary Tables 1 and 5. The root-mean-square error (r.m.s.e.) metric value shown for each model in the cross-validation columns is the average and the standard deviation computed over ten independent instantiations using different data splits. The error bars reported in the model validation columns result from models being retrained three times on the same data with different parameters initialization (model initialization variance). SVR, support vector regression; GPR, Gaussian process regression; NN, neural network; NA, value cannot be estimated. The best result in each category is highlighted in bold.

We then asked whether multi-task learning (equation (1)) could be used to train a model that predicts peptide potency against both receptors simultaneously. Multi-task learning aims to improve generalization and increase prediction accuracy by learning objectives for several target variables from shared representations^28^. The predictions made by neural-network models are subject to stochastic variation due to factors such as the random initialization of model parameters. To increase model robustness and mitigate epistemic uncertainty, we trained multiple copies of the same multi-task neural-network model and built a simple committee model, in which the final prediction is given by the average of the individual model predictions (details are provided in the Methods and Supplementary Fig. 2). The ensemble of 12 multi-task convolutional models achieves significantly better performance against GLP-1R across ten iterations of sixfold cross-validation on the training data (*t*-test, *P* = 0.05; Supplementary Table 3 and Supplementary Fig. 3), whereas performance differences against GCGR were largely not significant. We also built a simple nearest-neighbours model, and trained adversarial control models using data with shuffled sequences or targets (Supplementary Table 4 and Supplementary Fig. 4).

To further validate our models, we identified an additional set of peptide sequences from ref. ^15^ for which activity against GCGR and GLP-1R was biochemically characterized in vitro. Our trained models were used to make predictions for sequence variants from this dataset, without adjusting the model hyperparameters or weights. Table 1b shows that although overall model predictions on these data are less accurate, probably due to differences in the potency assays such as expected receptor expression levels and host cell background, the neural-network ensemble reports reasonable performance across both targets (also Extended Data Table 1, Supplementary Table 5 and Supplementary Fig. 5). Consequently, we decided to further evaluate the predictive accuracy of the multi-task ensemble model by testing its ability to carry out model-guided peptide design.

### Ligand design

**Fig. 3:**
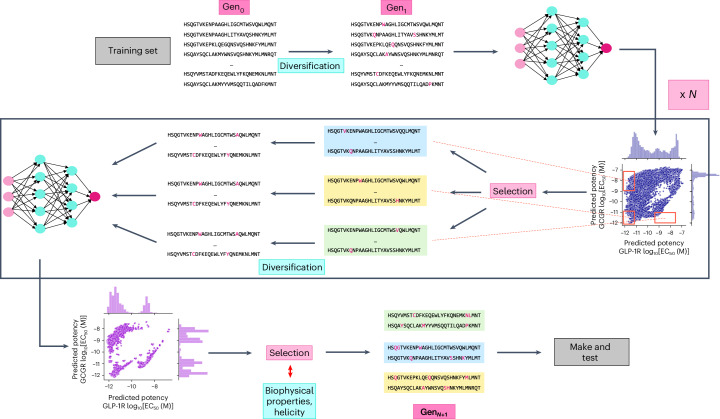
Schematic representation of the model-guided ligand design directed evolution workflow. Training set sequences (*G*_0_) were used as a starting point. Sequences were then evolved by introducing a single point mutation. The potency at human GCGR and GLP-1R was then predicted for each sequence using the trained neural-network multi-task ensemble model. Next, a subset of sequences predicted to obtain the design goal was extracted and used as a starting point for the next generation (*G*_N_). This cycle was repeated three times (*N* = 3). The sequences from the first and last generation that best met the design goals were selected and their computationally predicted biophysical properties compared with the training set. A fraction of these were then chosen before chemical synthesis and tested in vitro.

We next asked whether we can use our trained multi-task ensemble model to design peptide sequences that have (1) high potency for both GCGR and GLP-1R, (2) selective potency for GCGR or (3) selective potency for GLP-1R using the EC_50_ ranges defined in the Methods. To proceed, for each desired potency profile we carried out a directed search of sequence space that involved three rounds of model-guided sequence optimization, starting from training sequences with desirable potency values, and retaining the best variants from each round as the starting point for the next generation. After each round of optimization, we retrieved the 50 sequence variants that the model predicts to have the best potencies, and of these, the five to ten most diverse sequence variants were used as the starting points for the next round of optimization (see Methods for details). Finally, we applied an additional filter to the best candidates to ensure that their predicted chemical and biophysical properties aligned well with those training set samples that had the required potency profile. This process is illustrated in Fig. 3.

For each design category we retained the 50 sequences from each of the first and third generations with best model-predicted potencies. The mutual information (equation (5)) between the model-designed sequences in each category and the training set is visualized in Supplementary Fig. 6 (see Methods for details). For each design category, we estimated the probabilities of occurrence of amino acids at each site (position-specific scoring matrix (PSSM)) and calculated the entropy (equation (6)) across sequence positions in each generation of sequences. This analysis shed light on sequence regions that the model considered functionally important for each activity category, and we observed regions that were highly conserved or highly mutable (Supplementary Fig. 6). It is known that glucagon analogues with high potency tend to have strong helicity, together with an isoelectric point, stability and hydrophobicity that fall within specific ranges^11,12,29^. Similarly, helicity also impacts the activity of GLP-1 analogues^30^. These biophysical properties can be predicted from the sequence using well-known algorithms. We used the training set ranges of six biophysical properties (listed in the Methods) as additional criteria to further filter these 100 model-optimized sequences. To proceed, we divided our training set data into three groups using the potency regions shown in Fig. 1, and calculated the mean and standard deviation of each property within these groups. Then, for each optimized sequence, we computed the number of features for which the estimated property value was within one standard deviation of the mean calculated for the corresponding group of training-set sequences.

**Extended Data Fig. 1:**
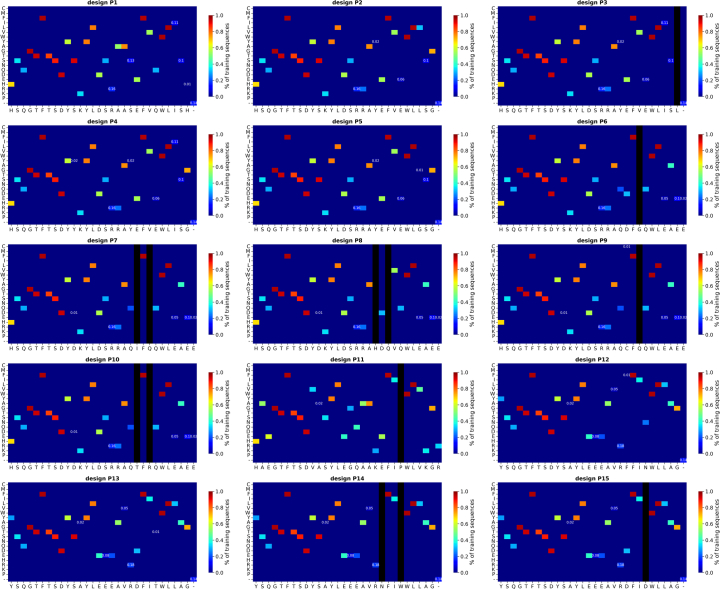
Comparison between 15 model-designed compounds and the training data. Each panel shows a construct (P1 - P15, using the same notation as in the main text) as an array whose entries reflect the prevalence of each amino acid at specific position in the training set, according to the legend on the right side. Rarely occurring mutations - frequency in the training set lower than 0.2 - are indicated by the number. Black vertical stripes mark new sequence substitutions which do not occur in the training set. Source data

We prioritized samples that passed this screen while also considering sequence diversity measured by the point mutation distance among candidates within the same potency category to select 15 sequences, five designs for each of the three potency profiles, for experimental validation. The biophysical properties monitored during the selection process are listed for these final analogues in Extended Data Table 2, and a comparison between each sequence and the training data is provided in Extended Data Fig. 1. We followed the same design process for each baseline model, generating sets of five designs for each potency category for each model (Supplementary Fig. 7 and Supplementary Tables 6–11). We note that the predicted potencies for all sequence designs are highly consistent across the different models. Supplementary Fig. 7 shows a PCA analysis of the sequences designed by different models, which suggests that different models explore different regions of sequence space, as previously reported^31^.

### Prospective experimental validation

**Fig. 4:**
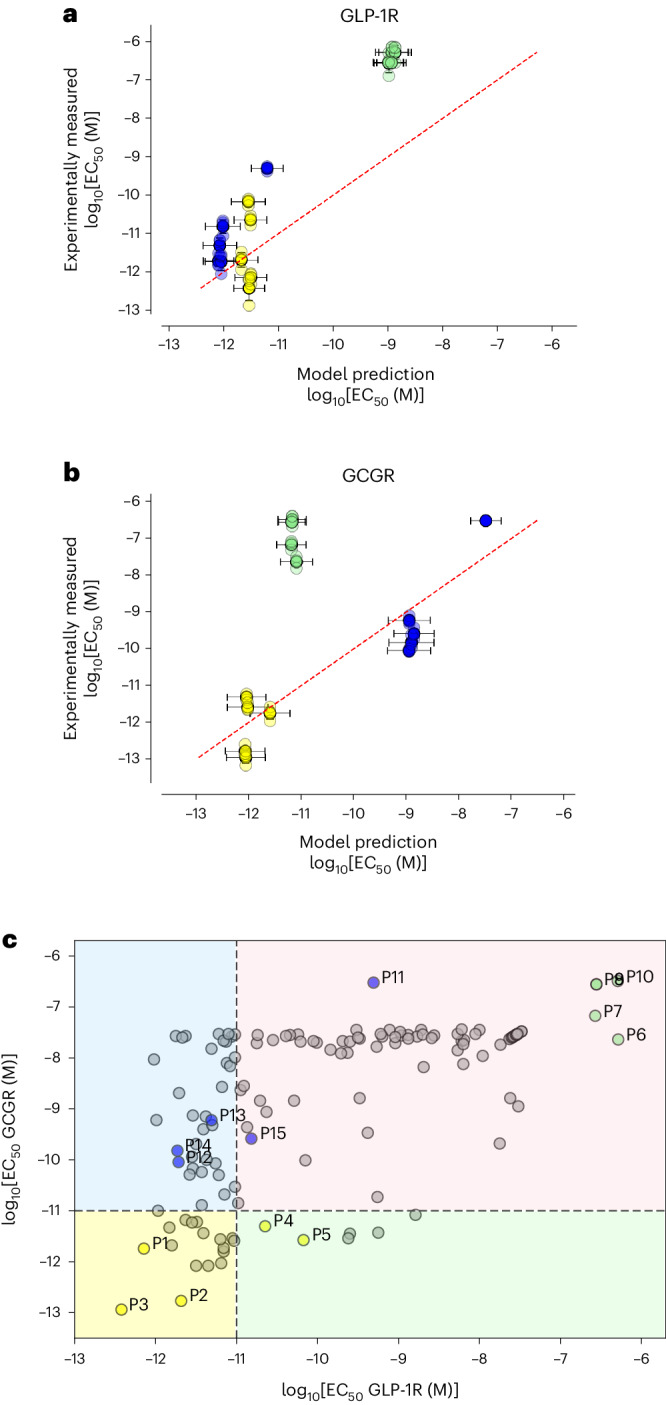
Experimental evaluation of predictions. **a**,**b**, The 15 model-designed peptides were synthesized and their experimental activity against hGLP-1R (**a**) and hGCGR (**b**) measured in triplicate. Here we show the correlation of our predictions (*x* axis) with the experimental results (*y* axis). The identity line (red dashes) illustrates the ideal case where the predicted value exactly matches the experimental measurement. Data points are coloured by the peptide intended activity: yellow, high potency at GCGR and GLP-1R; blue, GLP-1R-selective; green, GCGR-selective. Error bars denote one standard deviation from the mean, computed over three experimental trials (*n* = 3 independent experiments), or the standard deviation from the mean ensemble prediction (*n* = 12 predictions made independently by the neural-network model replicas). **c**, Comparison of the dual co-agonist compounds designed in this work and the training-set data (grey). The designed constructs P1, P2 and P3 exhibit higher dual potency then any variants in the training set. Source data

The 15 model-designed compounds were chemically synthesized, and their experimentally measured potencies were determined using cell-based assays expressing human GLP-1 or glucagon receptors (as described in the Methods and used for experimental evaluation of the peptide training set). Potencies are reported in Table 2 and Supplementary Tables 12 and 13. Overall, we found that the model succeeded in designing peptide analogues with specific quantitative activity. Designs P1–P3 are potent at both GCGR and GLP-1R, and P4 and P5 have an EC_50_ of 68 pM or better at both receptors. This is a striking result given that fewer than 30 training data points have measurements that fall within this range. Notably, our dual-agonist peptides P1, P2 and P3 have up to sevenfold higher potency at both receptors than any other data point in the training set, as shown in Fig. 4c. Our best construct, P3, exhibits 7.2-fold and 8.3-fold potency improvements at hGCGR and hGLP-1R, respectively.

**Table 2:** Multi-task neural-network ensemble-predicted potencies of the 15 designed peptides are compared to their experimentally measured potencies against hGCGR and hGLP-1R (reported in pM)

ID	Gen.	Desired activity category	Predicted GCGR EC_50_ (pM)	Measured GCGR EC_50_ (pM)	Predicted GLP-1R EC_50_ (pM)	Measured GLP-1R EC_50_ (pM)	Sequence
hGCG	–	–	–	1.16	–	206.00	HSQGTFTSDYSKYLDSRRAQDFVQWLMN
hGLP-1	–	–	–	>10,000	–	1.36	HAEGTFTSDVSSYLEGQAAKEFIAWLVKGR
P1	S1	Dual	2.63	1.92	3.09	0.75	**HSQGTFTSDYSKYLDSRAASEFVQWLISH**
T_P1_	S0	–	–	2.08	–	1.59	**HSQGTFTSDYSKYLDSRAASEFVQWLISE**
P2	S3	Dual	0.87	0.18	2.09	2.26	**HSQGTFTSDYSKYLDSRRAYEFVEWLLSG**
T_P2_	S0	–	–	0.83	–	3.16	**HSQGTFTSDYSKYLDSRRASEFVQWLISG**
P3	S3	Dual	0.91	0.12	2.88	0.47	**HSQGTFTSDYSKYLDSRRAYEFVEWLISL**
T_P3_	S0	–	–	0.83	–	3.16	**HSQGTFTSDYSKYLDSRRASEFVQWLISG**
P4	S3	Dual	0.93	5.02	3.09	23.10	**HSQGTFTSDYYKYLDSRRAYEFVEWLISG**
T_P4_	S0	–	–	0.83	–	3.16	**HSQGTFTSDYSKYLDSRRASEFVQWLISG**
P5	S3	Dual	0.98	2.71	2.82	68.00	**HSQGTFTSDYSKYLDSRRAYEFVEWLGSG**
T_P5_	S0	–	–	0.83	–	3.16	**HSQGTFTSDYSKYLDSRRASEFVQWLISG**
P6	S1	GCGR	8.32	>10,000	1174.90	>10,000	**HSQGTFTSDYSKYLDSRRAQDFGQWLEAEE**
T_P6_	S0	–	–	8.31	–	1,621	**HSQGTFTSDYSKYLDSRRAQDFVQWLEAEE**
P7	S3	GCGR	6.61	>10,000	1023.29	>10,000	**HSQGTFTSDYDKYLDSRRAQIFQQWLEAEE**
T_P7_	S0	–	–	8.31	–	1,621	**HSQGTFTSDYSKYLDSRRAQDFVQWLEAEE**
P8	S3	GCGR	6.76	>10,000	1,000.00	>10,000	**HSQGTFTSDYDKYLDSRRAHDQVQWLEAEE**
T_P8_	S0	–	–	8.31	–	1,621	**HSQGTFTSDYSKYLDSRRAQDFVQWLEAEE**
P9	S3	GCGR	6.76	>10,000	1,148.15	>10,000	**HSQGTFTSDYDKYLDSRRAQCFQQWLEAEE**
T_P9_	S0	–	–	8.31	–	1,621	**HSQGTFTSDYSKYLDSRRAQDFVQWLEAEE**
P10	S3	GCGR	6.91	>10,000	1,348.96	>10,000	**HSQGTFTSDYDKYLDSRRAQTFRQWLEAEE**
T_P10_	S0	–	–	8.31	–	1,621	**HSQGTFTSDYSKYLDSRRAQDFVQWLEAEE**
P11	S1	GLP-1R	>10,000	>10,000	6.17	495.00	**HAEGTFTSDVASYLEGQAAKEFIPWLVKGR**
T_P11_	S0	–	–	>10,000	–	1.78	**HAEGTFTSDVASYLEGQAAKEFIAWLVKGR**
P12	S3	GLP-1R	1,122.02	90.53	0.79	2.03	**YSQGTFTSDYSAYLEEEAVRFFINWLLAG**
T_P12_	S0	–	–	10.00	–	1.07	**YSQGTFTSDYSKYLEEEAVRLFIEWLLAG**
P13	S3	GLP-1R	1,148.15	613.33	0.85	5.34	**YSQGTFTSDYSAYLEEEAVRDFITWLLAG**
T_P13_	S0	–	–	10.00	–	1.07	**YSQGTFTSDYSKYLEEEAVRLFIEWLLAG**
P14	S3	GLP-1R	1,258.93	161.00	0.89	2.09	**YSQGTFTSDYSAYLEEEAVRNFIWWLLAG**
T_P14_	S0	–	–	10.00	–	1.07	**YSQGTFTSDYSKYLEEEAVRLFIEWLLAG**
P15	S3	GLP-1R	1,412.54	271.68	0.96	16.37	**YSQGTFTSDYSAYLEEEAVRDFIDWLLAG**
T_P15_	S0	–	–	10.00	–	1.07	**YSQGTFTSDYSKYLEEEAVRLFIEWLLAG**

Peptide designs 6–11 are 30 amino acids long; all other designed peptides contain 29 amino acids. The potencies predicted by the model in log scale (Fig. 4) are transformed here to facilitate comparison with the experimental results. For each sequence, the reported value is an arithmetic mean computed over three independent measurements (Supplementary Tables 12 and 13 provide measurement details). For comparison, the closest training-set sample is listed for each model-designed compound (indicated as ID T_*n*_, Gen 0) together with its measured potencies. Mutated amino acids are highlighted in bold in both sequences.

Peptides P11–P15 were designed to have selective activity at GLP-1R. Here we note that the model’s ability to ablate activity at GCGR was successful, with four of five designs reporting EC_50_ measurements of >161 pM. Of these, four of five designs had measured EC_50_ values of 16.37 pM or better at GLP-1R, and the model also successfully identified that the activity was highly sensitive to amino-acid changes at positions 21–24, which were varied in designs P12–P15. Of these designs, P11 was the least successful, with a measured EC_50_ of 495 pM against GLP-1R.

In contrast, our ability to design peptides selective for GCGR was poor, probably due to model overfitting to the limited available training data. When designing the peptides, we noticed that designs P6–P10 (Extended Data Table 2, green) had lower predicted stability and were significantly more hydrophilic than the other designs. Although the model was successful in that these compounds were inactive at GLP-1R, none of these peptides were active at GCGR. We note that this was the region of the design space for which only four training points were available in our dataset (Figs. 1b and 4c), and the failure of the model to capture this activity probably reflects this paucity of training data.

### Natural peptide analogues

We used our validated multi-task neural-network ensemble to explore whether natural GCG and GLP-1 peptide orthologues found in 288 species (listed in Supplementary Tables 15 and 16) have potency properties aligned with potential therapeutic candidates. Supplementary Fig. 8 shows the predicted potency for each GCG and GLP-1 orthologue at each receptor. We note that all tested natural homologues of GLP-1 are predicted to be inactive at human GCGR, whereas natural glucagon variants have on average around four times higher affinity towards GCGR than towards GLP-1R. These orthologues may provide useful seeds for ML-guided compound design. For example, the glucagon variant from the common degu (*Octodon degus*) is predicted to have high GCGR selectivity—the category that was under-represented in the dataset used to train models in this work—whereas other glucagon variants have high predicted potency against both receptors. These sequences have been subject to the pressures of natural selection, and so may already possess desirable attributes such as optimal biophysical properties and minimal off-target effects, at least in non-human organisms.

## Discussion

In this work we have trained an ensemble of multi-task convolutional neural networks using characterized peptide variants and thus designed and optimized 15 previously uncharacterized helical peptides with specific predicted dual-activity profiles. Our constructs were then synthesized and subjected to experimental verification. Our multi-task neural-network model successfully predicts peptides that exhibit high bioactivity against both receptors or are selective towards GLP-1R. On the other hand, the model fails to predict peptide sequences with selective activity towards glucagon receptors, probably reflecting the paucity of training data points with this selective activity profile.

**Fig. 5:**
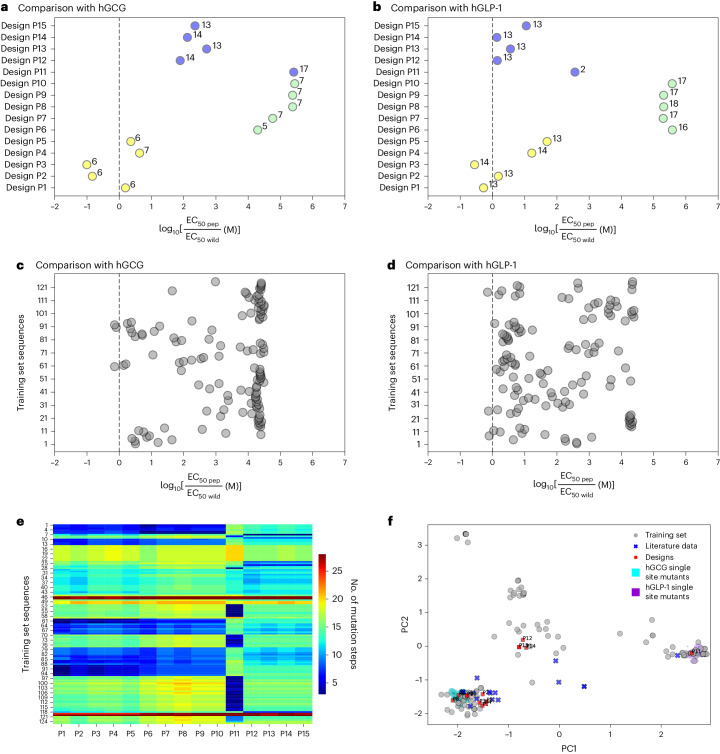
Sequence and potency comparison between the designed peptides, the wild-type human GCG and GLP-1, and the training data. For each peptide, the colour of the marker corresponds to the designated activity group: yellow, dual agonist; green, GCGR-selective; blue, GLP-1R-selective. **a**,**b**, The number of mutation steps from the given wild-type variant hGCG (**a**) and hGLP-1 (**b**) is indicated. Designs P2 and P3 are dual co-agonists, with potency seven- and tenfold higher than human glucagon at hGCGR, and simultaneously P3 has 3.5-fold higher potency than hGLP-1 at hGLP-1R. **c**,**d**, Potency comparison between the training data and human glucagon (**c**) and GLP-1 (**d**), showing that only a few training-set peptides achieved higher potency that the natural ligands, and in all cases the potency improvement is lower than threefold. **e**, Pairwise distances between the model-design peptides (P1–P15, *x* axis) and 125 training-set sequences (*y* axis). The colour of the field corresponds to the distance according to the legend on the right. **f**, PCA projection, as shown in Fig. 2a, with added model-designed compounds (P1–P15) marked in red. Source data

Figure 5 presents a comparison of our model-optimized constructs with the wild-type GPCR-binding ligands—human glucagon and human GLP-1. Only P11 is a close analogue of hGLP-1, with two substitutions at position 11 (S → A) and position 24 (A → P) in the N-terminal and C-terminal helices, respectively. In contrast to our expectation, these two substitutions decrease peptide potency at GLP-1R nearly 400 times, even though, to the best of our knowledge, neither of these positions has been identified as crucial for hGLP-1 activity^30^. This unexpected potency loss may result from the unique properties of proline, which was not seen at position 24 across the training-set examples. Our four remaining GLP-1R-selective constructs—P12–P15—have 13 residues changed with respect to hGLP-1. Our model was able to correctly preserve amino acids at positions 4, 7, 9, 22 and 23, which are known to be important for GLP-1R activation^30^, among all selective peptides (P11–P15), replacing only position 23 with a conservative substitution, I → V, for the dual-agonist peptide designs P1–P5. More importantly, the model specifically targets position 23 in constructs designed to be inactive at GLP-1R (P6–P10, Fig. 5b, marked in green). In addition, the model-imposed aspartate at positions 27, 29 and 30 consistently features in all GLP-1R-inactive peptides. Constructs P1 and P3 have 2- and 3.6-fold higher potency against human GLP-1R than the natural GLP-1 (Fig. 5b). Both of these designs carry a mutation at position 29, recognized to be important for GLP-1R activation by GLP-1^32^, G → H (P1) and G → L (P3). In both cases, these are not isofunctional mutations; moreover, L at position 29 is a novel substitution, introduced by the model.

The comparison with hGCG shown in Fig. 5a indicates that constructs P1–P10 are close hGCG analogues, with five to seven substitutions in the C-terminal region, whereas the GLP-1R-selective peptide designs are further from hGCG in sequence space (blue points). Across peptides that exhibit high potency at both receptors (marked in yellow), changes tend to involve position 20, and positions 27–29 at the C-terminal end. Remarkably, our constructs P2 and P3 are around seven and ten times more potent at hGCGR than natural hGCG, respectively. Several studies indicate the importance of the N terminus for glucagon activity^33,34^. Concordantly, our model tends not to impose changes in this region among peptide analogues designed to exhibit high potency at human GCGR. Among the peptides designed to be selective towards GCGR (marked in green), four have substitutions at position 11 (S → D) and position 23 (V to G, Q or R). Moreover, despite preserving V at position 23, our design P8 contains two new mutations (position 20 H and position 22 Q), which were not seen in the training set (Extended Data Fig. 1). We suspect that these mutations may be responsible for the unexpected loss of peptide affinity towards human GCGR.

Comparison with the training set shows that our conservative sequence design strategy introduces multiple mutations not seen during training (Fig. 5 and Extended Data Fig. 1). For example, our most successful design, P3, contains five mutations, each seen in <20% of training-set sequences, including Y20 (2%) and E24 (6%). Moreover, it adds L at position 29, which was not seen in the training data. Designs P1, P2, P4 and P5, which show high activity at both receptors, also incorporate four to six mutations that were not prevalent among training-set examples, mostly in the C-terminal region of the sequence. Design P14, which satisfies the GLP-1R selectivity criteria, has four low-frequency substitutions and two new mutations introduced at positions 21 and 24, occupied by residues N and W, respectively.

The model-guided search presented in this study enables molecular optimization to improve peptide potency and selectivity. The prediction of peptide in vivo parameters determining biological stability, such as pharmacokinetics (PK) or potential immunogenicity risk would comprise an interesting extension to the presented study with the goal of building general ML-guided modelling pipelines that can result in directly translatable designs.

Remarkably, despite limited training data, our framework achieves three out of five designs in the most desirable space of dual agonists that surpass the best compounds in the training set, with one of our designs (P3) simultaneously improving both potencies by more than sevenfold. Our study showcases the power of ML applied to peptide engineering, demonstrating that sophisticated models can be trained using limited pre-existing datasets to design molecules with significant improvements in functional activity. It is likely that our model’s ability to generalize and make more accurate predictions will improve as more training examples become available, particularly in the missing region of selective peptide activity. Using active learning to collect more data specifically in this region of sequence space and extending the sequence design capabilities by improving model performance is certainly an excellent direction for future studies. This work explores ligand optimization over a restricted design space, constrained by factors such as the limited diversity of the training data and the trust region of the model. As more training data and specifically more diverse training sequences become available, we anticipate that the trained model may accurately extrapolate further in sequence space, relieving these constraints and greatly expanding the corresponding search space to potentially uncover additional sequence design solutions. So far, three G-protein-coupled receptors of the incretin family have long been recognized as key regulators of human metabolism—GCGR, GLP-1R and GIP^11^. The multi-task learning approach presented in this study comprises an exciting opportunity for future research that aims to design triple agonist peptides to tackle metabolic-related pathologies such as obesity or diabetes.

## Methods

### Potency assays

Peptide potencies for cAMP accumulation were experimentally determined for the activation of both hGCGR and hGLP-1R expressed in Chinese hamster ovary (CHO) cells for a set of 125 unique peptide sequence variants, following methods described previously^16,35^. In brief stable CHO cell lines expressing human and mouse GLP-1R were generated in-house using standard methods, as previously described^16^. CHO cells expressing either human GLP-1R or human GCGR were dispensed in assay buffer (Hanks balanced salt solution containing 0.1% BSA (Sigma-Aldrich) and 0.5 mM IBMX (Sigma-Aldrich)) in 384-well assay plates containing dilutions of test peptides. After 30 min of incubation, cAMP levels were measured using the cAMP dynamic 2 HTRF kit (Cisbo) following the manufacturer’s recommendations. Fluorescence emissions at 665 nm and 620 nm following excitation at 320 nm were detected using an Envision reader (Perkin Elmer), and the data were transformed to % Delta F, as described in the manufacturer’s guidelines, before EC_50_ determination. All in vitro cell-based assay data are presented as the mean of *n* ≥ 3 independent experiments, and all individual EC_50_ measurements were within threefold of the geometric mean. The native peptide reference standard potency was within threefold of the historical geometric mean for all assays.

### Datasets

The GPCR-binding peptides considered in this work exclusively comprise naturally occurring amino acids, so the models are not able to capture the effect of any chemical modifications of residues. The initial set of sequences was aligned using MAFFT version 7^36^ to reveal regularities in amino-acid occurrences across positions. We reasoned that sequence alignment might help structure the data, thereby increasing the predictive power of the neural-network models. The aligned sequences were truncated to *L* = 30 amino acids, and redundant sequences were removed. The final set of sequences used in this study comprised *N* = 125 unique peptide sequences tested against human GPCR and GLP-1R receptors. Within this dataset, 122 records were C-terminally amidated. The sequences were subsequently encoded using a one-hot representation and used to train various regression models.

### Data encoding

To encode the amino acid at each sequence position we used a one-hot (binary) representation. Here, we considered 21 categories: 20 amino acids and the gap symbol ‘-’, introduced by alignment. Because nearly all peptides used in these studies (122/125) were C-terminally amidated, we did not introduce an additional parameter to encode this feature. In this approach, each peptide sequence of length *L* is converted to a binary matrix *S* of size 21 × *L*, the entries of which indicate the presence of an amino acid *A*_*i*_ at the given sequence site, such that *S*_*ab*_ = 1 if *a* = *i* and 0 elsewhere, ∀_*b* ∈ {1, …, *L*}_. The binary matrix is then re-shaped into a vector: *S*^21 × *L*^ → *v*^1 × 21*L*^. The alignment process ensures that *L* = 30 for all peptides, such that each sequence is represented by the binary vector ${v\in {{\mathbb{R}}}^{1\times 630}}$.

### Evaluation metrics

We employed the following commonly used regression metrics to evaluate the prediction accuracy of the models developed in this work.

1. Root-mean-square error (r.m.s.e.): ${\rm{r.m.s.e.}}={\sqrt{\frac{1}{N}\mathop{\sum }\nolimits_{i = 1}^{N}{(\;{y}_{i}-\hat{y})}^{2}}}$
2. Mean absolute error (m.a.e.): ${\rm{m.a.e.}}={\frac{1}{N}\mathop{\sum }\nolimits_{i = 1}^{N}| \;{y}_{i}-\hat{y}|}$
3. Coefficient of determination (*R*^2^): ${R}^{2}={1-\frac{\mathop{\sum }\nolimits_{i = 1}^{N}{(\;{y}_{i}-\hat{y})}^{2}}{\mathop{\sum }\nolimits_{i = 1}^{N}{(\;{y}_{i}-\bar{y})}^{2}}}$

Notation: *y*_*i*_, true value of the target for the *i*th sample; ${\hat{y}}$, predicted value of the target for the *i*th sample; ${\bar{y}}$, average value of the target; *N*, number of examples in the batch.

### Neural-network model

We used the Keras/Tensorflow functional API to build the deep network model^37^. The first Conv1D layer in our model has 256 filters, a kernel window of three amino acids, without padding, and an L2 regularization penalty on the kernel, with weight = 0.01. The layer uses ReLU activation. We next added batch normalization and a MaxPool1D operation with stride 2 and used Dropout = 0.5. The second Conv1D layer contains 512 filters and the same configuration of parameters as the first layer, with an additional L2 regularization penalty on the bias term, with weight = 0.01. The layer is activated with ReLU, followed by batch normalization, MaxPool1D operation with stride 2, and Dropout = 0.5. The third convolutional layer has 128 filters; here the padding preserves the shape of the input, and the kernel as well as bias are regularized with L2. This layer is followed by MaxPool1D operation with stride 2. Next, the output from convolutional layers is flattened and two dense layers terminate the network. The first dense layer comprises 256 units, and the second layer has 64 units. Both layers are ReLU-activated. The final two dense layers with a single unit convert the model output to the prediction. These layers are not activated.

### Network ensemble

We constructed a neural-network ensemble model where the final prediction is given by the average of the individual predictions made by *M* = 12 separate copies of the model. Different copies of the model, trained on the same data, differ in their predictions due to factors such as the random initialization of the network parameters. Ensembling predictions over several copies of the model has the effect of mitigating some of this randomness and reducing model variance. The resulting ensemble prediction is given by the average of the ensemble element predictions.

### Model training and hyperparameter tuning

To adjust capacity and select non-trainable model parameters, the available data were used for performance validation. Initially, the dataset of 125 examples was divided into three subsets: 105 training sequences, ten sequences for validation and ten held-out sequences for final model performance evaluation (unseen during training). We performed ten sixfold cross-validations splits with different seeds to split the data, obtaining 60 (test set size) × 10 = 600 data points (errors) in total for each model. Retraining on different data splits allowed us to take into account the variance resulting from training on different data, in addition to the variance that arises due to the random model initialization.

For each baseline model, we used the sklearn grid search (GridSearchCV) to find the set of hyperparameters that provide the best cross-validation performance (listed in Supplementary Table 2). Parameters for which the optimal value differs between tasks are marked with a double value *v*1/*v*2 in the respective column of Supplementary Table 2, where *v*1 is the optimal parameter value for the GCGR task, and *v*2 is the optimal parameter value for the GLP-1R task. For the neural networks, various configurations of layers, unit numbers and regularization were tried, and we selected the model that gave the best performance on the validation set.

In addition, to prevent overfitting of the neural networks, we monitored performance using early stopping. Training was terminated when the optimization loss reported on the validation set goes up after a selected number of parameter updates. Here, we use the Early Stopping monitor implemented in the Keras call-back module^38^. Deep models with 120 training examples (final models) were trained for up to 1,500 epochs, monitoring the validation loss, with the patience of 100 epochs. Each batch for the gradient step contained 25 samples. The deep models with 105 training examples used for validation were trained for up to 1,500 epochs, monitoring the validation loss with the patience of 75 epochs, and 20 examples per batch (each epoch had five parameter updates). Model training is illustrated in Supplementary Fig. 2.

### Baseline models

All baseline regressors in Table 1 were implemented using the sklearn Python module^39^. To confirm that the ML models do not simply learn the underlying potency distributions or amino-acid sequence compositions, we trained control ensembles of multi-task neural networks using the process described above, where we (1) shuffled each peptide sequence used to train the models and (2) shuffled the measured potencies between training examples. The resulting control models make much larger prediction errors; the results are shown in Supplementary Fig. 4 and summarized in Supplementary Table 4. Finally, we implemented a simple nearest-neighbours approach in which the predicted potency for a held-out test sequence is predicted by the measured potency of the nearest neighbour in the training data. For each test sequence we used the *pairwise2* BioPython module with the BLOSUM62 matrix to score alignments with every training sequence; in the case of multiple equidistant training sequences, the average potency was reported. Results across sixfold cross-validation are summarized in Supplementary Table 4 and show that this approach is outperformed by the ML models described above.

We used a *t*-test (two-sided) to test whether the differences in model performance were significant between the ensemble of multi-task neural networks and the other models. Distributions of 600 prediction errors (squared difference between the true and predicted potency for each test sequence) obtained for each model for the GCGR and GLP-1R tasks are shown in Supplementary Table 3. For each pair of models we test the null hypothesis that the two independent populations of error samples have the same average values (we do not assume equal variances). Supplementary Table 3 shows that at a confidence level of 0.05, the multi-task neural-network ensemble performs significantly better in all cases for the GLP-1R task, whereas the performance differences are insignificant in all except one case for the GCGR task.

### Multi-task training

Multi-task learning aims to improve generalization and increase prediction accuracy by learning objectives for several target variables from shared representations^28^. The basic idea is that by training all tasks using shared hidden layers, each task benefits from the presence of the others, which act as regularizers, making the model less sensitive to the specificity of a single target^28,40^. This is because the shared layers are shared representations—the model uses the same weights on each task. The effective number of training examples is therefore increased, and overfitting on each task separately is reduced by optimizing the model with respect to the average data noise^40^.

We used the Kereas deep-learning framework to build our model (https://keras.io) using the TensorFlow back-end^37^. The model consists of eight fully connected layers, comprising the input layer, followed by three 1D convolutional layers, three pooling layers and two dense layers at the bottom of the model, connected to two final units that convert the output to real-valued predictions. The overall objective is the weighted average of the loss for each of the two individual tasks:

1 $${L}_{\rm{total}}={\mathop{\sum }\limits_{i=1}^{k=2}{\alpha }_{i}{L}_{i}}$$

where *L*_*i*_ is the loss function of the *i*th task, *α*_*i*_ is the corresponding weight and *k* denotes the number of tasks. We set *α*_1_ = *α*_2_ = 0.5 so that each loss contributes with equal weight to the overall loss. We use the mean-squared-error (m.s.e.) as the loss for each task, ${\rm{m.s.e.}}={\frac{1}{n}\mathop{\sum }\nolimits_{j = 1}^{n}{(\;{y}_{j}-\hat{y})}^{2}}$, where *n* is the number of training examples per batch.

Our multi-task neural-network model shares all internal hidden layers between the tasks. Two output units return the predicted potencies, ${\hat{y}}_{1}$ and ${\hat{y}}_{2}$. The convolutional layers at the top of the model are designed to encode the peptide representations. We use a kernel with a window size of three amino acids and stride equal to 1. Each convolutional layer is followed by a max pooling layer, with stride equal to 2. We use batch normalization^41^ and Dropout^42^ for regularization. Each convolutional and dense layer is activated with ReLU^43^ activation. We trained the model with an optimization objective as given in equation (1) using the Adam optimizer^44^. The final network was trained on an equal number of training examples for both tasks, *N* = 120.

### Model-guided ligand design

Our goal was to design peptide sequences with the following properties:

- Highly active against both receptors:
  2 $$\begin{array}{r}{\rm{Activity}}=\left\{\begin{array}{l}{\log }_{10}{{{\rm{EC}}}}_{50}^{\rm{GCGR}}[M\,] < {-11.5}\quad \\ {\log }_{10}{{{\rm{EC}}}}_{50}^{\rm{GLP-1R}}[M\,] < {-11.5}\quad \\ {{{\rm{EC}}}}_{50}^{\rm{GCGR}}/{{{\rm{EC}}}}_{50}^{\rm{GLP-1R}}\approx {1}\quad \end{array}\right.\end{array}$$
- Selectively active towards GCGR:
  3 $$\begin{array}{r}{\rm{Activity}}=\left\{\begin{array}{l}{\log }_{10}{{{\rm{EC}}}}_{50}^{\rm{GCGR}}[M\,] < {-11}\quad \\ {\log }_{10}{{{\rm{EC}}}}_{50}^{\rm{GLP-1R}}[M\,] > {-9}\quad \\ {{{\rm{EC}}}}_{50}^{\rm{GCGR}}/{{{\rm{EC}}}}_{50}^{\rm{GLP-1R}}\approx {100}\quad \end{array}\right.\end{array}$$
- Selectively active towards GLP-1R:
  4 $$\begin{array}{r}{\rm{Activity}}=\left\{\begin{array}{l}{\log }_{10}{{{\rm{EC}}}}_{50}^{\rm{GCGR}}[M\,] > {-9}\quad \\ {\log }_{10}{{{\rm{EC}}}}_{50}^{\rm{GLP-1R}}[M\,] < {-11.5}\quad \\ {{{\rm{EC}}}}_{50}^{\rm{GLP-1R}}/{{{\rm{EC}}}}_{50}^{\rm{GCGR}}\approx {100}\quad \end{array}\right.\end{array}$$

We use model-guided directed evolution, an optimization strategy that attempts to solve the optimization problem by imitating the natural evolutionary process. In each successive generation (iteration), a change in the sequence is proposed, followed by the evaluation of a fitness function (here, potency predicted by the ensemble of multi-task neural networks) and the best solutions are progressed to the next generation. This process repeats until a satisfactory solution is reached. In this work we assume that the ensemble of multi-task convolutional neural networks makes reliable predictions up to three mutation steps from the closest training-set analogue sequence.

We first generated all single-step mutations from each training-set sequence in the three groups of interest, removing any duplicates within the generated set, and any overlaps with the training set. Because each sequence in the initial alignment has a length of 30 amino acids and each position can be mutated to one of 19 amino acids (20 if the position is gapped), this gives 570 single-step mutants in the first generation for each sequence in the training set, that is, 71,304 sequences, reducing to 69,639 sequences after removing duplicates. We then used each model to select the 50 best sequences for each of the three target designs defined above, and selected the ten most diverse sequences as starting points for a second round of optimization. Note that in the first generation for the multi-task CNN only five candidate dual agonists were found and used as parents for the second generation. For the second generation we repeated the process described for the first generation, and from the 50 best sequences for each group, we selected five diverse sequences as parents for the third generation. The entire process was then repeated for a final generation, taking the 50 sequences with the best predicted potencies within each of the three groups, considering GCGR for group one.

We identified six biophysical properties that can be predicted from a sequence using the ProtParam module (https://biopython.org/wiki/ProtParam) from the biopython Python package^45^: (1) the isoelectric point in neutral pH, (2) GRAVY (grand average of hydropathy)^46^, (3) the instability index^47^, (4) aromaticity, (5) the molar extinction coefficient and (6) molecular weight. We compared the predicted value for each designed peptide with the predicted properties of peptides in the training set within the same potency group. We ranked the 50 best sequences in each group by computing the number of features whose values are within one standard deviation of the mean calculated for the corresponding group of training-set sequences. As the last step of filtering, we predicted the secondary structure for each final candidate using PSIPRED^48^ (http://bioinf.cs.ucl.ac.uk/psipred/) to confirm that the selected sequences are helical peptides. Using this ranking, we selected five final samples in each potency category—four from the third generation, and one from the first generation of mutants. We prioritized designed sequences with the smallest (first generation) and largest (third generation) distance from the training set. Sequences selected with the ensemble of multi-task neural-network models experimentally tested in this study and discussed in the main text are listed in Table 2 (Supplementary Tables 7–11 provide additional details).

To examine the similarity of peptides predicted by different models within each potency profile, we used PCA, considering the 500 one-hot-encoded sequences generated across all five compared models, the wild-type peptides—hGCG and hGLP-1—and their single-step mutants (551 hGCG and 570 hGLP-1), such that the projection was computed for an array [1,621 × 21*L*]. The projected data are shown in Supplementary Fig. 7. The selected final sequences listed in Supplementary Table 6 were analysed in terms of the total number of mutations from wild-type and predicted potencies. The predictions made with different models are consistent, as evidenced by the low values of standard deviation (<0.5) from the average prediction computed across the models.

To evaluate the information content generated by the sequence design process, we calculated the entropy across each set of designed sequences, and the relative entropy (Kullback–Leibler divergence, KL) between the distribution of amino acids at each sequence position estimated for the model-designed samples, and the training data. KL divergence is equal to zero if and only if the distribution of amino acids across the designed samples matches exactly the respective distribution of amino acids estimated from the training-set sequences. The relative entropy between two discrete distributions *s*(*x*) and *t*(*x*) is given by

5 $${KL(s| | t)}={-\mathop{\sum }\limits_{i=1}^{21}s({x}_{i}){\log }_{21}\frac{t({x}_{i})}{s({x}_{i})}}$$

where *x*_*i*_ is one of the 21 symbols at a selected position *j*. We also measured the dependence between model-generated samples and the training data using mutual information (MI). Given two alignment columns *A* and *B*, each with discrete distributions of amino acids, their MI can be calculated as

6 $${I(A;\,B)}={\mathop{\sum }\limits_{i}^{K=21}\mathop{\sum }\limits_{j}^{L=21}p({x}_{i},\,{y}_{j}){\log }_{21}\frac{p({x}_{i},\,{y}_{j})}{p({x}_{i})p(\;{y}_{j})}={KL(\,p(a,\,b)| | p(a)p(b))}}$$

where *x*_*i*_ is one of the 21 symbols at position *A*, and *y*_*j*_ is one of the 21 symbols at position *B*. The MI describes the reduction of uncertainty about the amino acid at position *i* in our generated samples when we are told what the amino acid at position *i* in the training data is. The higher the value, the more dependent the variables.

### Predicted properties of natural homologues

As described in the main text, we used our multi-task neural-network ensemble model to make predictions for natural GCG and GLP-1 peptide orthologues that are found in various organisms, identified using BLASTp to search the NCBI RefSeq^49^ database to identify non-redundant proglucagon sequences from various organisms across diverse phylogenetic groups. In vertebrates, the pre-proglucagon polypeptide is a product of the GCG gene, which encodes four peptide hormones: glucagon, glucagon-like peptide-1, glucagon-like peptide-2 and oxyntomodulin^50^. In humans, pre-proglucagon has 180 amino acids and is cleaved to yield proglucagon (158 amino acids), which lacks the N-terminal signalling sequence. Proglucagon is subsequently processed by the prohormone convertases 1, 2 and 3^50^ to produce, among other products, the 29-amino-acid-long GCG (in human, positions 53–81, PSCK2) and the 30-amino-acid-long GLP-1_7−36_ (positions 98–127, PSCK1), which are the focus of this work.

We identified 450 initial records, which we aligned using MAFFT version 7^36^ with default parameters to construct a multiple sequence alignment (MSA). We also removed duplicated sequence isoforms, leaving a single representative for each species. Columns with low occupancy (*f* < 30% amino acids) were also removed, leaving 294 unique samples, such that the final MSA contained 294 rows (species) and 179 columns (positions). MSA regions corresponding to the human GCG sequence (positions 53–81) and the GLP-1 human sequence (positions 98–127) were extracted, yielding two sets of corresponding homologues. Species that lacked either a GCG or GLP-1 sequence in the alignment were further removed to yield two final peptide sequence sets, each comprising 288 orthologous sequences. The list of species and NCBI accession numbers, as well as the corresponding peptide sequences, are provided in Supplementary Tables 15 and 16.

### Reporting summary

Further information on research design is available in the Nature Portfolio Reporting Summary linked to this Article.

## Online content

Any methods, additional references, Nature Portfolio reporting summaries, source data, extended data, supplementary information, acknowledgements, peer review information; details of author contributions and competing interests; and statements of data and code availability are available at 10.1038/s41557-024-01532-x.

## Supplementary information

Supplementary Information Supplementary Figs. 1–8, Tables 1–11 and 14.

Reporting Summary

Supplementary Table 12 The potencies of peptides designed in this study by the multi-task neural-network ensemble model against human GCGR were measured in triplicate, in three independent measurements. The results of each experiment are reported (in pM), together with an arithmetic mean *μ* and the standard deviation *σ*.

Supplementary Table 13 The potencies of peptides designed in this study by the multi-task neural-network ensemble model against human GLP-1R were measured in triplicate, in three independent measurements. The results are reported (in pM), together with an arithmetic mean *μ* and the standard deviation *σ*.

Supplementary Table 15 The predictions of multi-task neural-network model ensemble for glucagon orthologues sequences collected from the NCBI database.

Supplementary Table 16 The predictions of multi-task neural-network model ensemble for glucagon-like peptide-1 orthologues sequences collected from the NCBI database.

Supplementary Data 1 Peptide characterization, structures and analytical data.

## Source data

Source Data Fig. 1 Statistical source data.

Source Data Fig. 2 Sequence data.

Source Data Fig. 4 Statistical source data, Dose–response curves.

Source Data Fig. 5 Statistical source data.

Source Data Extended Data Fig./Table 1 Statistical source data.

## Figures and Tables

**Extended Data Table 1:**
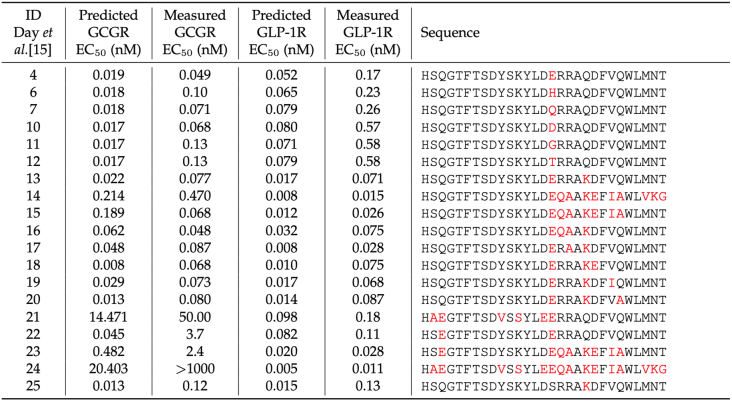
Multi-task ensemble neural-network model predictions on independent literature data

The set of peptides containing natural amino acids for which GCGR and GLP-1R potencies were measured by Day et al.^15^. Here red highlighting indicates sequence mutations from the wild type of human glucagon sequence. The models reported in this paper were not trained on this data, and these sequences are all distinct from those contained in the set used to train our models. We compare the measured activity reported by the authors with predictions made by multi-task ensemble neural-network model developed in this work.

**Extended Data Table 2:**
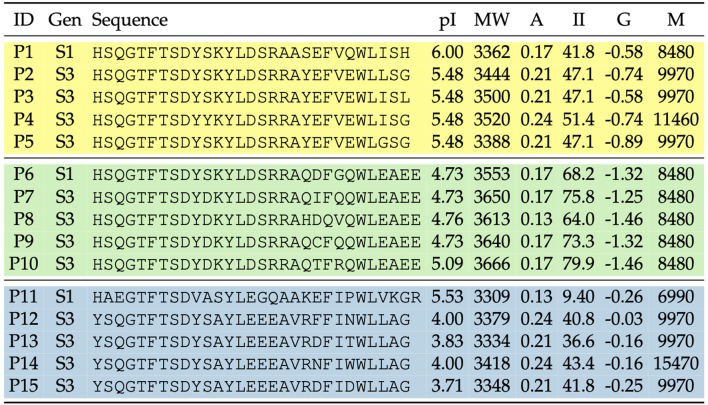
Predicted biophysical properties of designed peptides

Here MW is molecular weight in Daltons, pI is predicted iso- electric point, A is predicted aromaticity, II is predicted instability index (half-life), G is Grand Average of predicted Hydropathy (GRAVY) and M is predicted molar extinction coefficient. The properties were estimated with Prot-Param^45^. Peptide designs 6-11 are 30 amino acids long, while all other designed peptides contain 29 amino acids. The second column indicates the optimization step at which the sequence was collected: S1 denotes the 1st generation and S3 denotes the 3rd generation.

## Data Availability

All data used to build and validate models in addition to all peptide sequences designed and tested in this study are freely available for download from the code repository at https://github.com/amp91/PeptideModels. Source data are provided with this paper.

## Code availability

The pre-trained ensemble of deep multi-task neural-network models with weights used to draw the presented inferences, the code for the ensemble training, and the module for model-guided peptide sequence optimization together with all data used in this study are freely available for download from the online repository https://github.com/amp91/PeptideModels. The repository contains IPython notebooks tutorials that demonstrate how to use the software.
