# Supplementary material for: Machine learning designs new GCGR/GLP-1R dual agonists with enhanced biological potency
Source: Nat Chem. 2024 May 16;16(9):1436–44. doi: 10.1038/s41557-024-01532-x (PMC11374683; doi:10.1038/s41557-024-01532-x)
Supplement: Supplementary file 1 — Supplementary Figs. 1–8, Tables 1–11 and 14. [file 41557_2024_1532_MOESM1_ESM.pdf]

nature chemistry

Article

https://doi.org/10.1038/s41557-024-01532-x

Machine learning designs new GCGR/GLP-1R dual agonists with enhanced biological potency

In the format provided by the
authors and unedited

Supplementary Figures

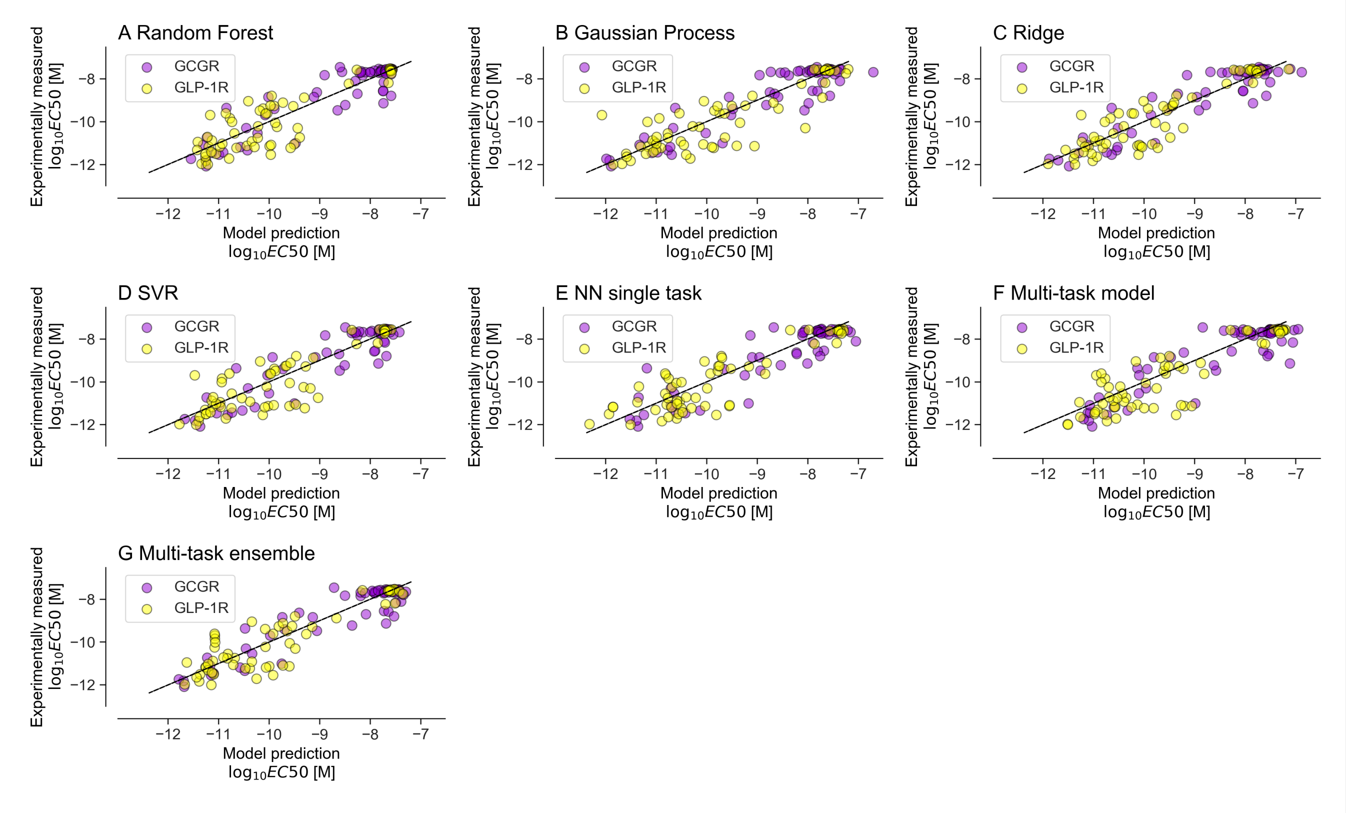

Supplementary Figure 1: Prediction accuracy of (A) Random Forest, (B) Gaussian Process Regression, (C) Ridge Regression, (D) Support Vector Regression and deep Single task (E), Multi-task (F) and Multi-task ensemble (G) neural network models trained in this study to predict peptide potency against GCGR and GLP-1R. In all cases, model performance is evaluated using 6-fold-cross-validation and the average metrics are shown in Table 1 (main text) and Supplementary Table 1. In each fold of the cross-validation, 10 sequences unseen during training are used to test the performance of the model. Each baseline model (Figs. A to D) was fit separately to the available GCGR and GLP-1R training data.

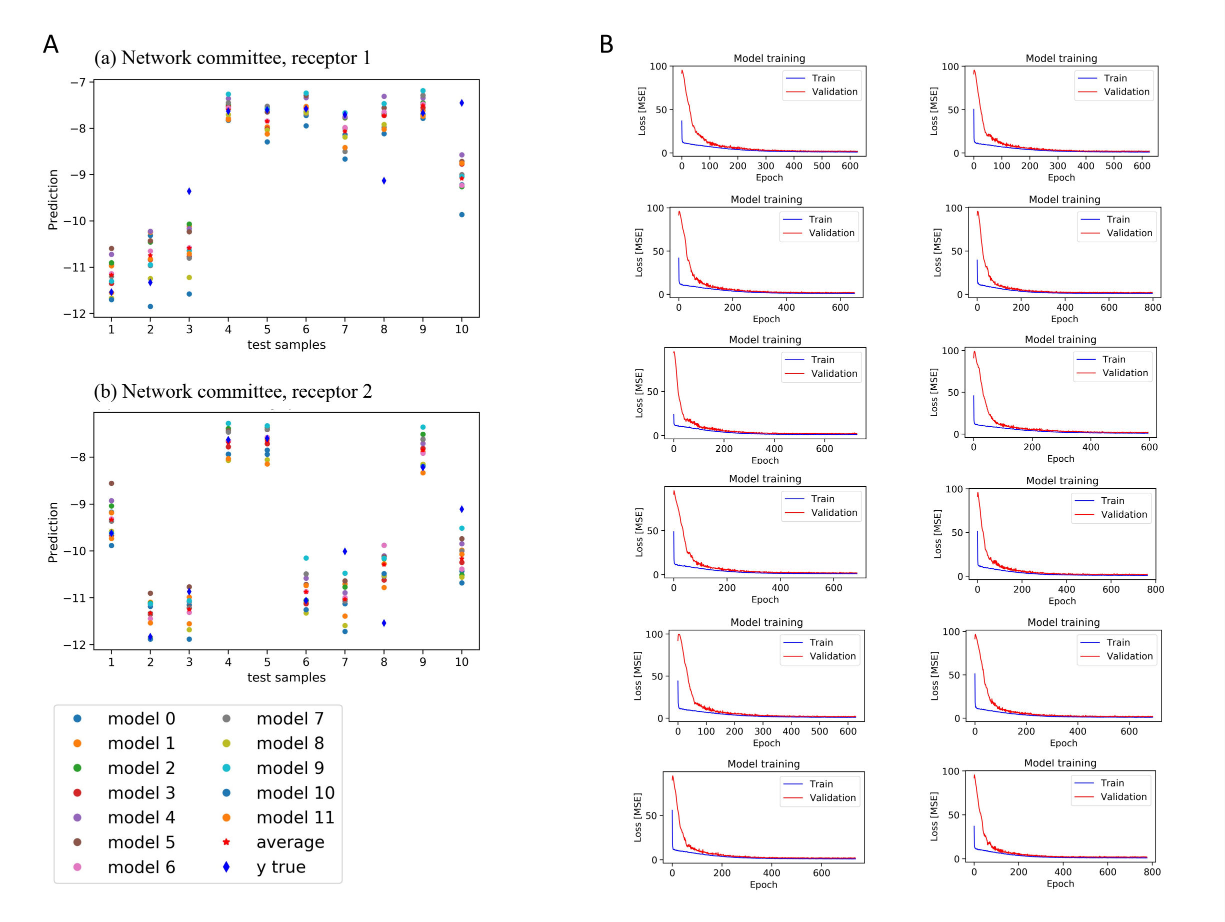

Supplementary Figure 2: Model training. (A) To improve model generalization, we constructed the network ensemble, where the final prediction at each task is given as an average (red asterisk) of the individual predictions made by twelve model replicas (colour circles). After parameter optimisation (training), each model replica makes predictions for both target variables simultaneously. (B) Examples of the training curves for one data batch (fold) across twelve model replicas shown in A. In our multi-task training, the total optimisation loss is the average of the individual losses (see equation 1 in the main text). At each iteration, the model updates parameters to minimize the training set loss (blue curve). To monitor model performance on unseen sequences, we use the performance on the held-out validation set during training (red curves).

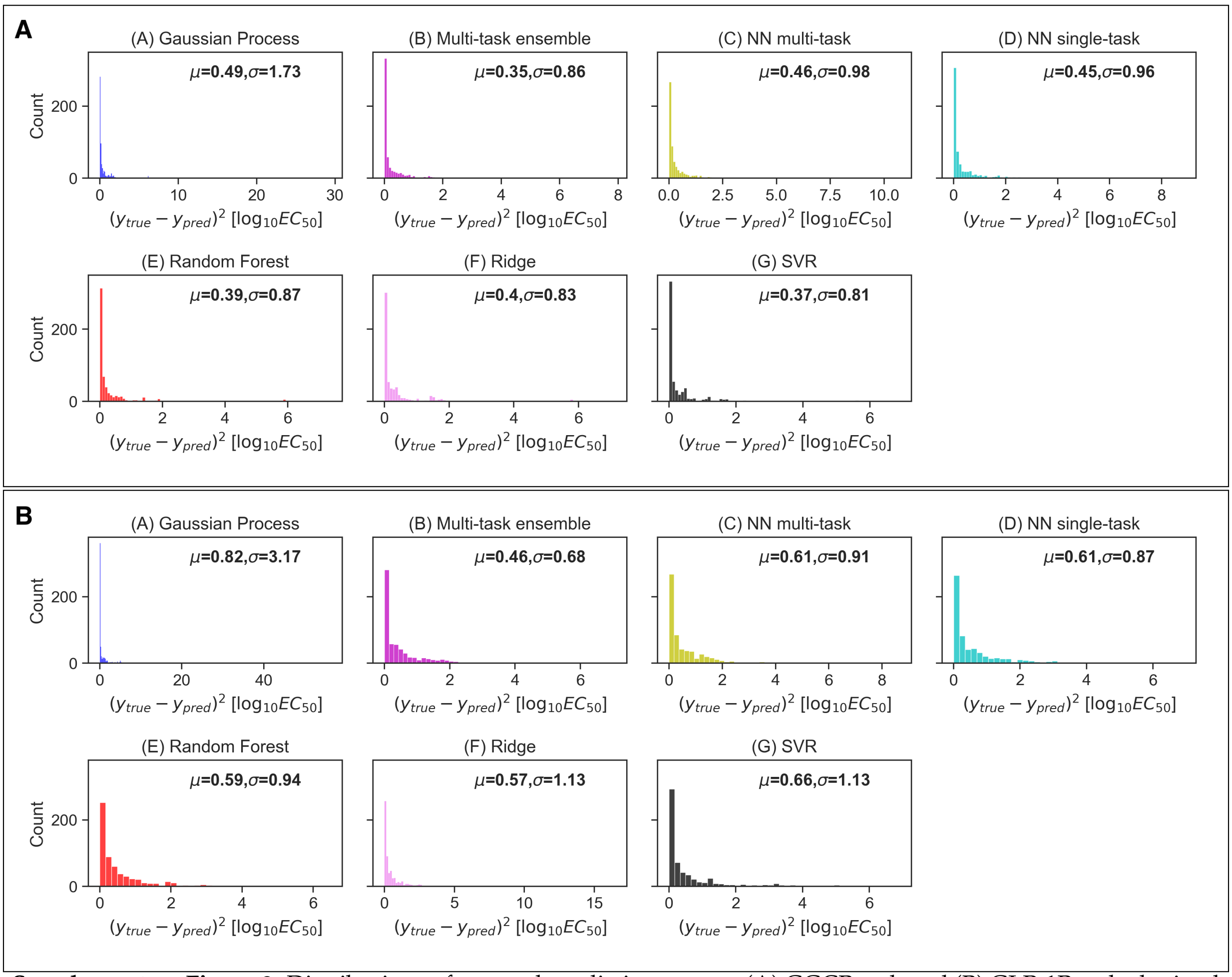

Supplementary Figure 3: Distributions of squared prediction error at (A) GGCR task and (B) GLP-1R task obtained with different machine learning approaches tested in this work.

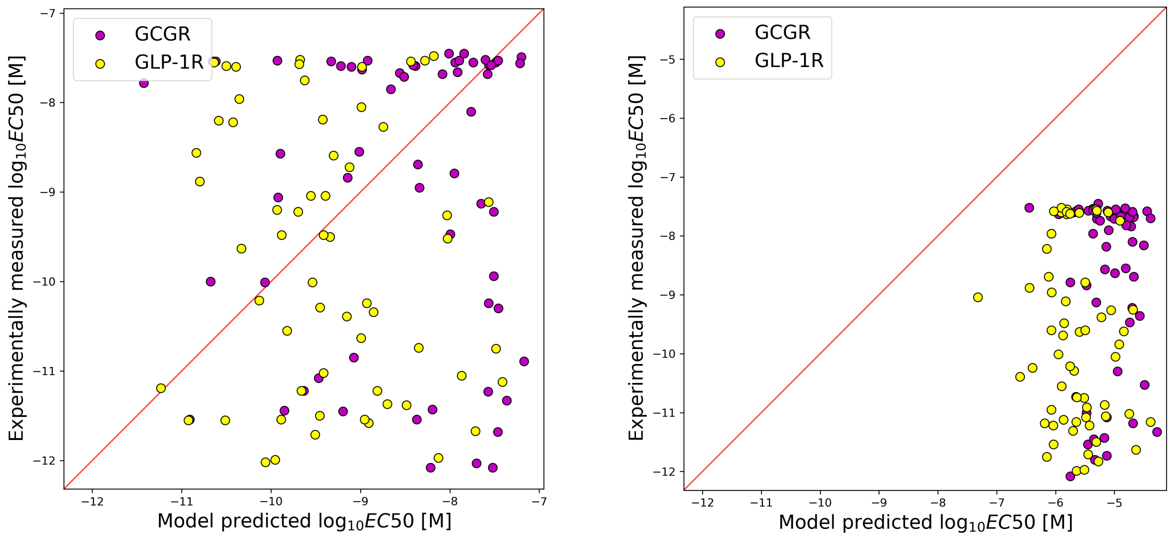

(A) Shuffled targets

(B) Permuted peptide sequences

Supplementary Figure 4: Potency predictions for held-out test sequences made by the ensemble of convolutional multi-task neural network models trained with (A) permuted targets, and (B) permuted peptide sequences.

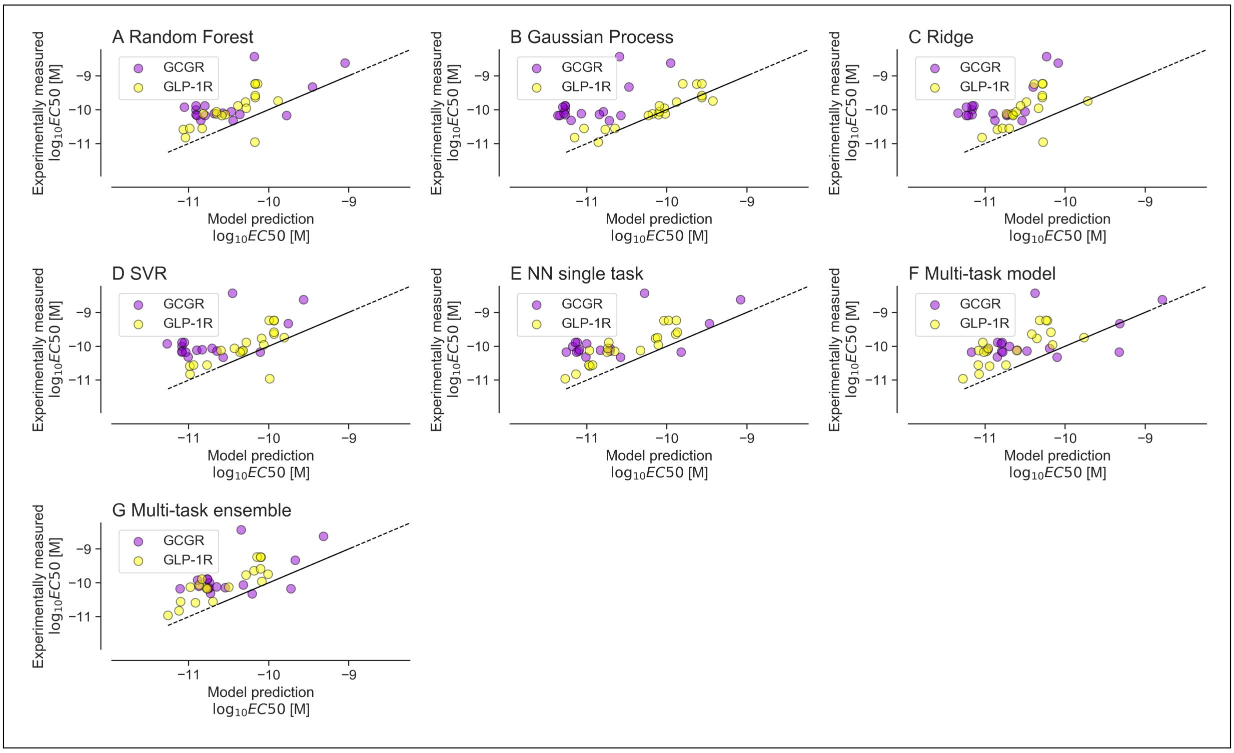

Supplementary Figure 5: Prediction accuracy on independent data from Day *et al.*[15] listed in Extended Data Table 1. (A) Random Forest, (B) Gaussian Process Regression, (C) Ridge Regression, (D) Support Vector Regression, deep Single task (E), Multi-task (F) and Multi-task ensemble (G) models trained in this study to predict peptide potency at GCGR and GLP-1R for literature data (see Extended Data Table 1). Each baseline model (Figs. A to D) was fit separately to the training data discussed in the main text.

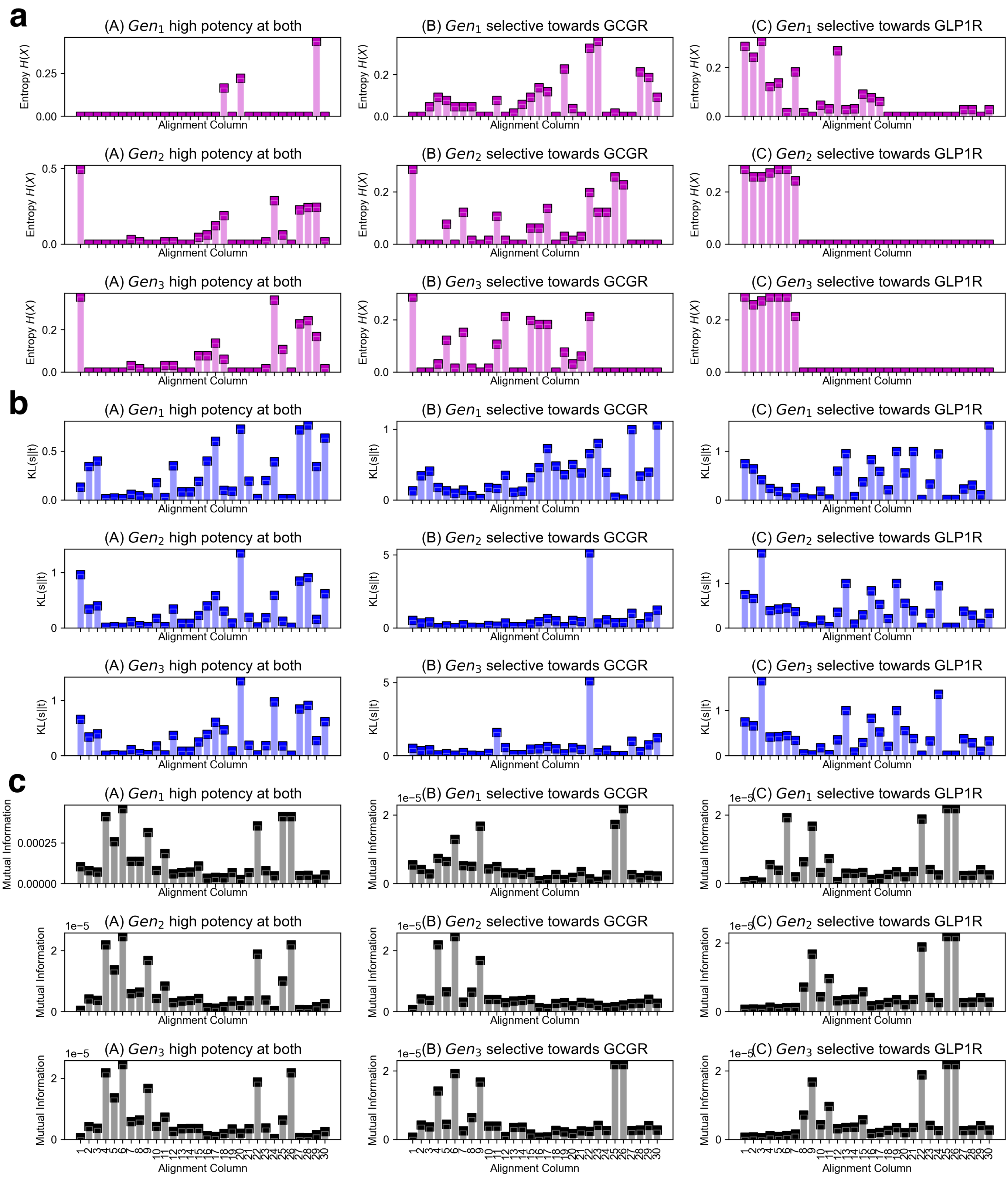

Supplementary Figure 6: (a) Entropy, (b) KL distance and (c) Mutual Information of sequences generated in three rounds (Gen_1, Gen_2, Gen_3) of model-guided optimization by the ensemble of twelve convolutional multi-task neural network models.

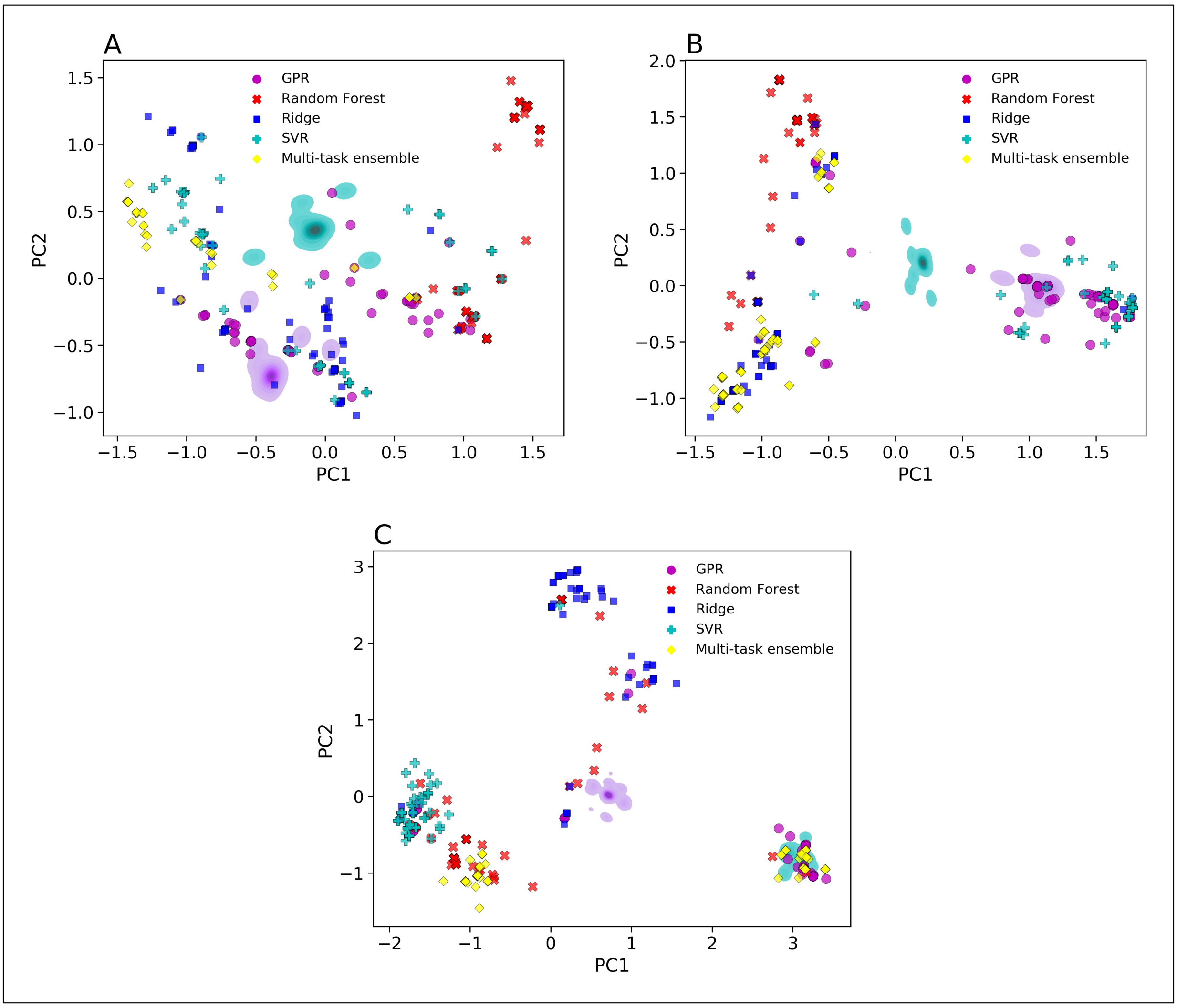

Supplementary Figure 7: Projection of the selected peptide sequences revealed similarity between the designed peptides obtained with different approaches. The projections of data points designed with different models are marked by a colour according to the legend on the left. The single step mutants from human glucagon and GLP-1 are shown for the reference in magenta and cyan, respectively. (A) Similarity of the sequences optimised to achieve dual agonism; 428 sequences projected into its first and second principal components are shown. (B) Projection of sequences optimised towards human GCGR selectivity. Here GPR and SVR predict that this potency category requires closer analogy to human GCG than predicted by other models, possibly due to the adjustment of the potency threshold; 488 projected sequences are shown. (C) Projection of the sequences optimised for hGLP-1R selectivity; 500 data points are shown.

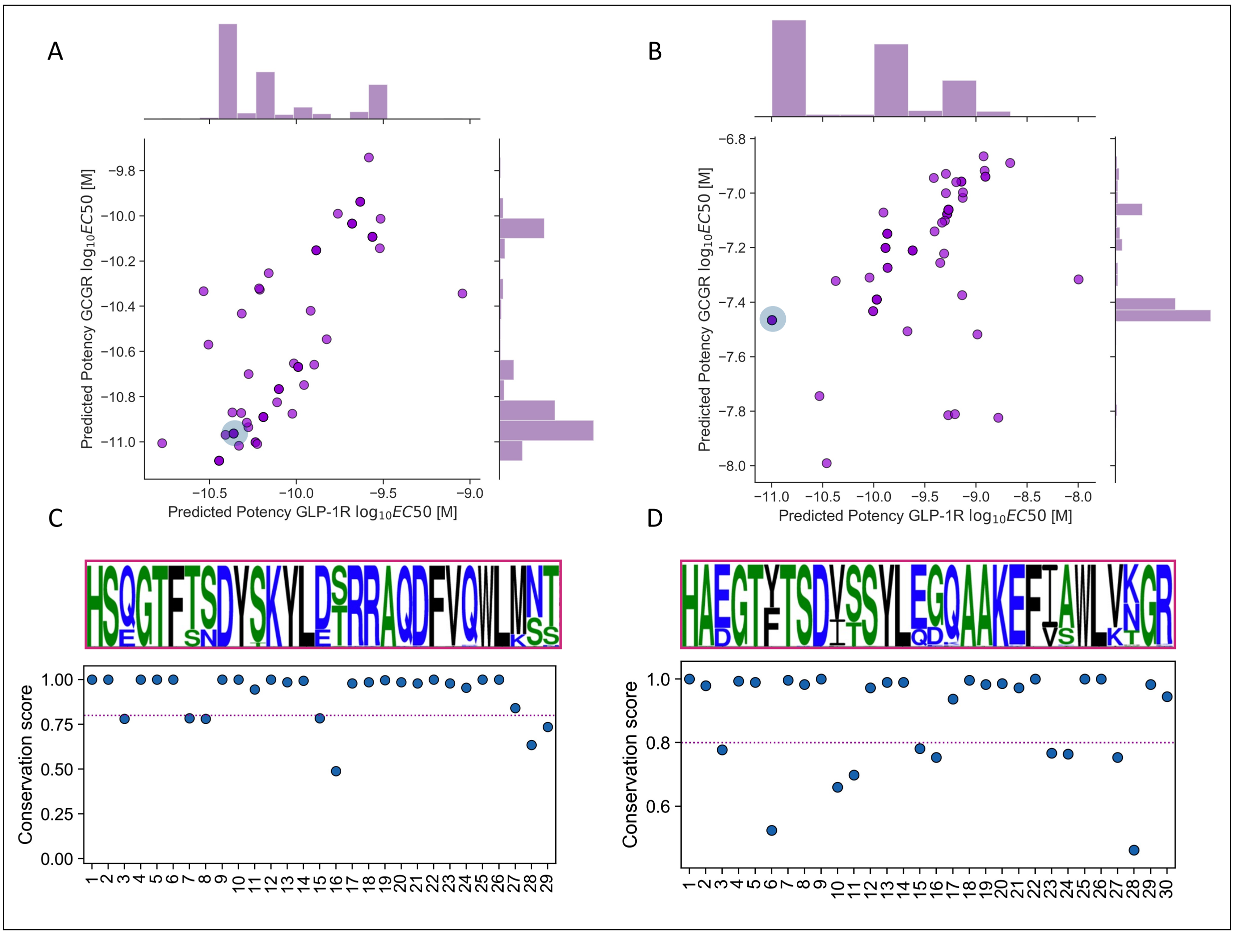

Supplementary Figure 8: Multi-task neural network ensemble activity predictions against human GCGR (y axis) and human GLP-1R (x axis) for natural (A) glucagon and (B) glucagon-like peptide-1 orthologues from 288 species of various organisms (see Supplementary Tables 15 and 16). The predicted potency values for human variants are indicated by blue circles. We observed high correlation (PCC=0.93) between the predicted potencies for glucagon homologues. (C) Evolutionary conservation and sequence logo constructed for the MSA of 288 glucagon variants. (D) Conservation and sequence logo constructed from the MSA of 288 GLP-1 orthologues.

Supplementary Tables

Supplementary Table 1: Model performance comparison. Models predict the EC_{50} measurements for held-out test sequences using 6-fold cross validation, where for each fold 105 sequences are used for training, 10 are used for validation and 10 samples are held-out to measure model performance on the test set.

Models	RMSE		MAE		R^2	
	GCGR	GLP1-R	GCGR	GLP1-R	GCGR	GLP1-R
GP	0.69±0.17	0.88±0.23	0.47±0.05	0.55±0.07	0.75±0.16	0.60±0.27
Random Forest	0.62±0.04	0.77±0.06	0.41±0.02	0.58±0.04	0.81±0.03	0.71±0.05
Ridge	0.63±0.06	0.75±0.10	0.45±0.03	0.58±0.06	0.81±0.04	0.73±0.09
SVR	0.60±0.04	0.81±0.07	0.42±0.02	0.59±0.05	0.82±0.03	0.68±0.06
NN single-task	0.67±0.07	0.78±0.07	0.48±0.04	0.62±0.06	0.78±0.05	0.71±0.06
NN multi-task	0.68±0.06	0.78±0.05	0.49±0.04	0.61±0.04	0.78±0.04	0.71±0.03
NN multi-task ensemble	0.59±0.05	0.68±0.04	0.40±0.02	0.52±0.03	0.83±0.03	0.78±0.03

Supplementary Table 2: Optimal hyperparameters of the baseline models.

**Ridge Regression**		**Support Vector Regression (SVR)**		**Random Forest**		**Gaussian Process Regressor (GPR)**	
parameter	value	parameter	value	parameter	value	parameter	value
alpha	1.5/0.5	C	1.5	bootstrap	FALSE	alpha	0.01
copy_X	TRUE	cache_size	200	ccp_alpha	0	copy_X_train	TRUE
fit_intercept	TRUE	coef0	0	criterion	mae/mse	kernel_length_scale	10
normalize	FALSE/TRUE	degree	3	max_depth	20/10	scale_bounds	(1e-05,1e5)
random_state	42	epsilon	0.1	max_features	sqrt	kernel	RBF(len_s = 10)
solver	lsqr	gamma	scale	min_impurity_decrease	0	n_restarts_optimizer	0
tol	0.001	kernel	poly	min_samples_leaf	1	normalize_y	FALSE
		max_iter	-1	min_samples_split	5/2	optimizer	fmin_l_bfgs_b
		shrinking	TRUE	min_weight_fraction_leaf	0	random_state	42
		tol	0.001	n_estimators	100		
		verbose	FALSE	n_jobs	-1		
				oob_score	FALSE		
				random_state	42		
				warm_start	FALSE		

Supplementary Table 3: T-test (two-sided) results obtained by comparing prediction error populations (see Supplementary Figure 3). The evidence column uses the following p-value categorization: p-value $\geq$0.1 - no evidence to reject the hypothesis, 0.01 $\geq$ p-value $\geq$ 0.05 - weak evidence, 0.05 $\geq$ p-value $\geq$ 0.01 - evidence to reject, 0.01 $\geq$ p-value $\geq$ 0.001 - strong evidence, p-value $\leq$ 0.001 - very strong evidence to reject. No adjustments were made for multiple comparisons.

Task	Statistic	p-value	Compared populations	Test result	Evidence
GCGR	0.677	0.498	Random Forest and Multi-task ensemble	no rejection	no evidence
GCGR	1.774	0.076	Gaussian Process and Multi-task ensemble	no rejection	weak evidence
GCGR	0.872	0.383	Ridge and Multi-task ensemble	no rejection	no evidence
GCGR	0.214	0.831	SVR and Multi-task ensemble	no rejection	no evidence
GCGR	-1.966	0.050	Multi-task ensemble and NN multi-task	reject	weak evidence
GCGR	-1.779	0.076	Multi-task ensemble and NN single-task	no rejection	weak evidence
GLP-1R	2.761	0.006	Random Forest and Multi-task ensemble	reject	strong evidence
GLP-1R	2.683	0.007	Gaussian Process and Multi-task ensemble	reject	strong evidence
GLP-1R	1.962	0.050	Ridge and Multi-task ensemble	reject	weak evidence
GLP-1R	3.604	0.000	SVR and Multi-task ensemble	reject	very strong evidence
GLP-1R	-3.166	0.002	Multi-task ensemble and NN multi-task	reject	strong evidence
GLP-1R	-3.234	0.001	Multi-task ensemble and NN single-task	reject	strong evidence

Supplementary Table 4: Comparison of the performance of adversarial control models with a simple nearest neighbours model and the ensemble of multi-task neural networks.

Models	RMSE		MAE		R^2	
	GCGR	GLP1-R	GCGR	GLP1-R	GCGR	GLP1-R
Shuffled targets	1.99	1.92	1.50	1.62	-0.51	-0.65
Permuted peptide sequences	3.87	4.46	3.55	4.16	-5.98	-8.47
Nearest-neighbours	0.79±0.04	1.42±0.03	0.48±0.02	0.97±0.02	0.72±0.03	0.08±0.04
NN multi-task ensemble	0.59±0.05	0.68±0.04	0.40±0.02	0.52±0.03	0.83±0.03	0.78±0.03

Supplementary Table 5: Model performance comparison on literature data from Day *et al.*[15].

Models	MAE		R^2	
	GCGR	GLP1-R	GCGR	GLP1-R
Ridge	1.04±NA	0.56±NA	0.02±NA	-0.59±NA
SVR	0.96±NA	0.38±NA	0.08±NA	0.2±NA
GPR	1.17±NA	0.19±NA	-0.22±NA	0.76±NA
Random Forest	0.84±0.06	0.56±0.06	0.24±0.08	-0.51±0.3
NN single-task	0.80±0.16	0.36±0.13	0.32±0.24	0.29±0.37
NN multi-task	0.87±0.12	0.68±0.37	0.15±0.13	-1.44±1.78
NN multi-task ensemble	0.73±0.02	0.46±0.09	0.42±0.02	-0.12±0.32

Supplementary Table 6 caption: Predictions for optimised sequences, designed with different models studied in this work. Sequences 61-75 (highlighted) predicted by the multi-task neural network ensemble model were synthesized and their potency experimentally evaluated (see in main text). The number of point mutation steps between the designed sequence and the natural ligands: human GCG and GLP-1 is listed for each record. The predicted effect of substitutions is computed as a difference between the potency predicted for the given peptide, and the measured potency of the natural ligand, reported in the log scale. Negative values correspond the potency increase with respect to the wild-type peptide.

Supplementary Table 6

			Predicting model	Multi-task ensemble		RF		GPR		Ridge		SVR		**Prediction at hGCGR**		**Prediction at hGLP-1R**						
ID	Gen	Proposing Model	Sequence	GCGR EC50	GLP-1R EC50	GCGR EC50	GLP-1R EC50	GCGR EC50	GLP-1R EC50	GCGR EC50	GLP-1R EC50	GCGR EC50	GLP-1R EC50	Average prediction	STD	Average prediction	STD	Distance to human glucagon	Potency difference	Distance to human GLP-1	Potency difference	Design group
1	1	GPR	HSQGTFTSDYSKYLDSRRASEFVQWLISD-	-11.67	-11.20	-11.79	-11.27	-11.72	-11.04	-11.76	-11.41	-11.53	-11.15	-11.69	0.10	-11.21	0.14	5	0.18	15	0.54	g1
2	3	GPR	HSQGTFTSDYSKYLDSRRASEFVQWLIDG-	-11.61	-11.24	-11.71	-11.38	-11.68	-11.06	-11.65	-11.26	-11.34	-11.19	-11.60	0.15	-11.23	0.11	5	0.22	14	0.52	g1
3	3	GPR	HSQGTFTSDYSRYLDSIRASEFVQWLISE-	-11.59	-11.13	-11.69	-11.23	-11.63	-11.06	-11.62	-11.37	-11.11	-11.01	-11.53	0.23	-11.16	0.14	7	0.27	15	0.52	g1
4	3	GPR	HSQGTFTSDYSKYLDSRAAREFVQWLISE-	-11.67	-11.41	-11.49	-11.58	-11.71	-11.30	-11.86	-11.53	-11.44	-11.39	-11.63	0.17	-11.44	0.11	6	0.19	14	0.28	g1
5	3	GPR	HSQGTFTSDYSKYLDSRRASEFVQWLISH-	-11.73	-11.28	-11.79	-11.27	-11.71	-11.04	-11.70	-11.35	-11.52	-11.14	-11.69	0.10	-11.22	0.13	5	0.19	15	0.54	g1
6	1	GPR	HSQGTFTSDYDKYLDSRRAESFVQWLENT-	-11.40	-9.74	-11.29	-9.68	-11.17	-9.85	-13.00	-10.22	-11.07	-10.10	-11.59	0.80	-9.92	0.23	4	0.73	17	1.73	g2
7	3	GPR	HSQGTFTSDYSKYLDSRRAQDFVQWLESEG	-11.29	-9.32	-11.28	-9.51	-11.35	-9.84	-11.06	-9.67	-11.03	-10.15	-11.20	0.14	-9.70	0.32	4	0.55	16	1.74	g2
8	3	GPR	HSQGTFTSDYSKYLDSRRAEDFVQWLENE-	-11.32	-9.49	-11.27	-9.56	-11.39	-9.69	-11.54	-9.60	-11.40	-10.02	-11.38	0.10	-9.67	0.21	3	0.51	16	1.89	g2
9	3	GPR	HSQGTFTSDYSKYLDSRRARDFVQWLENT-	-11.38	-10.29	-11.22	-9.68	-11.54	-9.94	-11.62	-10.03	-11.37	-10.26	-11.42	0.16	-10.04	0.25	2	0.36	16	1.64	g2
10	3	GPR	HSQGTFTSDYSKYLDSRRAEEFVQWLENT-	-11.46	-10.13	-11.48	-9.68	-11.58	-9.98	-11.76	-9.90	-11.36	-10.26	-11.53	0.15	-9.99	0.22	3	0.32	15	1.60	g2
11	1	GPR	HAEGTFTADVSDYLEGQAAKEFIAWLVKR-	-8.32	-11.14	-7.93	-11.54	-8.07	-11.19	-7.96	-12.45	-8.08	-9.72	-8.07	0.15	-11.21	0.98	16	3.83	4	0.39	g3
12	3	GPR	YSEGTFTSDTSKLLEEEAVRDFIEWLLAGG	-8.52	-11.44	-8.97	-11.80	-8.99	-11.24	-8.95	-11.96	-8.76	-11.29	-8.84	0.20	-11.54	0.32	16	2.91	14	0.34	g3
13	3	GPR	YSEGTFTSDKSKLLEEEAVRDFIEWLLAGG	-8.45	-11.35	-8.97	-11.80	-8.99	-11.24	-8.95	-11.96	-8.76	-11.29	-8.82	0.23	-11.53	0.33	16	2.91	14	0.34	g3
14	3	GPR	HSQGTFTSDYSEYLDSEHARDFVQWLVAGG	-8.40	-11.03	-9.36	-11.40	-8.97	-11.00	-8.95	-11.46	-9.42	-11.15	-9.02	0.41	-11.21	0.21	8	2.93	14	0.58	g3
15	3	GPR	YSEGTFTSDASKLLEEEAVRDFIEWLLAGG	-8.38	-11.56	-8.97	-11.80	-8.99	-11.24	-8.95	-11.96	-8.76	-11.29	-8.81	0.26	-11.57	0.31	16	2.91	14	0.34	g3
16	1	Ridge	HSQGTFTSDNSKYLDSRRAYEFVIWLIAE-	-11.70	-11.29	-11.64	-11.17	-11.24	-10.30	-12.79	-11.81	-10.99	-10.73	-11.67	0.69	-11.06	0.57	7	-0.89	15	-0.23	g1
17	3	Ridge	HSQGTFTSDYEKYLDSRRASEFVQWLISG-	-11.34	-10.95	-11.81	-11.39	-11.57	-10.92	-12.08	-11.62	-11.28	-11.21	-11.62	0.33	-11.22	0.29	6	-0.18	15	-0.04	g1
18	3	Ridge	HSQGTFTSDYDKYLDSRRASEFVQWLISG-	-11.82	-11.23	-11.81	-11.39	-11.75	-10.92	-13.07	-11.62	-11.31	-11.24	-11.95	0.66	-11.28	0.25	6	-1.17	15	-0.04	g1
19	3	Ridge	HSQGTFTSDYDKYLDSERARDFVAWLVAGG	-11.03	-11.36	-11.08	-11.49	-10.93	-10.96	-12.21	-11.66	-10.09	-11.21	-11.07	0.75	-11.34	0.27	8	-0.31	14	-0.08	g1
20	3	Ridge	HSQGTFTSDYSRYLDSRRASEFVIWLISE-	-11.71	-11.29	-11.66	-11.28	-11.51	-10.85	-12.12	-11.81	-11.13	-11.04	-11.63	0.36	-11.25	0.36	7	-0.22	15	-0.23	g1
21	1	Ridge	HSQGTFTSDYGKQLDSRRAQYFVQWLEAEE	-10.61	-8.80	-11.17	-9.07	-10.57	-9.61	-11.84	-8.90	-10.56	-9.62	-10.95	0.56	-9.20	0.39	7	0.06	18	2.68	g2
22	3	Ridge	HSQGAFTSDYSKYLDSRRAQDFVQWLEEGE	-11.04	-9.07	-11.24	-9.24	-10.95	-9.47	-11.31	-8.55	-10.63	-9.63	-11.03	0.27	-9.19	0.41	5	0.59	16	3.03	g2
23	3	Ridge	HSQGCFTSDYSKYLDSRRAQDFVQWLEEGE	-11.11	-9.12	-11.24	-9.24	-10.95	-9.47	-11.31	-8.55	-10.63	-9.63	-11.05	0.27	-9.20	0.41	5	0.59	16	3.03	g2
24	3	Ridge	HSQGMFTSDYSKYLDSRRAQDFVQWLEEGE	-11.07	-9.10	-11.24	-9.24	-10.95	-9.47	-11.31	-8.55	-10.63	-9.63	-11.04	0.27	-9.20	0.41	5	0.59	16	3.03	g2
25	3	Ridge	HSQGTFGSDYSKYLDSRRAQDFVQWLEAEE	-11.07	-9.00	-11.16	-8.74	-10.86	-9.21	-11.29	-8.94	-10.76	-9.50	-11.03	0.22	-9.08	0.29	5	0.61	17	2.64	g2
26	1	Ridge	YSQGTLTSDYSCLLERQAIDEFVNWLLKGG	-8.25	-11.02	-10.19	-11.59	-8.90	-10.44	-8.96	-11.51	-8.68	-10.92	-9.00	0.72	-11.10	0.47	16	2.94	14	0.07	g3
27	3	Ridge	YSEGTFTSDYSKLLERMAIDEFVNWLLKGG	-8.40	-11.26	-8.86	-11.59	-8.81	-11.09	-9.00	-11.66	-8.71	-11.12	-8.75	0.23	-11.34	0.27	15	2.90	13	-0.08	g3
28	3	Ridge	YSEGTFTSDYSKLLERPAIDEFVNWLLKGG	-8.33	-11.21	-8.86	-11.59	-8.81	-11.09	-9.00	-11.66	-8.71	-11.12	-8.74	0.25	-11.33	0.27	15	2.90	13	-0.08	g3
29	3	Ridge	YSEGTFTSDYSKLLERNAIDEFVNWLLKGG	-8.34	-11.28	-8.86	-11.59	-8.81	-11.09	-9.00	-11.66	-8.71	-11.12	-8.74	0.25	-11.35	0.26	15	2.90	13	-0.08	g3
30	3	Ridge	YSEGTFTSDYSKLLERWAIDEFVNWLLKGG	-8.29	-11.12	-8.86	-11.59	-8.81	-11.09	-9.00	-11.66	-8.71	-11.12	-8.73	0.27	-11.31	0.28	15	2.90	13	-0.08	g3
31	1	Random Forest	HSQGTFTSDYSKYLDSRAASEWVQWLISE-	-11.44	-11.14	-11.59	-11.58	-11.23	-11.09	-11.43	-11.24	-11.21	-11.18	-11.38	0.16	-11.25	0.19	7	0.31	15	0.44	g1
32	3	Random Forest	HSQGTFTTDYSKYLDSRAASEFVQWLISE-	-11.35	-11.09	-11.59	-11.58	-11.22	-11.09	-11.40	-11.63	-11.21	-11.18	-11.35	0.16	-11.31	0.27	7	0.31	15	0.55	g1
33	3	Random Forest	HSQGTFTSDYQKYLDSRAASEFVQWLISE-	-11.43	-11.13	-11.59	-11.58	-11.29	-11.09	-11.83	-11.68	-11.22	-11.23	-11.47	0.24	-11.34	0.27	7	0.31	15	0.51	g1
34	3	Random Forest	HSQKTFTSDYSKYLDSRAASEFVQWLISE-	-11.21	-10.97	-11.59	-11.58	-11.22	-11.09	-11.37	-11.14	-11.20	-11.18	-11.32	0.17	-11.19	0.23	7	0.31	15	0.58	g1
35	3	Random Forest	HSQGGFTSDYSKYLDSRAASEFVQWLISE-	-11.49	-11.26	-11.59	-11.58	-11.25	-11.09	-11.63	-10.84	-11.22	-11.14	-11.44	0.19	-11.18	0.27	7	0.31	15	0.58	g1
36	1	Random Forest	HSQGTFKSDYSKYLDSRRAQEFVQWLEELE	-11.08	-9.34	-11.43	-8.86	-11.01	-9.72	-11.70	-9.34	-10.71	-9.67	-11.19	0.39	-9.39	0.34	6	0.42	17	1.64	g2
37	3	Random Forest	HSQGTFASDYSKYLDSRRAQDFVQWLEEGE	-11.01	-9.07	-11.24	-8.92	-10.93	-9.46	-11.33	-9.17	-10.61	-9.56	-11.02	0.28	-9.24	0.27	5	0.67	16	1.60	g2
38	3	Random Forest	HSQGTFCSDYSKYLDSRRAQDFVQWLEEGE	-11.16	-9.15	-11.24	-8.92	-10.93	-9.46	-11.31	-9.14	-10.62	-9.56	-11.05	0.28	-9.25	0.26	5	0.67	16	1.60	g2
39	3	Random Forest	HSQGTFGSDYSKYLDSRRAQDFVQWLEAEE	-11.07	-9.00	-11.16	-8.74	-10.86	-9.21	-11.29	-8.94	-10.76	-9.50	-11.03	0.22	-9.08	0.29	5	0.70	17	1.63	g2
40	3	Random Forest	HSQGTFSSDYSKYLDSRRAQDFVQWLEAEE	-11.08	-9.01	-11.16	-8.74	-10.85	-9.20	-11.30	-8.71	-10.76	-9.44	-11.03	0.22	-9.02	0.31	5	0.70	17	1.68	g2
41	1	Random Forest	YSAGTFTSDYSKLLEEESIRLFIEWLVKGP	-7.92	-10.71	-9.27	-11.22	-8.74	-10.26	-8.18	-9.86	-8.75	-10.67	-8.57	0.53	-10.55	0.51	16	3.13	16	0.45	g3
42	3	Random Forest	YSPGTFTSDYSKLLERQAIDEFVNWLLKGG	-8.51	-11.58	-9.26	-11.61	-8.92	-11.05	-8.93	-11.60	-8.77	-11.14	-8.88	0.27	-11.40	0.28	15	2.98	14	0.30	g3
43	3	Random Forest	YSEGTFTSDYSKLLEREAVRDFIEWLLAGG	-8.92	-11.70	-9.04	-11.79	-9.27	-11.33	-9.60	-12.30	-9.01	-11.47	-9.17	0.28	-11.72	0.37	15	3.01	14	0.18	g3
44	3	Random Forest	HSEGTFTSDYSKYLEEEAVRLFICWLMNT-	-10.01	-11.27	-9.23	-11.60	-9.68	-11.01	-9.67	-11.76	-9.75	-11.06	-9.67	0.28	-11.34	0.33	10	2.92	15	0.25	g3
45	3	Random Forest	HSEGTFTSDYSKYLDRAAASDFVQWLVAGG	-9.68	-11.15	-9.03	-11.28	-9.35	-10.88	-9.21	-11.58	-9.25	-11.06	-9.30	0.24	-11.19	0.26	9	2.95	14	0.30	g3
46	1	SVR	HSQGTFTSDYSKYLDSRAASEFVWQLISE-	-11.57	-11.31	-11.58	-11.59	-11.07	-10.72	-11.97	-12.00	-11.03	-11.14	-11.44	0.40	-11.35	0.48	8	0.87	15	0.44	g1
47	3	SVR	HSQGTFTSDYSKYLDSRRASEFVQWLIGE-	-11.65	-11.17	-11.74	-11.17	-11.66	-10.91	-11.70	-10.86	-11.58	-11.03	-11.67	0.06	-11.03	0.14	5	0.32	15	0.55	g1
48	3	SVR	HSQGTFTSDYSKYLDSRRAAEFVQWLISE-	-11.68	-11.22	-11.71	-11.13	-11.82	-10.89	-12.08	-10.91	-11.65	-11.07	-11.79	0.18	-11.04	0.14	5	0.25	15	0.51	g1
49	3	SVR	HSQGTFTSDYSKYLDSRRALEFVQWLISE-	-11.63	-11.06	-11.71	-11.15	-11.80	-10.84	-12.11	-10.88	-11.64	-11.00	-11.78	0.20	-10.99	0.13	5	0.26	15	0.58	g1
50	3	SVR	HSQGTFTSDYSKYLDSRRANEFVQWLISE-	-11.62	-11.12	-11.71	-11.15	-11.80	-10.84	-12.11	-10.88	-11.64	-11.00	-11.78	0.20	-11.00	0.14	5	0.26	15	0.58	g1
51	1	SVR	HSQGTFTSDYSKYLDSRRECDFVQWLENT-	-11.10	-9.17	-11.20	-9.65	-10.99	-9.84	-11.00	-8.89	-11.04	-9.94	-11.07	0.09	-9.50	0.45	3	0.86	17	1.64	g2
52	3	SVR	HSQGTFTSDYSKYLDSRRAEDFVPWLENT-	-11.28	-9.75	-11.21	-9.68	-11.23	-9.75	-11.72	-10.01	-11.09	-9.98	-11.31	0.24	-9.83	0.15	3	0.81	16	1.60	g2
53	3	SVR	HSQGTFTSDYSKYLDSRRAEDFVLWLENT-	-11.27	-9.67	-11.21	-9.68	-11.23	-9.75	-11.72	-10.01	-11.09	-9.98	-11.30	0.24	-9.82	0.17	3	0.81	16	1.60	g2
54	3	SVR	HSQGTFTSDYYKYLDSRRAEDFVQWLENT-	-11.25	-9.57	-11.22	-9.68	-11.06	-9.70	-11.78	-10.20	-11.08	-9.95	-11.28	0.29	-9.82	0.25	3	0.82	17	1.63	g2
55	3	SVR	HSQGTFTSDYSKYLDSRRAEDAVQWLENT-	-11.27	-9.52	-11.22	-9.68	-11.01	-9.70	-11.38	-9.76	-11.08	-9.90	-11.19	0.15	-9.71	0.14	3	0.82	17	1.68	g2
56	1	SVR	YCQGTFTSDYSKLLEEEAVRDVIEWLLAGG	-8.66	-11.25	-9.14	-9.05	-9.15	-10.62	-8.56	-10.69	-8.94	-11.13	-8.89	0.27	-10.55	0.88	16	2.96	16	0.45	g3
57	3	SVR	YSEGTFTSDYSKLLEEEAVRDFIEWLLAGD	-8.73	-11.64	-9.12	-11.78	-9.24	-11.35	-9.30	-11.61	-8.98	-11.28	-9.07	0.23	-11.53	0.21	15	2.92	14	0.30	g3
58	3	SVR	QSEGTFTSDYSKLLEEEAVRDFIEWLLAGG	-8.83	-11.78	-9.04	-11.81	-9.14	-11.27	-9.19	-12.10	-8.96	-11.40	-9.03	0.14	-11.67	0.33	15	2.94	14	0.18	g3
59	3	SVR	YSEGTFTSDYSKLLEEEAVRDFIEWLLANG	-8.69	-11.56	-9.04	-11.79	-9.13	-11.24	-9.14	-12.14	-8.97	-11.33	-8.99	0.18	-11.61	0.37	15	2.93	15	0.25	g3
60	3	SVR	YSEGTFTSDYSKLLEEEAVRDFIEWLLAGT	-8.75	-11.67	-9.12	-11.78	-9.24	-11.35	-9.38	-11.61	-8.99	-11.28	-9.10	0.24	-11.54	0.21	15	2.91	14	0.30	g3
61	1	MT ensemble	HSQGTFTSDYSKYLDSRAASEFVQWLISH-	-11.58	-11.51	-11.49	-11.61	-11.42	-11.32	-11.29	-11.77	-11.21	-11.33	-11.40	0.15	-11.51	0.19	6	0.16	13	-0.57	g1
62	3	MT ensemble	HSQGTFTSDYSKYLDSRRAYEFVEWLLSG-	-12.06	-11.68	-11.53	-11.43	-11.84	-10.58	-13.22	-11.93	-11.14	-11.20	-11.96	0.79	-11.37	0.52	6	-0.87	13	-0.11	g1
63	3	MT ensemble	HSQGTFTSDYSKYLDSRRAYEFVEWLISL-	-12.04	-11.54	-11.69	-11.28	-11.70	-10.50	-13.03	-11.75	-11.29	-10.97	-11.95	0.66	-11.21	0.49	6	-1.04	14	-0.84	g1
64	3	MT ensemble	HSQGTFTSDYYKYLDSRRAYEFVEWLISG-	-12.03	-11.51	-11.74	-11.38	-11.58	-10.44	-13.41	-12.10	-11.08	-11.05	-11.97	0.88	-11.30	0.61	7	0.60	14	0.93	g1
65	3	MT ensemble	HSQGTFTSDYSKYLDSRRAYEFVEWLGSG-	-12.01	-11.55	-11.58	-11.38	-11.72	-10.50	-12.98	-11.67	-11.08	-11.03	-11.87	0.70	-11.23	0.47	6	0.32	13	1.40	g1
66	1	MT ensemble	HSQGTFTSDYSKYLDSRRAQDFGQWLEAEE	-11.08	-8.93	-11.16	-9.04	-10.86	-9.22	-11.11	-8.88	-10.76	-9.63	-10.99	0.17	-9.14	0.30	5	4.26	16	5.29	g2
67	3	MT ensemble	HSQGTFTSDYDKYLDSRRAQIFQQWLEAEE	-11.18	-8.99	-11.22	-9.05	-10.75	-9.62	-12.72	-9.33	-10.59	-9.77	-11.29	0.84	-9.35	0.34	7	4.73	17	5.01	g2
68	3	MT ensemble	HSQGTFTSDYDKYLDSRRAHDQVQWLEAEE	-11.17	-9.00	-11.12	-9.17	-10.61	-9.64	-12.57	-9.79	-10.60	-9.83	-11.21	0.80	-9.49	0.38	7	5.34	18	5.02	g2
69	3	MT ensemble	HSQGTFTSDYDKYLDSRRAQCFQQWLEAEE	-11.17	-8.94	-11.21	-9.05	-10.81	-9.63	-12.91	-9.38	-10.63	-9.81	-11.35	0.91	-9.36	0.37	7	5.34	17	5.02	g2
70	3	MT ensemble	HSQGTFTSDYDKYLDSRRAQTFRQWLEAEE	-11.16	-8.87	-11.22	-9.05	-10.75	-9.62	-12.72	-9.33	-10.59	-9.77	-11.29	0.84	-9.33	0.37	7	5.41	17	5.29	g2
71	1	MT ensemble	HAEGTFTSDVASYLEGQAAKEFIPWLVKGR	-7.49	-11.21	-7.58	-11.18	-7.84	-11.01	-7.61	-10.92	-7.74	-9.42	-7.65	0.14	-10.75	0.75	17	5.38	2	2.27	g3
72	3	MT ensemble	YSQGTFTSDYSAYLEEEAVRFFINWLLAG-	-8.95	-12.10	-10.28	-11.73	-9.82	-10.64	-9.85	-13.28	-9.25	-11.44	-9.63	0.53	-11.84	0.97	14	1.86	13	-0.14	g3
73	3	MT ensemble	YSQGTFTSDYSAYLEEEAVRDFITWLLAG-	-8.94	-12.07	-10.26	-11.73	-9.90	-10.70	-9.73	-13.13	-9.34	-11.43	-9.63	0.51	-11.81	0.89	13	2.68	13	0.27	g3
74	3	MT ensemble	YSQGTFTSDYSAYLEEEAVRNFIWWLLAG-	-8.90	-12.05	-10.28	-11.74	-9.90	-10.62	-9.98	-13.25	-9.33	-11.46	-9.68	0.55	-11.82	0.96	14	2.08	13	-0.15	g3
75	3	MT ensemble	YSQGTFTSDYSAYLEEEAVRDFIDWLLAG-	-8.85	-12.02	-10.26	-11.73	-9.90	-10.70	-9.71	-13.09	-9.34	-11.43	-9.61	0.54	-11.79	0.88	13	2.32	13	0.76	g3

Supplementary Table 7: Multi-task ensemble of neural networks. Totals after removing duplicates and overlaps with the training data.

	1st generation		2st generation		3rd generation
Desired activity profile	Total (after clearance) 69639	No. of selected seeds	Total (after clearance) 13902	No of selected seeds	Total (after clearance) 8382
Highly active at both receptors	5	5	396	5	755
Selectively active towards GLP-1R	4145	10	651	5	1490
Selectively active towards GCGR	138	10	1022	5	631
Sequences with no predicted activity against both receptors	23703	-	13	-	11

Supplementary Table 8: Random Forest. Totals after removing duplicates and overlaps with the training data.

	1st generation		2st generation		3rd generation
Desired activity profile	Total (after clearance) 69639	No. of selected seeds	Total (after clearance) 13902	No of selected seeds	Total (after clearance) 8382
Highly active at both receptors	309	5	1281	5	1141
Selectively active towards GLP-1R	4599	10	3895	5	2119
Selectively active towards GCGR	38	10	4060	5	2051
Sequences with no predicted activity against both receptors	18169	-	0	-	0

Supplementary Table 9: Ridge Regression. Totals after removing duplicates and overlaps with the training data.

	1st generation		2st generation		3rd generation
Desired activity profile	Total (after clearance) 69639	No. of selected seeds	Total (after clearance) 13902	No of selected seeds	Total (after clearance) 8382
Highly active at both receptors	254	5	1000	5	1364
Selectively active towards GLP-1R	3814	10	3517	5	2283
Selectively active towards GCGR	297	10	2574	5	1712
Sequences with no predicted activity against both receptors	20211	-	0	-	0

Supplementary Table 10: Gaussian Process Regression. Totals after removing duplicates and overlaps with the training data.*$\log_{10}$EC$_{50}$GLP-1R [M] = -11 and $\log_{10}$ EC$_{50}$GCGR [M] = -11, **$\log_{10}$ EC$_{50}$GCGR [M] < -11 and $\log_{10}$ EC$_{50}$ GLP1-R [M] > -10.

	1st generation		2st generation		3rd generation
Desired activity profile	Total (after clearance) 69639	No. of selected seeds	Total (after clearance) 13902	No of selected seeds	Total (after clearance) 8382
Highly active at both receptors	1/903*	1/4*	0/529*	5	23*
Selectively active towards GLP-1R	1595	10	92	5	50
Selectively active towards GCGR	0/640**	10**	0/1291**	5	50**
Sequences with no predicted activity against both receptors	16678	-	0	-	0

Supplementary Table 11: Support Vector Regression. Totals after removing duplicates and overlaps with the training data.*$\log_{10}$EC$_{50}$GLP-1R [M] = -11 and $\log_{10}$ EC$_{50}$GCGR [M] = -11, **$\log_{10}$ EC$_{50}$GCGR [M] < -11 and $\log_{10}$ EC$_{50}$ GLP1-R [M] > -10.

	1st generation		2st generation		3rd generation
Desired activity profile	Total (after clearance) 69639	No. of selected seeds	Total (after clearance) 13902	No of selected seeds	Total (after clearance) 8382
Highly active at both receptors	0/1921*	5*	0/756*	5	104*
Selectively active towards GLP-1R	1318	10	2918	5	517
Selectively active towards GCGR	0/288**	10 **	0/242**	5	98**
Sequences with no predicted activity against both receptors	16678	-	0	-	0

Supplementary Table 14: Performance comparison of different models at predicting the GCGR and GLP-1R binding affinity of the 15 model-designed peptides. These models use the same hyperparameters that were found using cross-validation, with accuracy metrics on held-out test sequences reported in Table 1 (main text) and Supplementary Table 1.

Models	RMSE		MAE		R^2	
	GCGR	GLP1-R	GCGR	GLP1-R	GCGR	GLP1-R
Ridge	3.331	2.047	2.279	1.833	-1.098	0.246
SVR	2.586	1.718	1.882	1.380	-0.265	0.469
GPR	2.323	1.989	1.66	1.631	-0.02	0.288
Random Forest	2.303	1.988	1.744	1.462	-0.003	0.289
NN single-task	2.209	1.72	1.631	1.466	0.077	0.468
NN multi-task	2.602	1.772	1.861	1.479	-0.28	0.435
NN multi-task ensemble	2.555	1.65	1.885	1.386	-0.235	0.51
