# Supplementary material for: Machine learning designs new GCGR/GLP-1R dual agonists with enhanced biological potency
Source: Nat Chem. 2024 May 16;16(9):1436–44. doi: 10.1038/s41557-024-01532-x (PMC11374683; doi:10.1038/s41557-024-01532-x)
Supplement: Supplementary file 7 — Peptide characterization, structures and analytical data. [file 41557_2024_1532_MOESM7_ESM.pdf]

Supporting Information: Predicted Peptide Sequence Characterisation

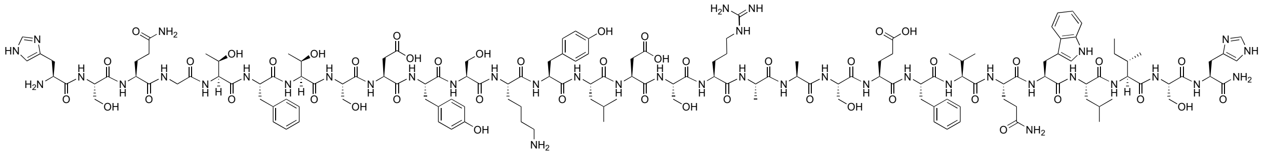

Peptide P1: Free-HSQGT^{5} FTSDY^{10} SKYLD^{15} SRAAS^{20} EFVQW^{25} LISH-amide Molecular Weight: 3360.60

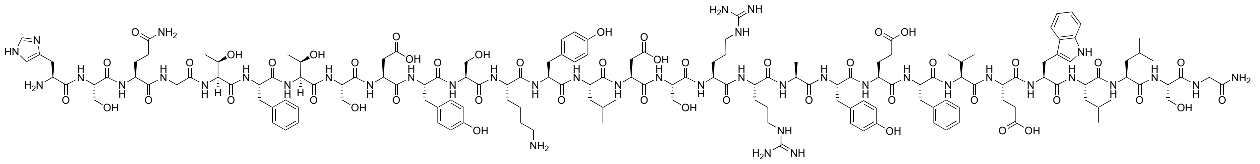

Peptide P2: Free-HSQGT^{5} FTSDY^{10} SKYLD^{15} SRRAY^{20} EFVEW^{25} LLSG-amide Molecular Weight: 3442.70

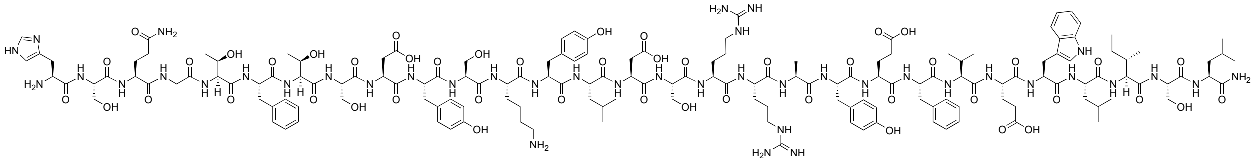

Peptide P3: Free-HSQGT^{5} FTSDY^{10} SKYLD^{15} SRRAY^{20} EFVEW^{25} LISL-amide Molecular Weight: 3498.81

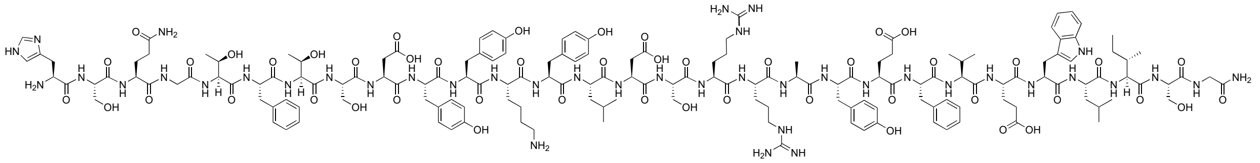

Peptide P4: Free-HSQGT^{5} FTSDY^{10} YKYLD^{15} SRRAY^{20} EFVEW^{25} LISG-amide Molecular Weight: 3518.80

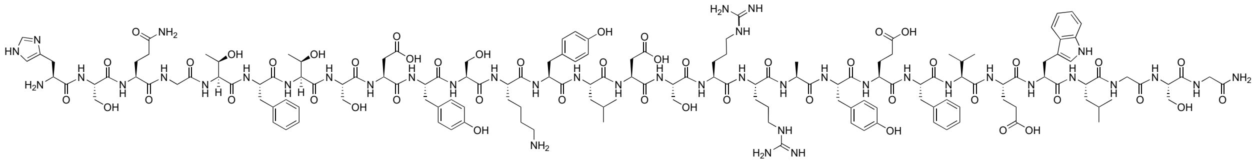

Peptide P5: Free-HSQGT^{5} FTSDY^{10} SKYLD^{15} SRRAY^{20} EFVEW^{25} LGSG-amide Molecular Weight: 3386.60

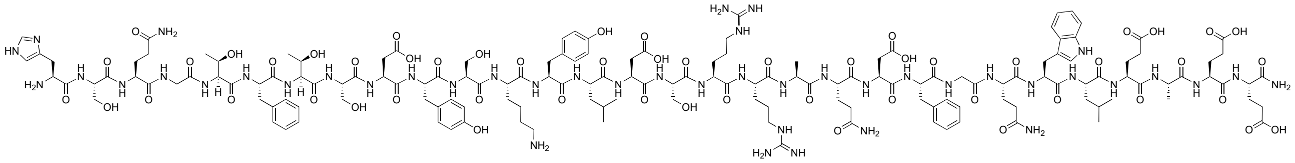

Peptide P6: Free-HSQGT^{5} FTSDY^{10} SKYLD^{15} SRRAQ^{20} DFGQW^{25} LEAEE^{30}-amide Molecular Weight: 3551.70

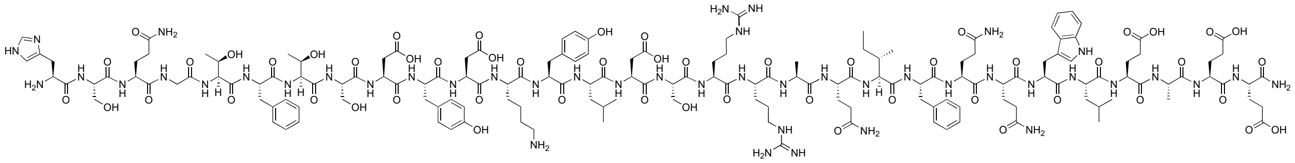

Peptide P7: Free-HSQGT^{5} FTSDY^{10} DKYLD^{15} SRRAQ^{20} IFQQW^{25} LEAEE^{30}-amide Molecular Weight: 3648.86

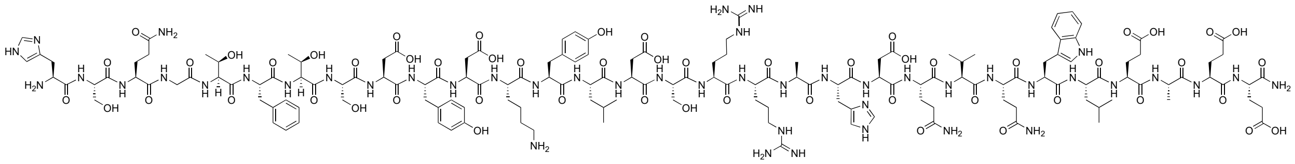

Peptide P8: Free-HSQGT^{5} FTSDY^{10} DKYLD^{15} SRRAH^{20} DQVQW^{25} LEAEE^{30}-amide Molecular Weight: 3611.76

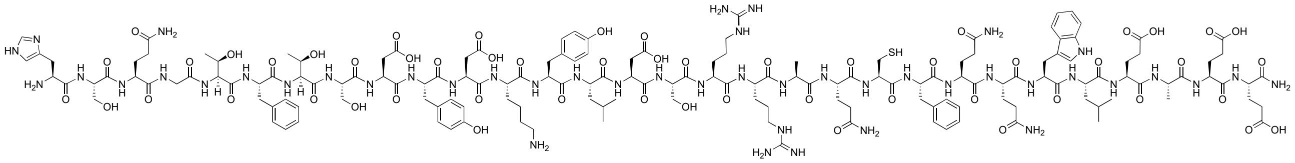

Peptide P9: Free-HSQGT^{5} FTSDY^{10} DKYLD^{15} SRRAQ^{20} CFQQW^{25} LEAEE^{30}-amide Molecular Weight: 3638.84

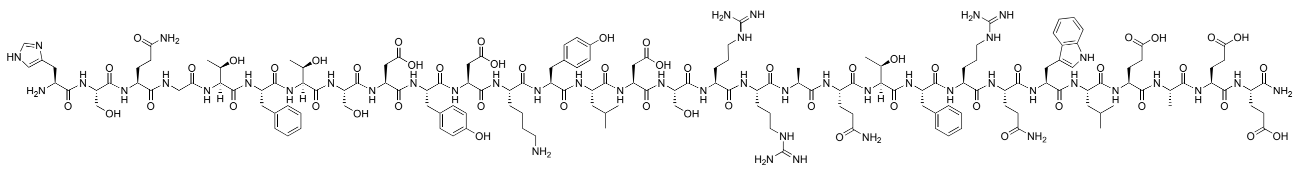

Peptide P10: Free-HSQGT[5] FTSDY[10] DKYLD[15] SRRAQ[20] TFRQW[25] LEAEE[30]-amide Molecular Weight: 3664.86

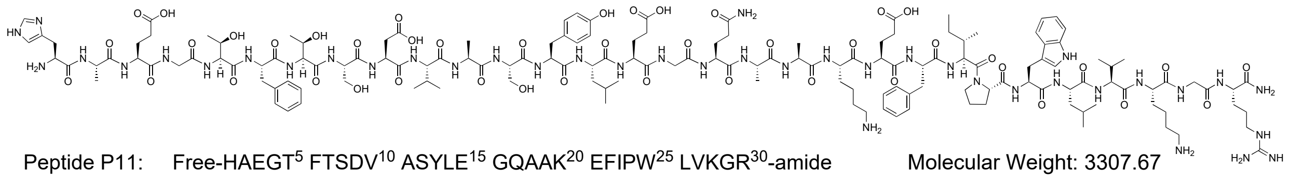

Peptide P11: Free-HAEGT[5] FTSDV[10] ASYLE[15] GQAAK[20] EFIPW[25] LVKGR[30]-amide Molecular Weight: 3307.67

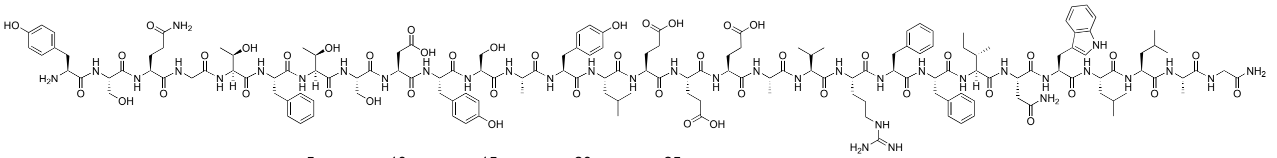

Peptide P12: Free-YSQGT[5] FTSDY[10] SAYLE[15] EEAVR[20] FFINW[25] LLAG-amide Molecular Weight: 3377.67

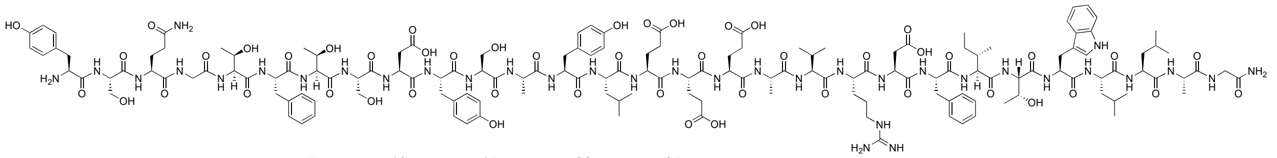

Peptide P13: Free-YSQGT[5] FTSDY[10] SAYLE[15] EEAVR[20] DFITW[25] LLAG-amide Molecular Weight: 3332.58

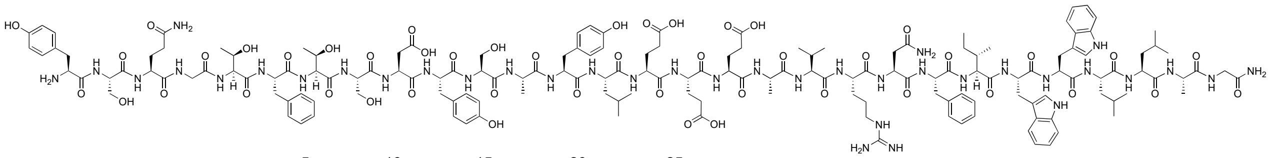

Peptide P14: Free-YSQGT[5] FTSDY[10] SAYLE[15] EEAVR[20] NFIWW[25] LLAG-amide Molecular Weight: 3416.70

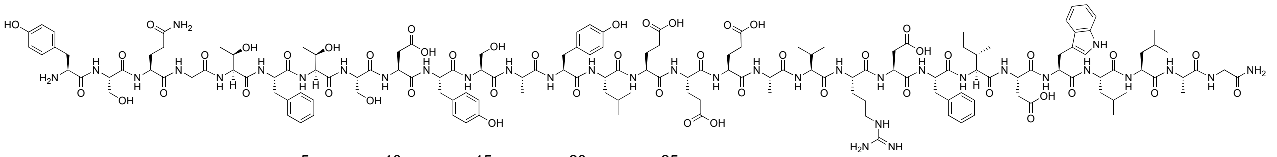

Peptide P15: Free-YSQGT[5] FTSDY[10] SAYLE[15] EEAVR[20] DFIDW[25] LLAG-amide Molecular Weight: 3346.56

Alias	Peptide Sequence	Purity % (AUC)	M.W. (Da)	m/z	$[M+2H]^{2+}$	$[M+3H]^{3+}$	$[M+4H]^{4+}$	$[M+5H]^{5+}$
P1	Free-HSQGTFTSDYSKYLDSRAASEFVQWLISH-amide	97.1	3360.60	Expected	1681.30	1121.20	841.15	673.12
				Observed	N/A	1120.21	840.82	673.03
P2	Free-HSQGTFTSDYSKYLDSRRAYEFVEWLLSG-amide	95.5	3442.70	Expected	1722.35	1148.57	861.68	689.54
				Observed	N/A	1148.32	861.64	689.37
P3	Free-HSQGTFTSDYSKYLDSRRAYEFVEWLISL-amide	96.1	3498.81	Expected	1750.41	1167.27	875.70	700.76
				Observed	N/A	1167.16	875.60	700.61
P4	Free-HSQGTFTSDYYKYLDSRRAYEFVEWLISG-amide	98.6	3518.80	Expected	1760.40	1173.93	880.70	704.76
				Observed	N/A	1173.43	880.23	704.55
P5	Free-HSQGTFTSDYSKYLDSRRAYEFVEWLGSG-amide	97.1	3386.60	Expected	1694.30	1129.87	847.65	678.32
				Observed	N/A	1129.60	847.65	678.15
P6	Free-HSQGTFTSDYSKYLDSRRAQDFGQWLEAEE-amide	100.0	3551.70	Expected	1776.85	1184.90	888.93	711.34
				Observed	N/A	1184.39	888.90	711.21
P7	Free-HSQGTFTSDYDKYLDSRRAQIFQQWLEAEE-amide	96.2	3648.86	Expected	1825.43	1217.29	913.22	730.77
				Observed	N/A	1216.05	912.87	730.39
P8	Free-HSQGTFTSDYDKYLDSRRAHDQVQWLEAEE-amide	100.0	3611.76	Expected	1806.88	1204.92	903.94	723.35
				Observed	N/A	1204.45	903.52	723.17
P9	Free-HSQGTFTSDYDKYLDSRRAQCFQQWLEAEE-amide	100.0	3638.84	Expected	1820.42	1213.95	910.71	728.77
				Observed	N/A	1213.45	910.94	728.79
P10	Free-HSQGTFTSDYDKYLDSRRAQTFRQWLEAEE-amide	97.5	3664.86	Expected	1833.43	1222.62	917.22	733.97
				Observed	N/A	1222.76	916.80	733.94
P11	Free-HAEGTFTSDVASYLEGQAAKEFIPWLVKGR-amide	95.1	3307.67	Expected	1654.84	1103.56	827.92	662.53
				Observed	N/A	1103.67	827.90	622.46
P12	Free-YSQGTFTSDYSAYLEEEAVRFFINWLLAG-amide	97.1	3377.67	Expected	1689.84	1126.89	845.42	676.53
				Observed	1689.02	1126.58	845.01	N/A
P13	Free-YSQGTFTSDYSAYLEEEAVRDFITWLLAG-amide	97.3	3332.58	Expected	1667.29	1111.86	834.15	667.52
				Observed	1666.87	1111.87	833.75	N/A
P14	Free-YSQGTFTSDYSAYLEEEAVRNFIWWLLAG-amide	96.0	3416.70	Expected	1709.35	1139.90	855.18	684.34
				Observed	1709.02	1139.69	854.56	N/A
P15	Free-YSQGTFTSDYSAYLEEEAVRNFIWWLLAG-amide	95.0	3346.56	Expected	1674.28	1116.52	837.64	670.31
				Observed	1674.37	1116.57	837.30	N/A

HPLC Analysis Report

Data File name:C:\CHEM32\1\DATA\CHECKOUT\02JULY20A 2020-07-02 08-05-44\ ->
Method name: C:\CHEM32\1\DATA\CHECKOUT\02JULY20A 2020-07-02 08-05-44\ ->
Injection date:: 02/07/2020
Sample Name:41450 FINAL

P1 Free-HSQGTFTSDYSKYLDSRAASEFVQWLISH-amide

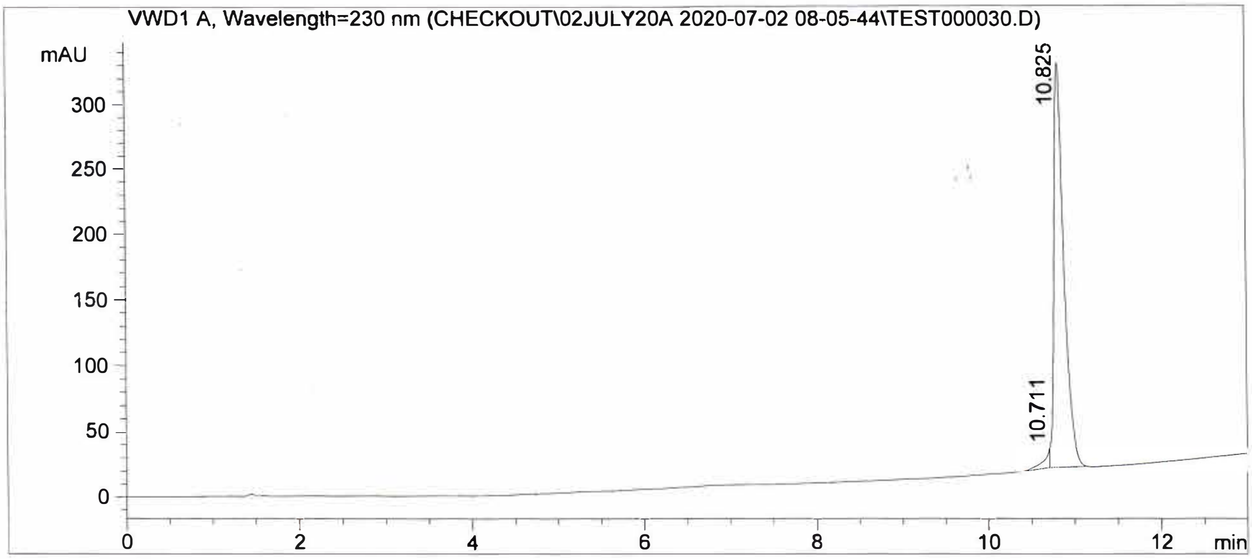

Column: ACE 3 C18-300 300A 150x2.1mm
Buffer A: 0.1 % TFA in H2O
Buffer B: 0.1 % TFA in MeCN
Flow rate: 0.35mL/min
Gradient: 2 to 70% B over 13 min

#	RT (Min)	Area	Height	Area %
1	10.711	71.531	14.254	2.8
2	10.825	2447.027	308.735	97.1

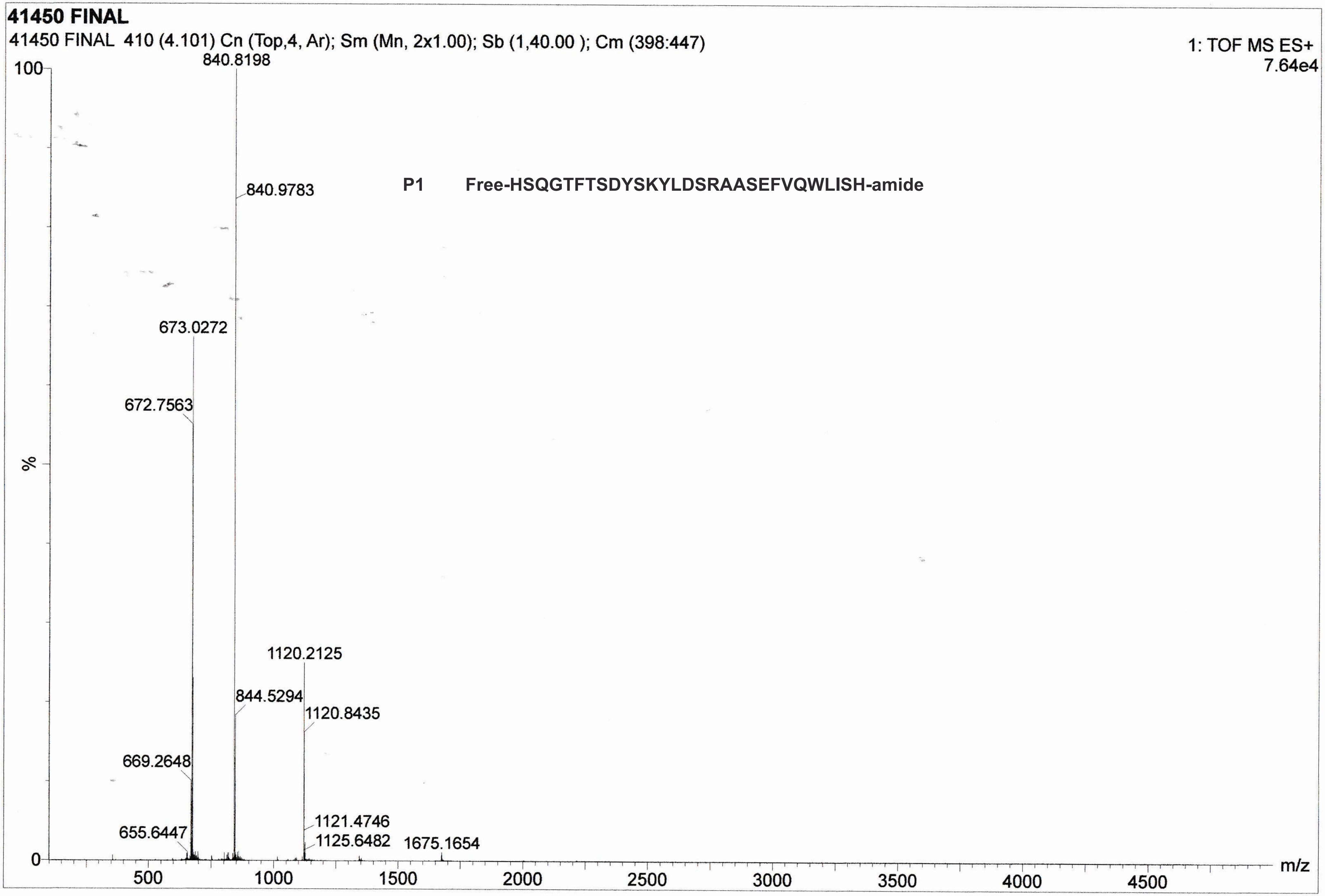
41450 FINAL
41450 FINAL 410 (4.101) Cn (Top,4, Ar); Sm (Mn, 2x1.00); Sb (1,40.00); Cm (398:447)
1: TOF MS ES+
7.64e4
P1 Free-HSQGTFTSDYSKYLDSRAASEFVQWLISH-amide
840.8198
840.9783
673.0272
672.7563
1120.2125
844.5294
1120.8435
669.2648
655.6447
1121.4746
1125.6482
1675.1654
100
%
0
500
1000
1500
2000
2500
3000
3500
4000
4500
m/z

HPLC Analysis Report

Data File name:C:\CHEM32\1\DATA\CHECKOUT\06JUL20A 2020-07-06 09-22-20\ ->
Method name: C:\CHEM32\1\DATA\CHECKOUT\06JUL20A 2020-07-06 09-22-20\ ->
Injection date:: 06/07/2020
Sample Name:41451 FINAL

P2 Free-HSQGTFTSDYSKYLDSRRAYEFVEWLLSG-amide

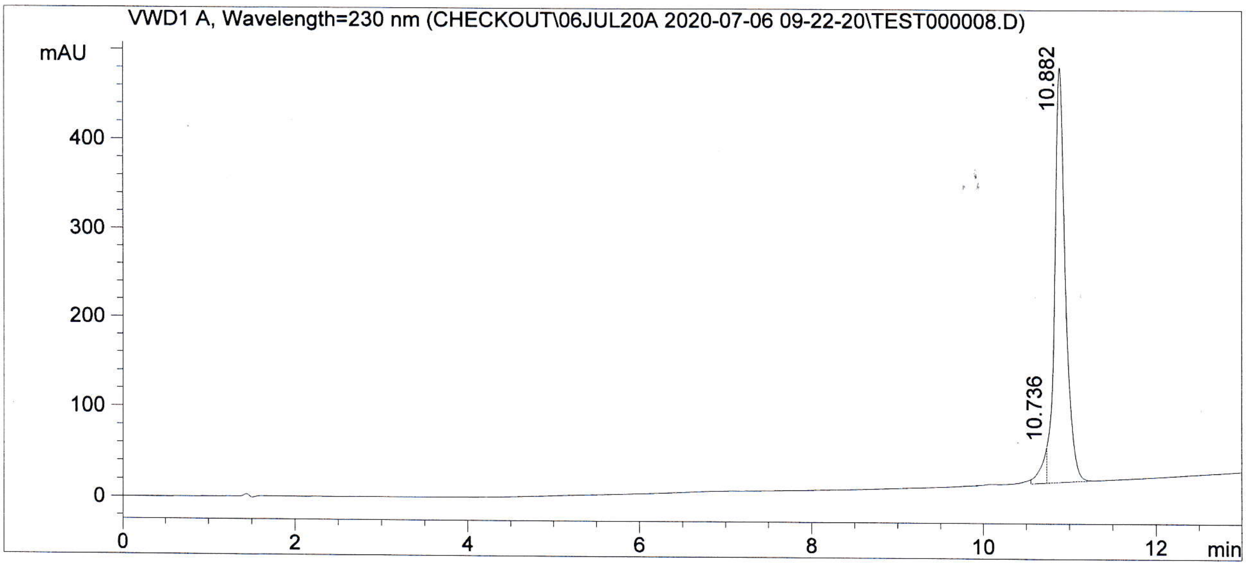

Column: ACE 3 C18-300 300A 150x2.1mm
Buffer A: 0.1 % TFA in H2O
Buffer B: 0.1 % TFA in MeCN
Flow rate: 0.35mL/min
Gradient: 2 to 70% B over 13 min

#	RT (Min)	Area	Height	Area %
1	10.736	186.735	39.180	4.4
2	10.882	3972.303	462.410	95.5

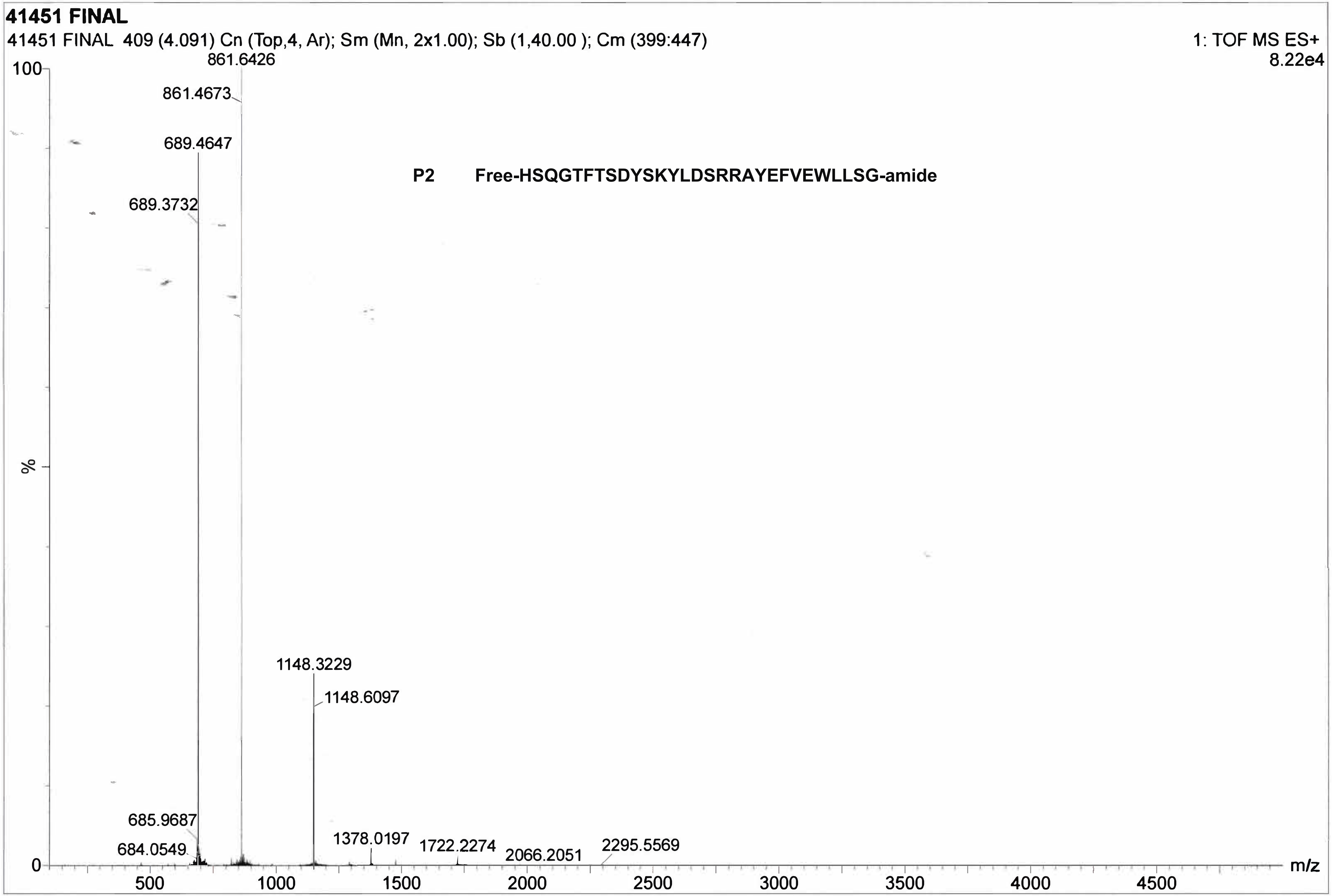
41451 FINAL
41451 FINAL 409 (4.091) Cn (Top,4, Ar); Sm (Mn, 2x1.00); Sb (1,40.00); Cm (399:447)
1: TOF MS ES+
8.22e4
P2 Free-HSQGTFTSDYSKYLDSRRAYEFVEWLLSG-amide
861.6426
861.4673
689.4647
689.3732
1148.3229
1148.6097
685.9687
684.0549
1378.0197
1722.2274
2066.2051
2295.5569
100
%
0
500
1000
1500
2000
2500
3000
3500
4000
4500
m/z

HPLC Analysis Report

Data File name:C:\CHEM32\1\DATA\CHECKOUT\09JULY20B 2020-07-09 14-24-49\ ->
Method name: C:\CHEM32\1\DATA\CHECKOUT\09JULY20B 2020-07-09 14-24-49\ ->
Injection date:: 09/07/2020
Sample Name:41452 FINAL

P3 Free-HSQGTFTSDYSKYLDSRRAYEFVEWLISL-amide

crb CAMBRIDGE RESEARCH BIOCHEMICALS

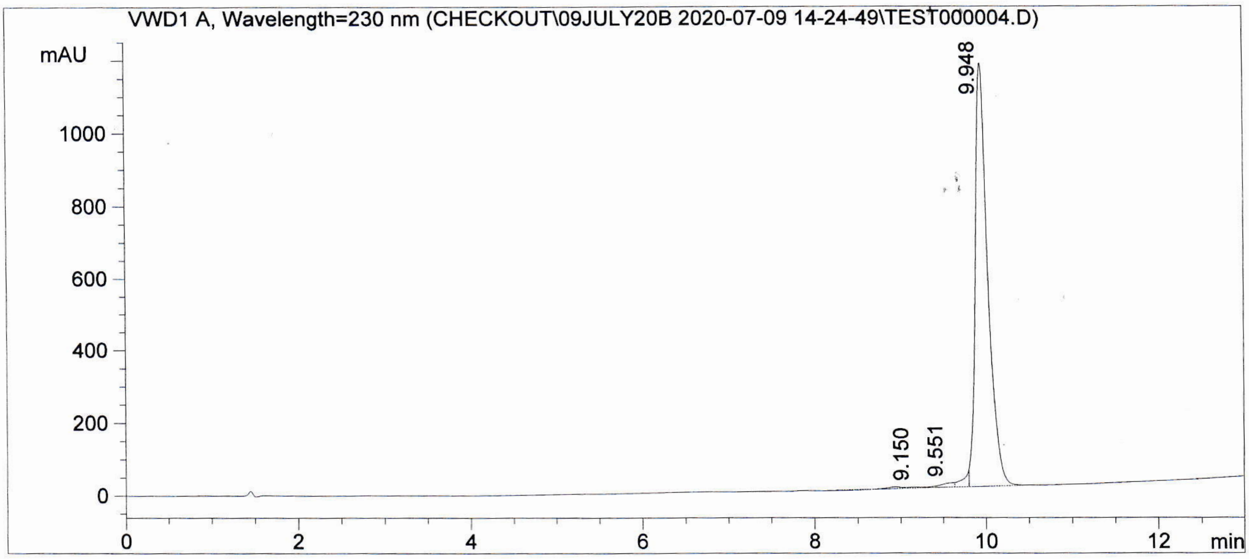

Column: ACE 3 C18-300 300A 150x2.1mm
Buffer A: 0.1 % TFA in H2O
Buffer B: 0.1 % TFA in MeCN
Flow rate: 0.35mL/min
Gradient: 2 to 70% B over 13 min

#	RT (Min)	Area	Height	Area %
1	8.937	55.248	4.852	0.4
2	9.150	27.869	3.457	0.2
3	9.551	153.766	11.545	1.2
4	9.799	230.530	47.292	1.8
5	9.948	11738.492	1166.658	96.1

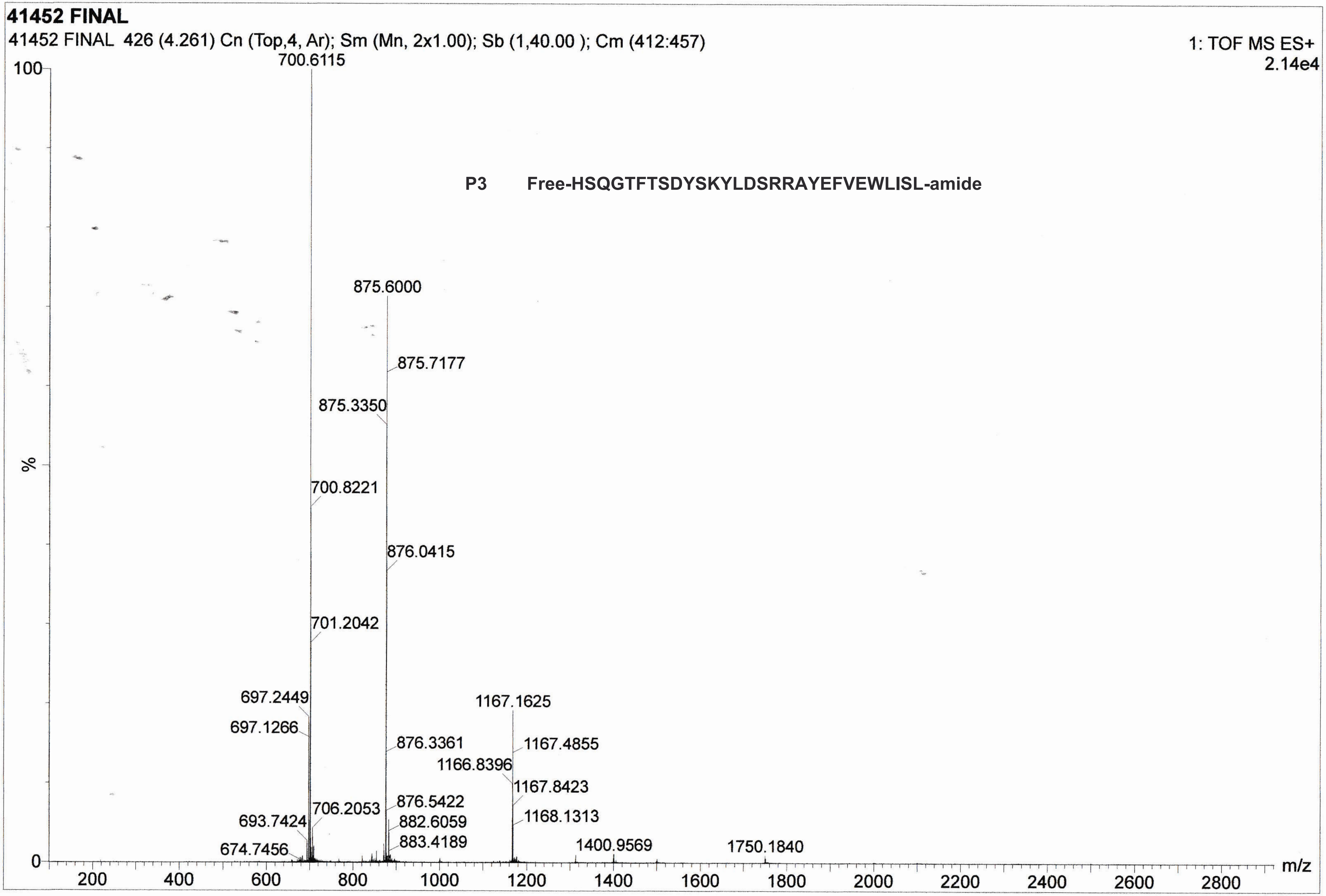

41452 FINAL
41452 FINAL 426 (4.261) Cn (Top,4, Ar); Sm (Mn, 2x1.00); Sb (1,40.00); Cm (412:457)
1: TOF MS ES+
2.14e4
P3 Free-HSQGTFTSDYSKYLDSRRAYEFVEWLISL-amide
700.6115
875.6000
875.7177
875.3350
700.8221
876.0415
701.2042
697.2449
697.1266
1167.1625
876.3361
1167.4855
1166.8396
1167.8423
876.5422
706.2053
1168.1313
693.7424
882.6059
883.4189
674.7456
1400.9569
1750.1840
%
100
0
m/z
200
400
600
800
1000
1200
1400
1600
1800
2000
2200
2400
2600
2800

HPLC Analysis Report

Data File name:C:\CHEM32\1\DATA\CHECKOUT\09JULY20B 2020-07-09 14-24-49\ ->
Method name: C:\Chem32\1\DATA\CHECKOUT\09JULY20B 2020-07-09 14-24-49\CUST->
Injection date:: 09/07/2020
Sample Name:41453 FINAL

P4 Free-HSQGTFTSDYYKYLDSRRAYEFVEWLISG-amide

CAMBRIDGE RESEARCH BIOCHEMICALS

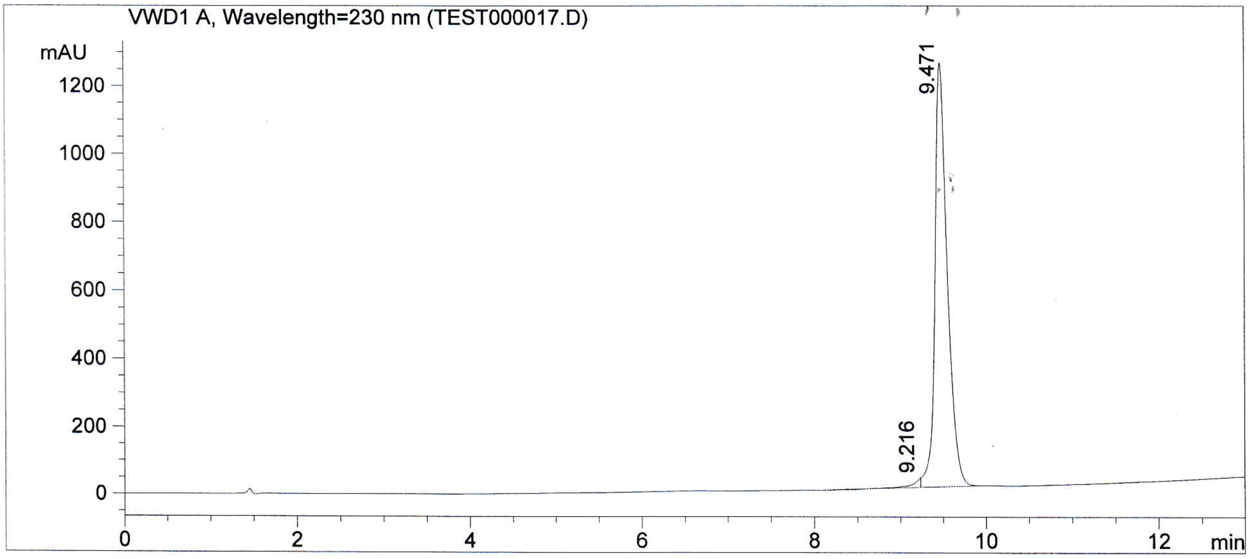

Column: ACE 3 C18-300 300A 150x2.1mm
Buffer A: 0.1 % TFA in H2O
Buffer B: 0.1 % TFA in MeCN
Flow rate: 0.35mL/min
Gradient: 2 to 70% B over 13 min

#	RT (Min)	Area	Height	Area %
1	9.216	172.575	24.598	1.3
2	9.471	12489.522	1246.128	98.6

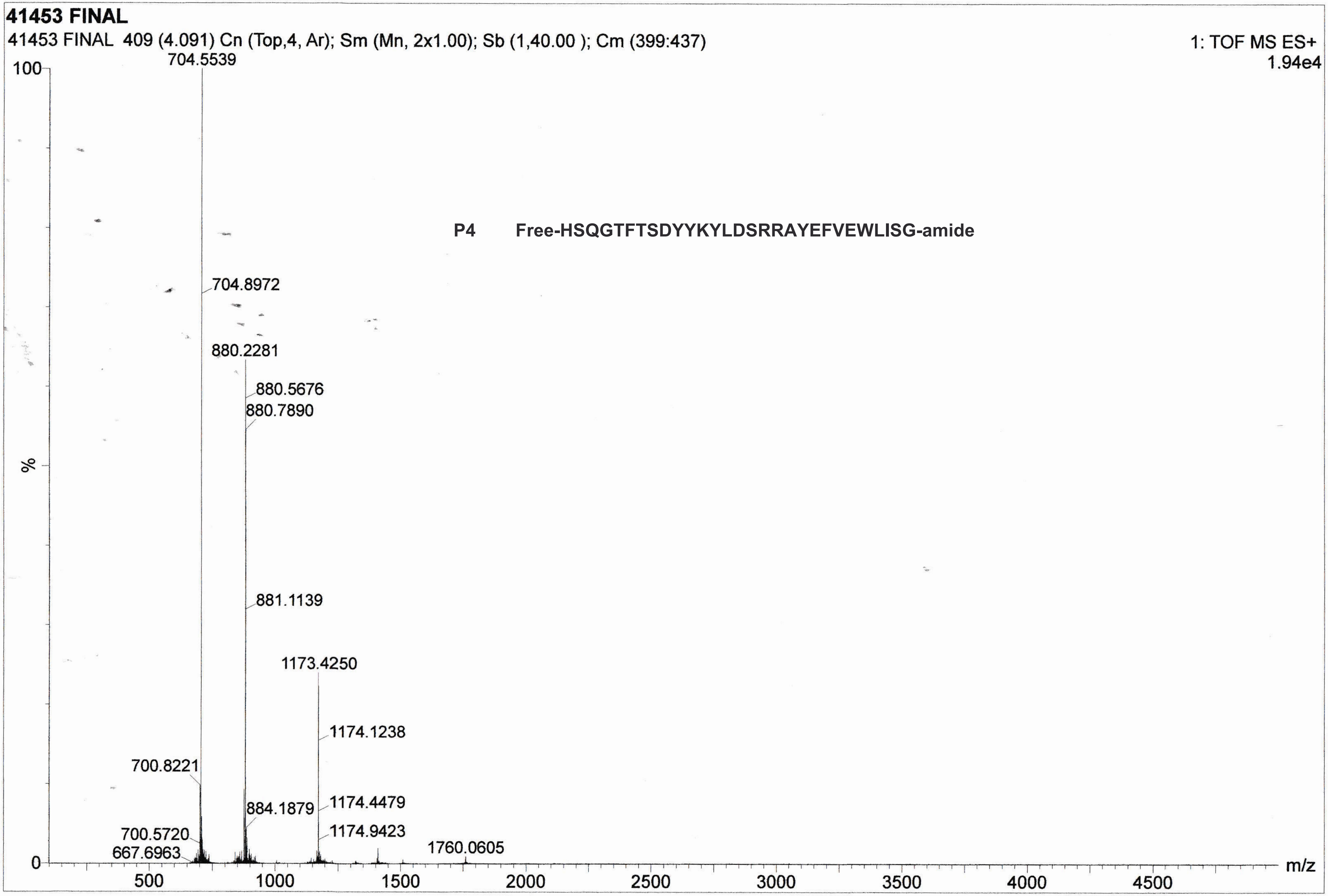
41453 FINAL
41453 FINAL 409 (4.091) Cn (Top,4, Ar); Sm (Mn, 2x1.00); Sb (1,40.00); Cm (399:437)
1: TOF MS ES+
1.94e4
P4 Free-HSQGTFTSDYYKYLDSRRAYEFVEWLISG-amide
704.5539
704.8972
880.2281
880.5676
880.7890
881.1139
1173.4250
1174.1238
700.8221
884.1879
1174.4479
700.5720
1174.9423
667.6963
1760.0605
%
100
0
500
1000
1500
2000
2500
3000
3500
4000
4500
m/z

HPLC Analysis Report

Data File name:C:\CHEM32\1\DATA\CHECKOUT\06JUL20A 2020-07-06 09-22-20\ ->
Method name: C:\CHEM32\1\DATA\CHECKOUT\06JUL20A 2020-07-06 09-22-20\ ->
Injection date:: 06/07/2020
Sample Name:41454 FINAL

P5 Free-HSQGTFTSDYSKYLDSRRAYEFVEWLGSG-amide

CAMBRIDGE RESEARCH BIOCHEMICALS

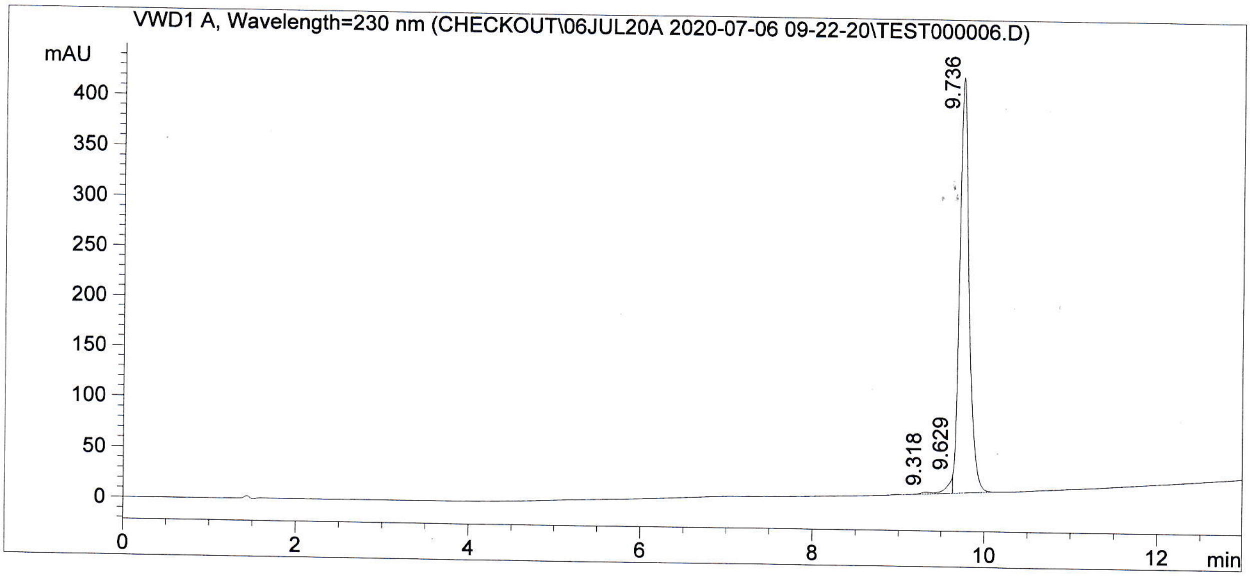

Column: ACE 3 C18-300 300A 150x2.1mm
Buffer A: 0.1 % TFA in H2O
Buffer B: 0.1 % TFA in MeCN
Flow rate: 0.35mL/min
Gradient: 2 to 70% B over 13 min

#	RT (Min)	Area	Height	Area %
1	9.318	20.639	2.023	0.6
2	9.629	72.050	16.605	2.2
3	9.736	3137.642	412.129	97.1

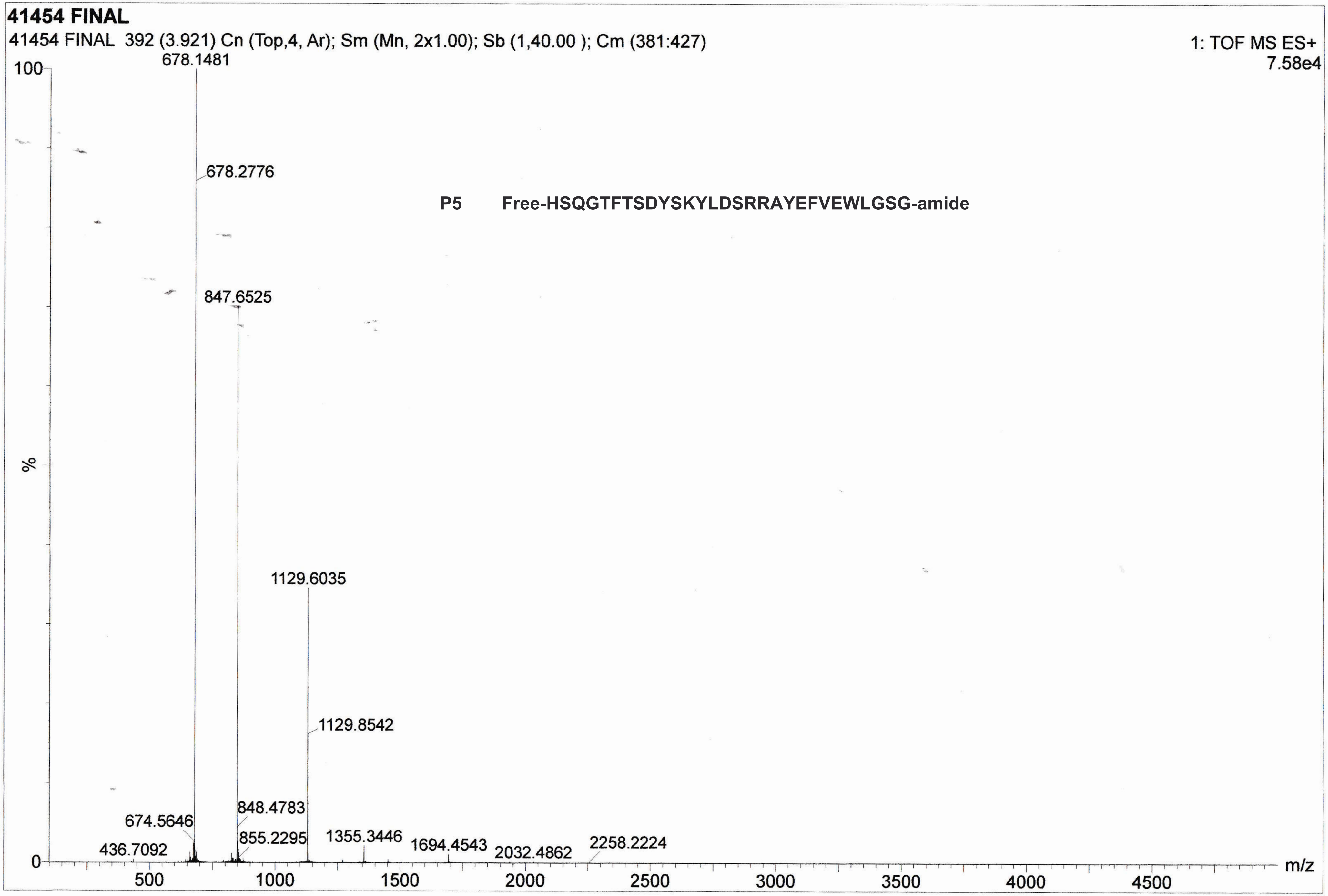
41454 FINAL
41454 FINAL 392 (3.921) Cn (Top,4, Ar); Sm (Mn, 2x1.00); Sb (1,40.00); Cm (381:427)
1: TOF MS ES+
7.58e4
P5 Free-HSQGTFTSDYSKYLDSRRAYEFVEWLGSG-amide
678.1481
678.2776
847.6525
1129.6035
1129.8542
848.4783
674.5646
855.2295
1355.3446
1694.4543
2032.4862
2258.2224
436.7092
100
%
0
500
1000
1500
2000
2500
3000
3500
4000
4500
m/z

HPLC Analysis Report

Data File name:C:\CHEM32\1\DATA\CHECKOUT\03JULY20A 2020-07-03 07-39-04\ ->
Method name: C:\Chem32\1\DATA\CHECKOUT\03JULY20A 2020-07-03 07-39-04\2-70->
Injection date:: 03/07/2020
Sample Name:41455 FINAL

P6 Free-HSQGTFTSDYSKYLDSRRAQDFGQWLEAEE-amide

crb CAMBRIDGE RESEARCH BIOCHEMICALS

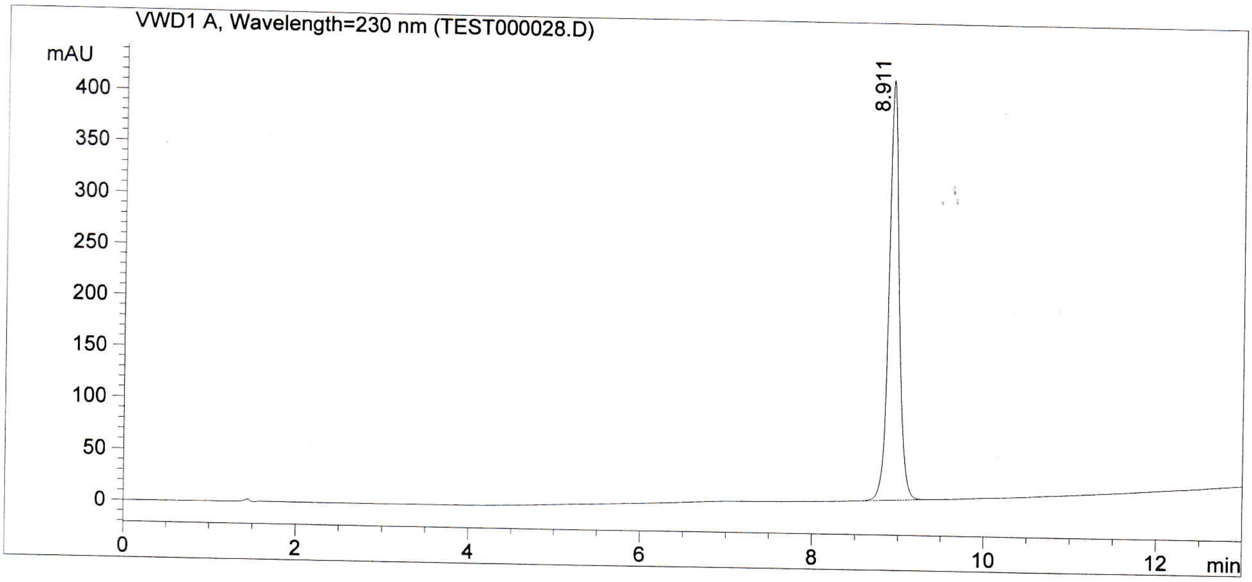

Column: ACE 3 C18-300 300A 150x2.1mm
Buffer A: 0.1 % TFA in H2O
Buffer B: 0.1 % TFA in MeCN
Flow rate: 0.35mL/min
Gradient: 2 to 70% B over 13 min

#	RT (Min)	Area	Height	Area %
1	8.911	3381.982	409.214	100.0

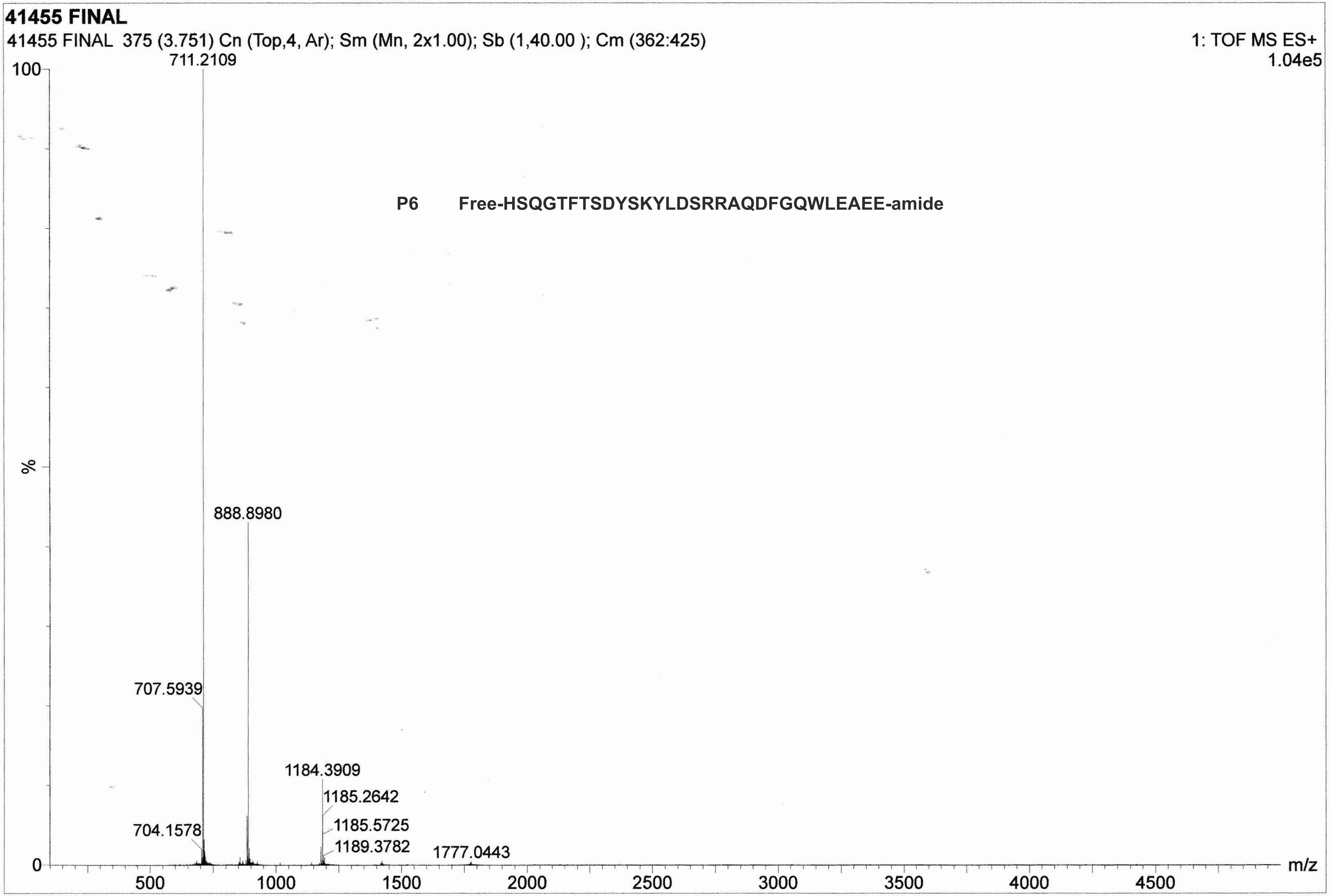
41455 FINAL
41455 FINAL 375 (3.751) Cn (Top,4, Ar); Sm (Mn, 2x1.00); Sb (1,40.00); Cm (362:425)
1: TOF MS ES+
1.04e5
P6 Free-HSQGTFTSDYSKYLDSRRAQDFGQWLEAEE-amide
711.2109
888.8980
707.5939
1184.3909
1185.2642
1185.5725
1189.3782
704.1578
1777.0443
100
%
0
500
1000
1500
2000
2500
3000
3500
4000
4500
m/z

HPLC Analysis Report

Data File name:C:\CHEM32\1\DATA\CHECKOUT\30JUNE20A 2020-06-30 07-03-22\ ->
Method name: C:\CHEM32\1\DATA\CHECKOUT\30JUNE20A 2020-06-30 07-03-22\ ->
Injection date:: 30/06/2020
Sample Name:41456 FINAL

P7 Free-HSQGTFTSDYDKYLDSRRAQIFQQWLEAEE-amide

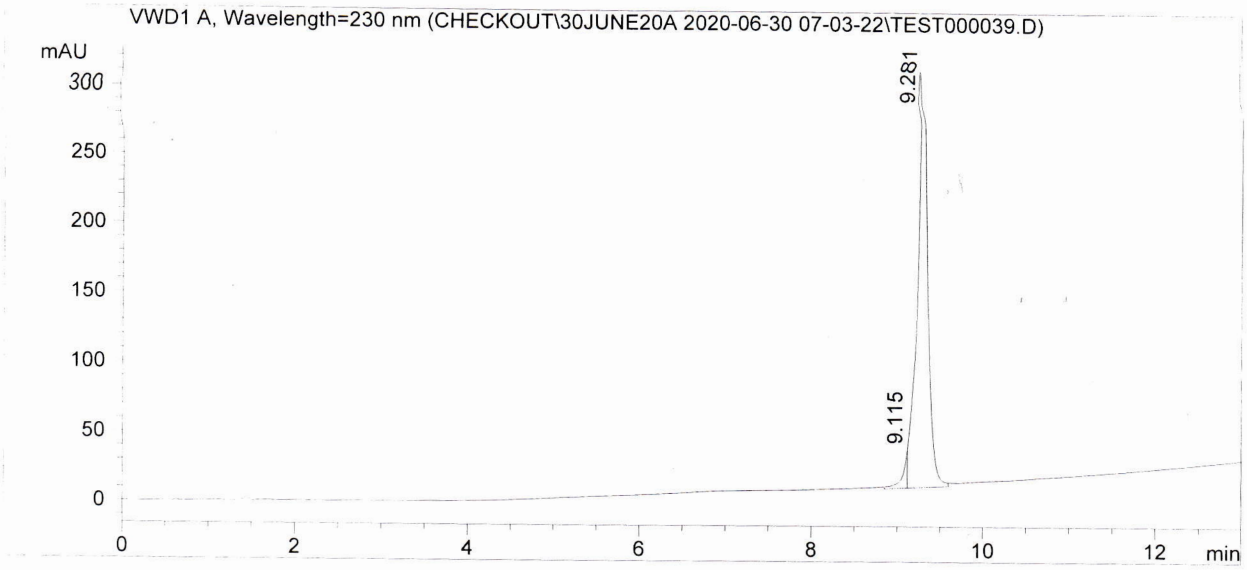

Column: ACE 3 C18-300 300A 150x2.1mm
Buffer A: 0.1 % TFA in H2O
Buffer B: 0.1 % TFA in MeCN
Flow rate: 0.35mL/min
Gradient: 2 to 70% B over 13 min

#	RT (Min)	Area	Height	Area %
1	9.115	100.097	27.492	3.7
2	9.281	2575.937	301.212	96.2

41456 FINAL

41456 FINAL 389 (3.891) Cn (Top,4, Ar); Sm (Mn, 2x1.00); Sb (1,40.00); Cm (375:446) 1: TOF MS ES+ 2.72e4

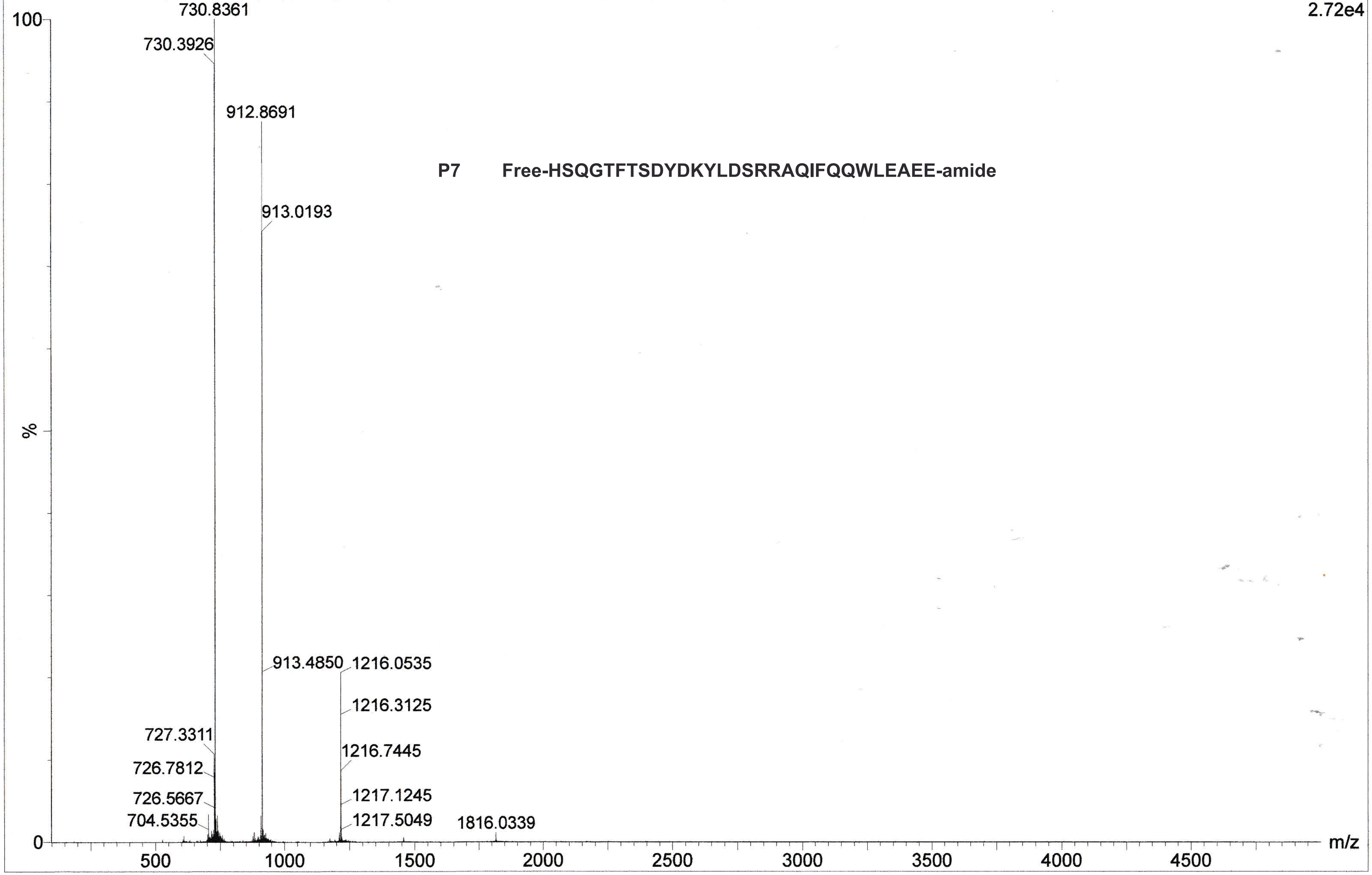

P7 Free-HSQGTFTSDYDKYLDSRRAQIFQQWLEAEE-amide

HPLC Analysis Report

Data File name:C:\CHEM32\1\DATA\CHECKOUT\03JULY20A 2020-07-03 07-39-04\ ->
Method name: C:\Chem32\1\DATA\CHECKOUT\03JULY20A 2020-07-03 07-39-04\2-70->
Injection date:: 03/07/2020
Sample Name:41457 FINAL

P8 Free-HSQGTFTSDYDKYLDSRRAHDQVQWLEAEE-amide

crb CAMBRIDGE RESEARCH BIOCHEMICALS

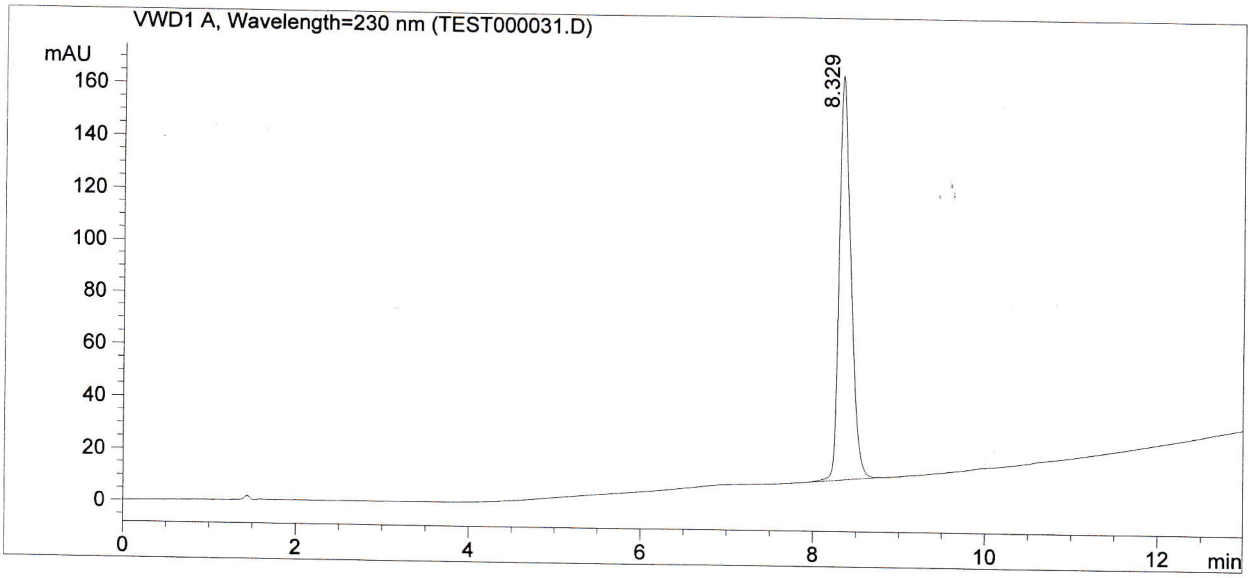

Column: ACE 3 C18-300 300A 150x2.1mm
Buffer A: 0.1 % TFA in H2O
Buffer B: 0.1 % TFA in MeCN
Flow rate: 0.35mL/min
Gradient: 2 to 70% B over 13 min

#	RT (Min)	Area	Height	Area %
1	8.329	1458.547	154.449	100.0

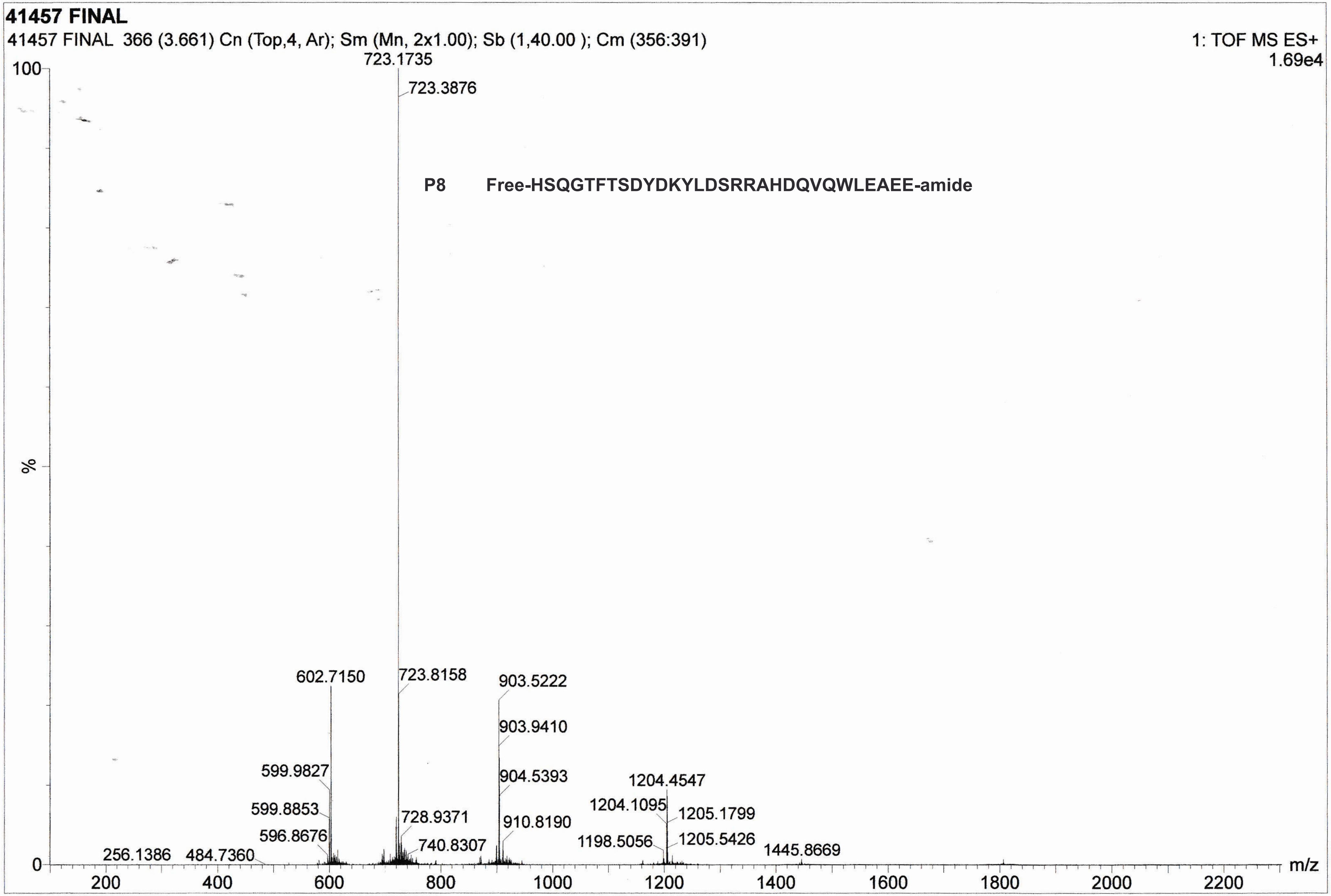
41457 FINAL
41457 FINAL 366 (3.661) Cn (Top,4, Ar); Sm (Mn, 2x1.00); Sb (1,40.00); Cm (356:391)
1: TOF MS ES+
1.69e4
P8 Free-HSQGTFTSDYDKYLDSRRAHDQVQWLEAEE-amide
723.1735
723.3876
602.7150
723.8158
903.5222
903.9410
599.9827
904.5393
1204.4547
599.8853
1204.1095
1205.1799
728.9371
910.8190
596.8676
740.8307
1198.5056
1205.5426
1445.8669
256.1386
484.7360
100
%
0
m/z
200
400
600
800
1000
1200
1400
1600
1800
2000
2200

HPLC Analysis Report

Data File name:C:\CHEM32\1\DATA\CHECKOUT\03JULY20A 2020-07-03 07-39-04\ ->
Method name: C:\Chem32\1\DATA\CHECKOUT\03JULY20A 2020-07-03 07-39-04\2-70->
Injection date:: 03/07/2020
Sample Name:41458 FINAL

P9 Free-HSQGTFTSDYDKYLDSRRAQCFQQWLEAEE-amide

CAMBRIDGE RESEARCH BIOCHEMICALS

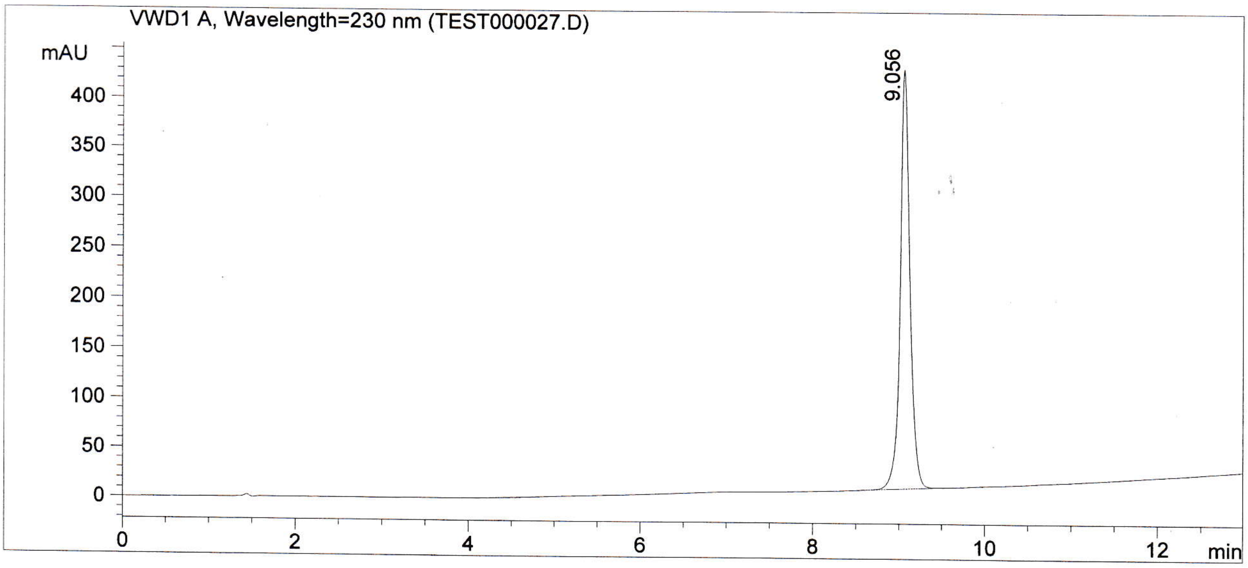

Column: ACE 3 C18-300 300A 150x2.1mm
Buffer A: 0.1 % TFA in H2O
Buffer B: 0.1 % TFA in MeCN
Flow rate: 0.35mL/min
Gradient: 2 to 70% B over 13 min

#	RT (Min)	Area	Height	Area %
1	9.056	3306.132	419.321	100.0

41458 FINAL

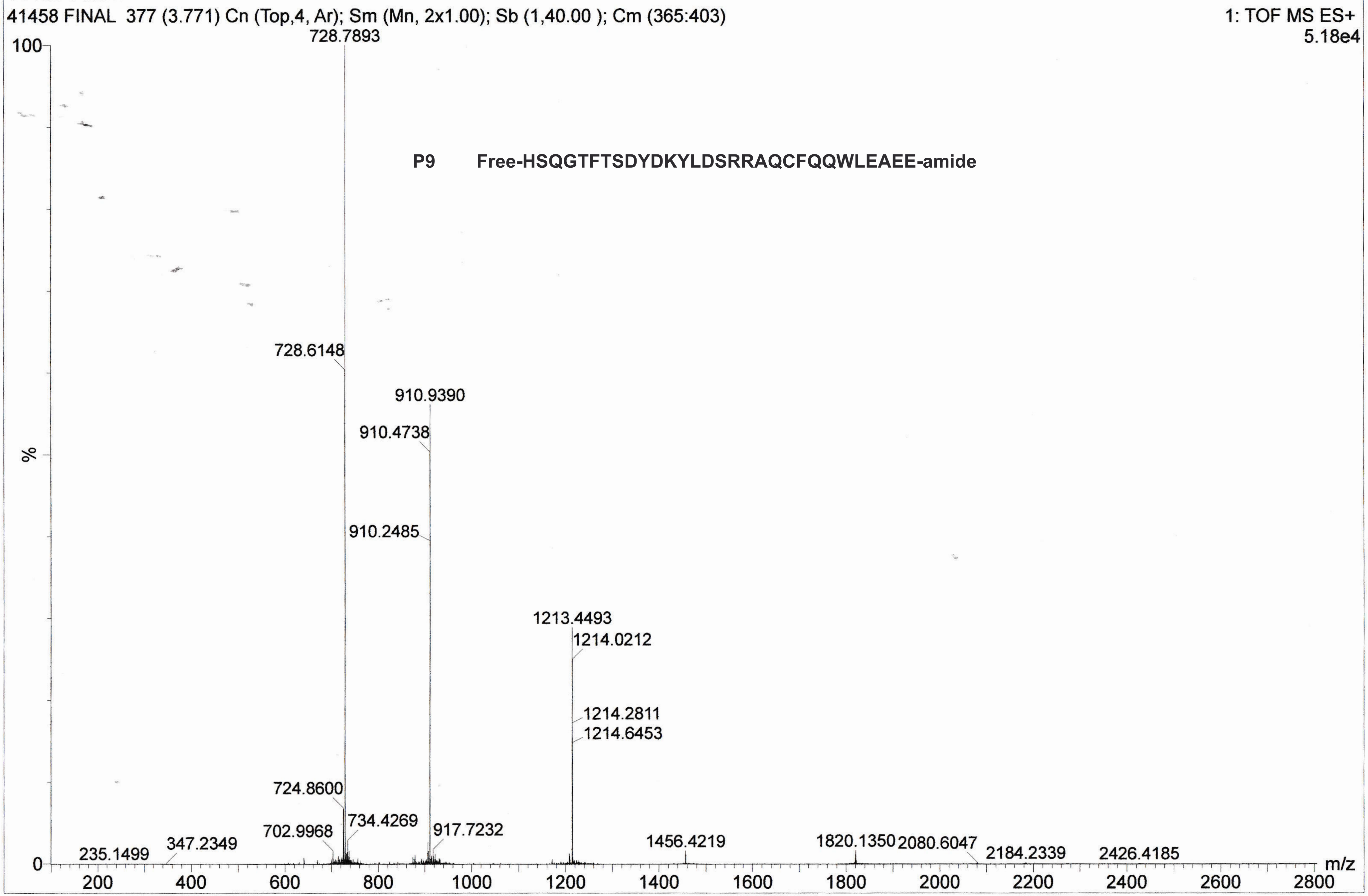
41458 FINAL 377 (3.771) Cn (Top,4, Ar); Sm (Mn, 2x1.00); Sb (1,40.00); Cm (365:403)
1: TOF MS ES+
5.18e4
P9 Free-HSQGTFTSDYDKYLDSRRAQCFQQWLEAEE-amide
728.7893
728.6148
910.9390
910.4738
910.2485
1213.4493
1214.0212
1214.2811
1214.6453
724.8600
734.4269
702.9968
917.7232
235.1499
347.2349
1456.4219
1820.1350
2080.6047
2184.2339
2426.4185
%
m/z
100
0
200
400
600
800
1000
1200
1400
1600
1800
2000
2200
2400
2600
2800

HPLC Analysis Report

Data File name:C:\CHEM32\1\DATA\CHECKOUT\06JUL20B 2020-07-06 14-16-37\ ->
Method name: C:\CHEM32\1\DATA\CHECKOUT\06JUL20B 2020-07-06 14-16-37\ ->
Injection date:: 06/07/2020
Sample Name:41459 FINAL

P10 Free-HSQGTFTSDYDKYLDSRRAQTFRQWLEAEE-amide

CAMBRIDGE RESEARCH BIOCHEMICALS

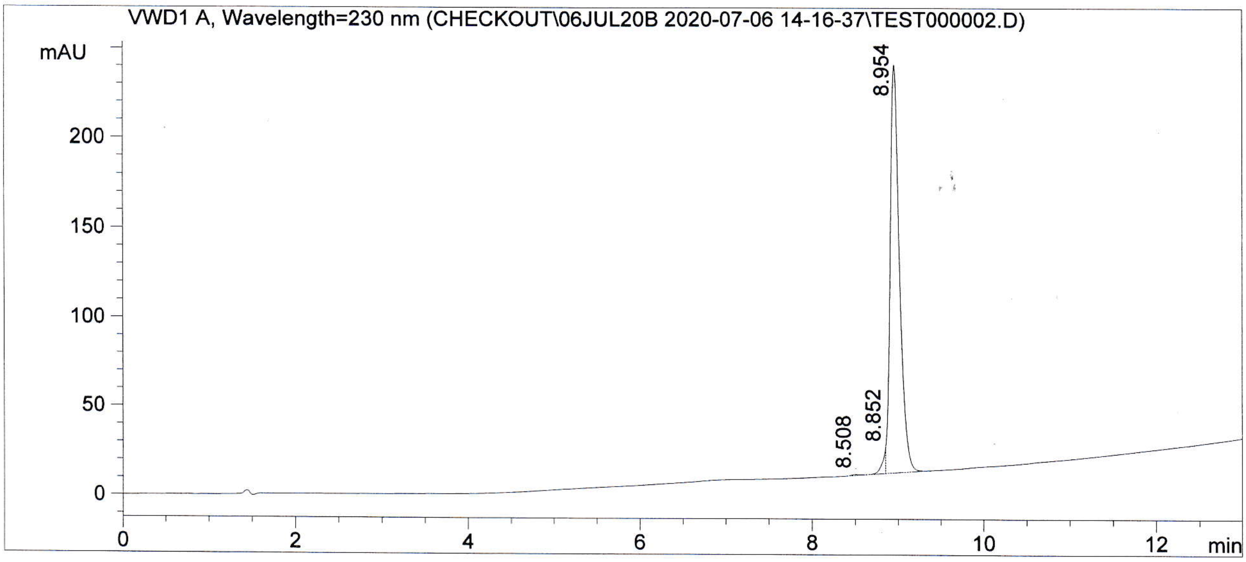

Column: ACE 3 C18-300 300A 150x2.1mm
Buffer A: 0.1 % TFA in H2O
Buffer B: 0.1 % TFA in MeCN
Flow rate: 0.35mL/min
Gradient: 2 to 70% B over 13 min

#	RT (Min)	Area	Height	Area %
1	8.508	4.649	0.567	0.2
2	8.852	38.666	14.344	2.2
3	8.954	1697.141	227.882	97.5

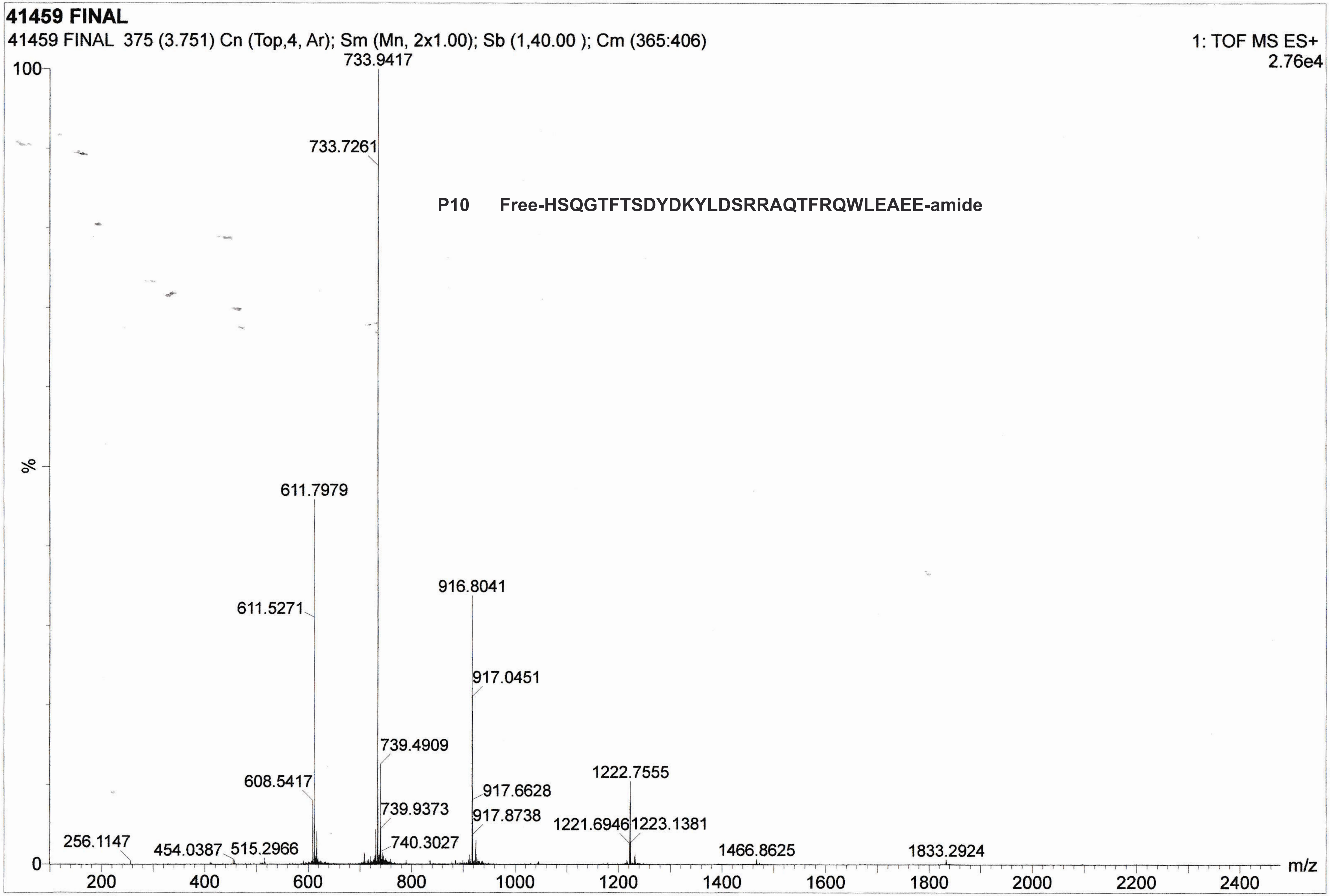

41459 FINAL
41459 FINAL 375 (3.751) Cn (Top,4, Ar); Sm (Mn, 2x1.00); Sb (1,40.00); Cm (365:406)
1: TOF MS ES+
2.76e4
P10 Free-HSQGTFTSDYDKYLDSRRAQTFRQWLEAEE-amide
733.9417
733.7261
611.7979
611.5271
916.8041
917.0451
739.4909
608.5417
1222.7555
917.6628
739.9373
917.8738
1221.6946
1223.1381
256.1147
454.0387
515.2966
740.3027
1466.8625
1833.2924
%
m/z
100
0
200
400
600
800
1000
1200
1400
1600
1800
2000
2200
2400

HPLC Analysis Report

Data File name:C:\CHEM32\1\DATA\CHECKOUT\13JULY20A 2020-07-13 08-12-49\ ->
Method name: C:\CHEM32\1\DATA\CHECKOUT\13JULY20A 2020-07-13 08-12-49\ ->
Injection date:: 13/07/2020
Sample Name:41460 B2 FINAL

P11 Free-HAEGTFTSDVASYLEGQAAKEFIPWLVKGR-amide

CAMBRIDGE RESEARCH BIOCHEMICALS

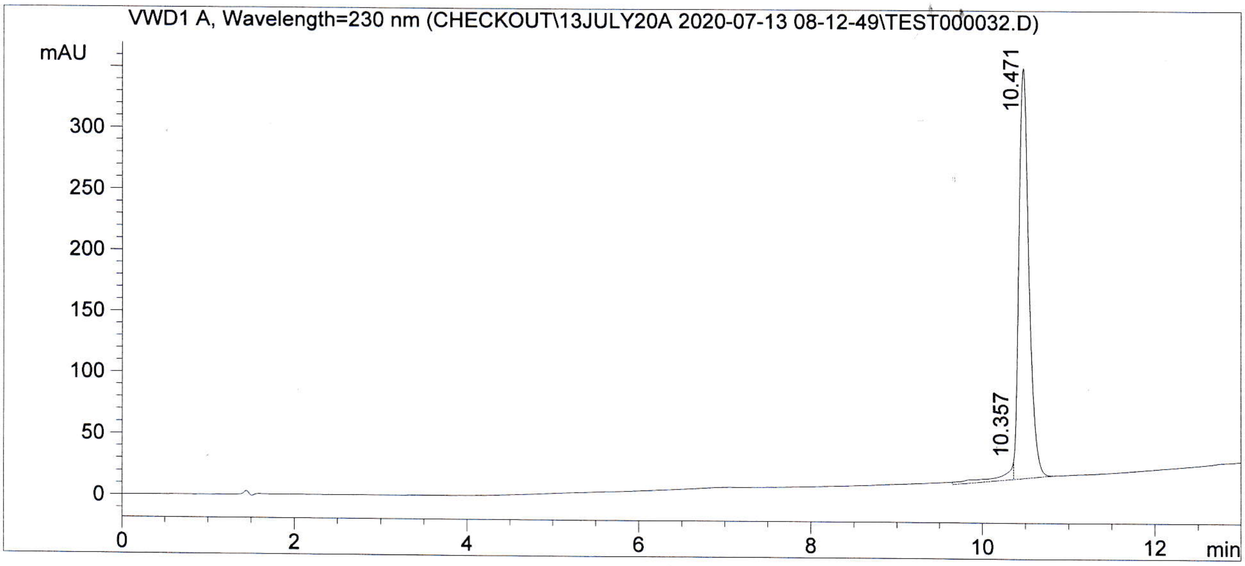

Column: ACE 3 C18-300 300A 150x2.1mm
Buffer A: 0.1 % TFA in H2O
Buffer B: 0.1 % TFA in MeCN
Flow rate: 0.35mL/min
Gradient: 2 to 70% B over 13 min

#	RT (Min)	Area	Height	Area %
1	10.357	138.048	12.982	4.8
2	10.471	2690.516	334.755	95.1

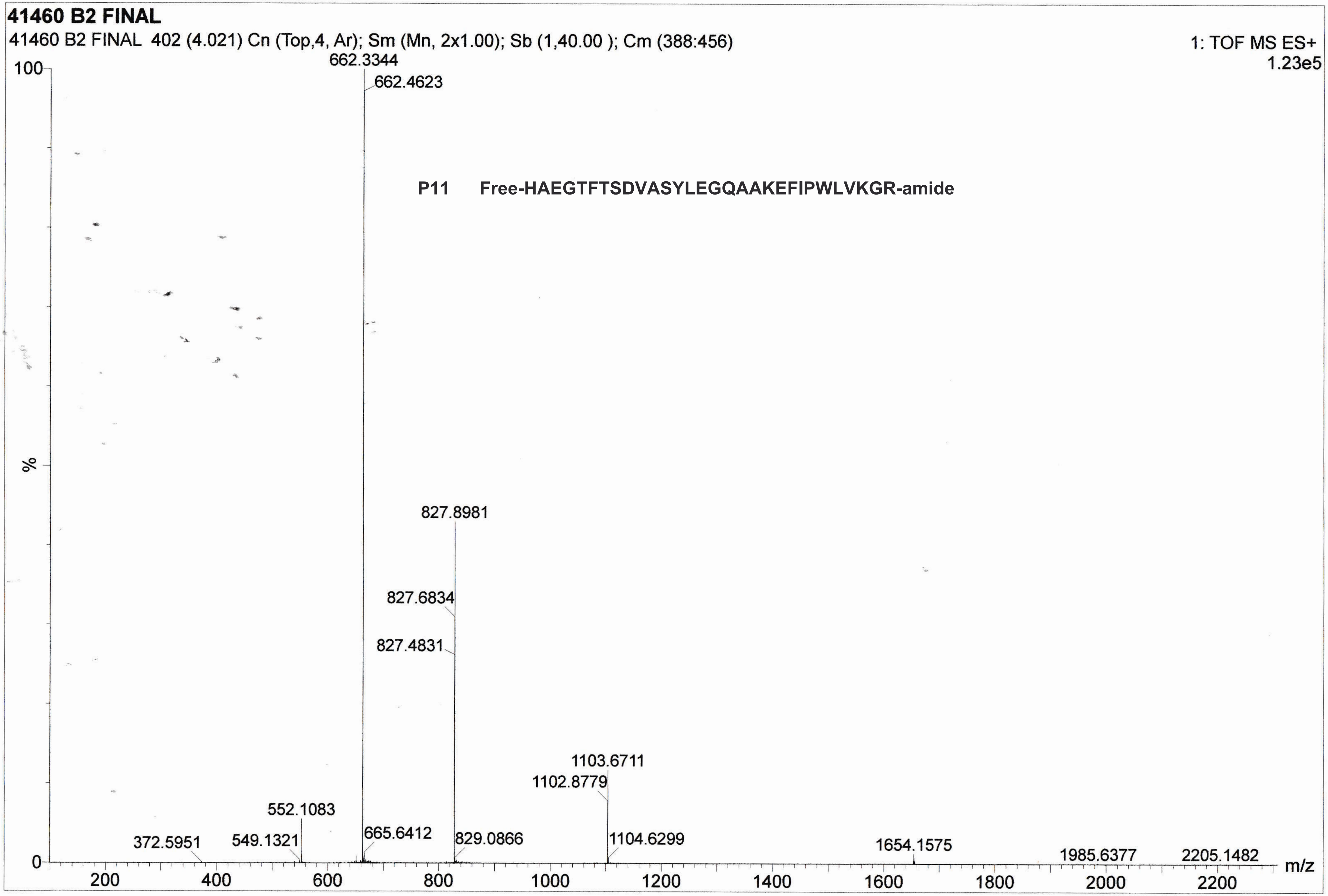

41460 B2 FINAL
41460 B2 FINAL 402 (4.021) Cn (Top,4, Ar); Sm (Mn, 2x1.00); Sb (1,40.00); Cm (388:456)
1: TOF MS ES+
1.23e5
P11 Free-HAEGTFTSDVASYLEGQAAKEFIPWLVKGR-amide
100
%
0
662.3344
662.4623
827.8981
827.6834
827.4831
1103.6711
1102.8779
552.1083
372.5951
549.1321
665.6412
829.0866
1104.6299
1654.1575
1985.6377
2205.1482
200
400
600
800
1000
1200
1400
1600
1800
2000
2200
m/z

HPLC Analysis Report

Data File name:C:\CHEM32\1\DATA\CHECKOUT\07JUL20A 2020-07-07 08-13-46\ ->
Method name: C:\CHEM32\1\DATA\CHECKOUT\07JUL20A 2020-07-07 08-13-46\ ->
Injection date:: 07/07/2020
Sample Name:41461 FINAL

P12 Free-YSQGTFTSDYSAYLEEEAVRFFINWLLAG-amide

CAMBRIDGE
RESEARCH BIOCHEMICALS

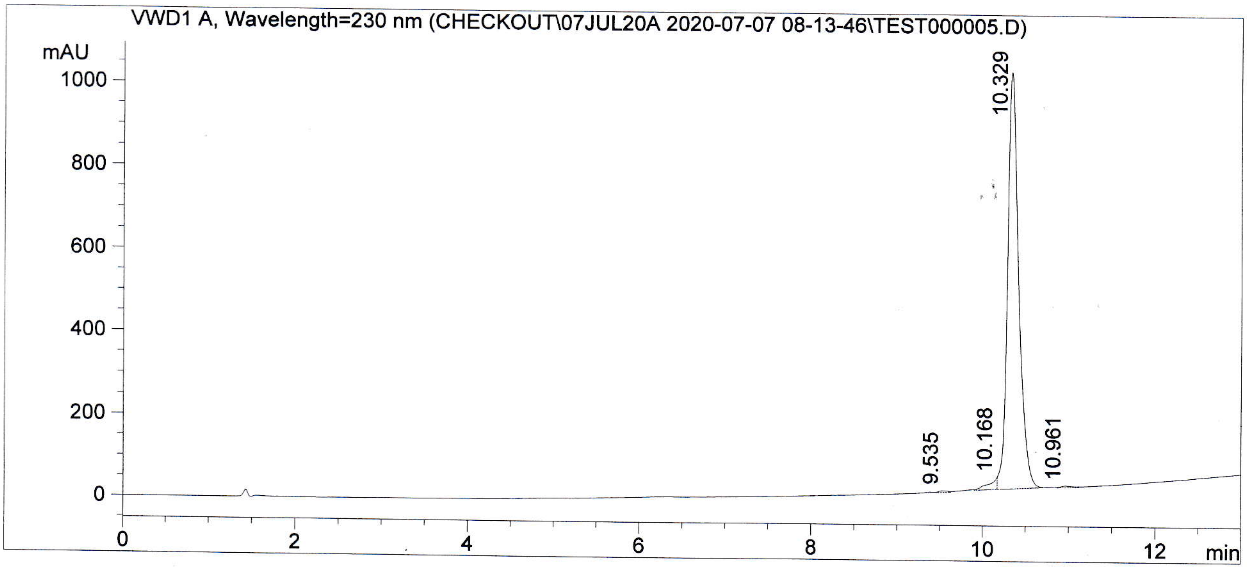

Column: ACE 3 C18-300 300A 150x2.1mm
Buffer A: 0.1 % TFA in H2O
Buffer B: 0.1 % TFA in MeCN
Flow rate: 0.35mL/min
Gradient: 2 to 70% B over 13 min

#	RT (Min)	Area	Height	Area %
1	9.535	32.309	4.344	0.3
2	10.168	191.598	28.221	2.0
3	10.329	9085.273	1003.981	97.1
4	10.961	47.235	4.319	0.5

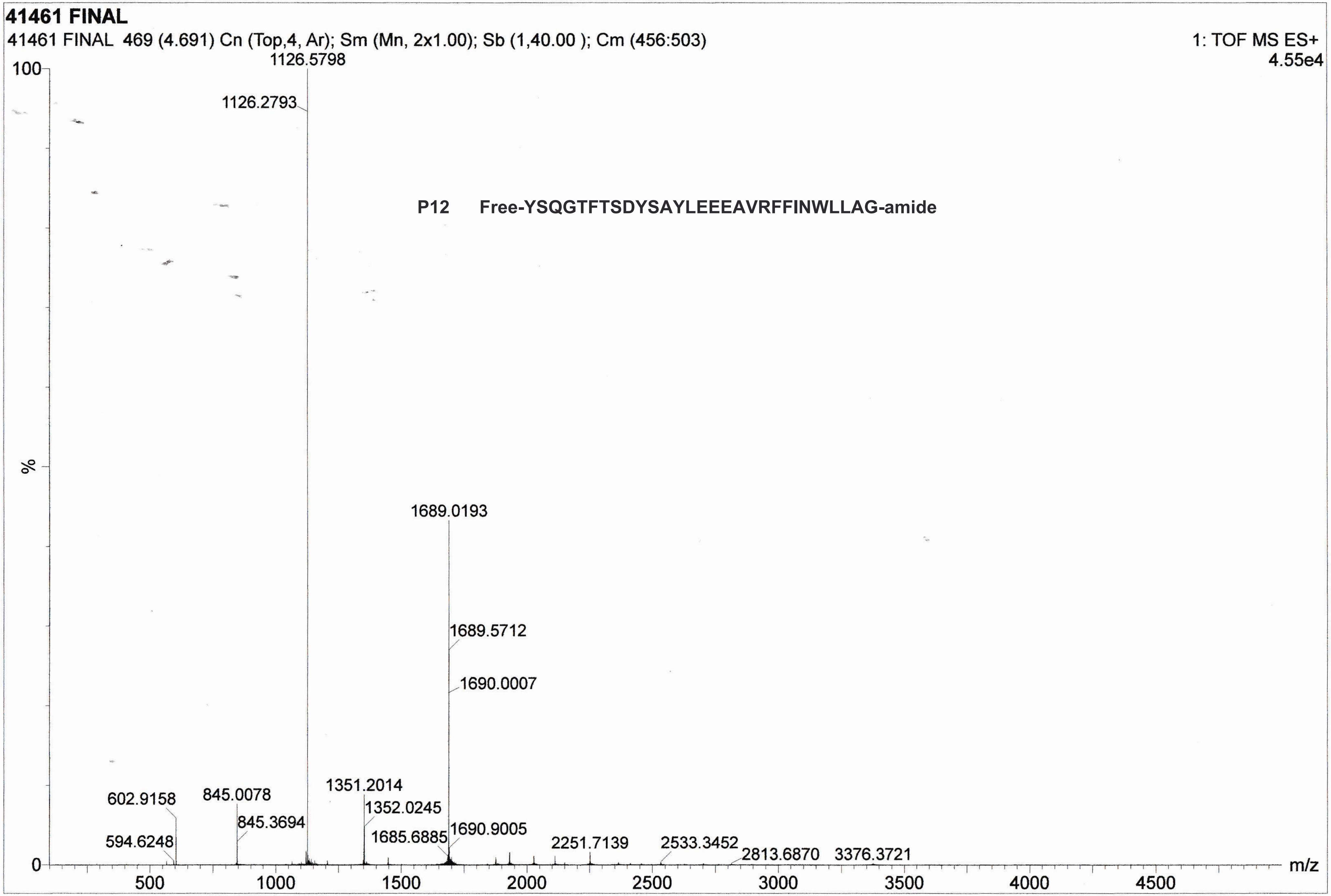
41461 FINAL
41461 FINAL 469 (4.691) Cn (Top,4, Ar); Sm (Mn, 2x1.00); Sb (1,40.00); Cm (456:503)
1: TOF MS ES+
4.55e4
P12 Free-YSQGTFTSDYSAYLEEEAVRFFINWLLAG-amide
1126.5798
1126.2793
1689.0193
1689.5712
1690.0007
602.9158
594.6248
845.0078
845.3694
1351.2014
1352.0245
1685.6885
1690.9005
2251.7139
2533.3452
2813.6870
3376.3721
100
%
0
500
1000
1500
2000
2500
3000
3500
4000
4500
m/z

HPLC Analysis Report

Data File name:C:\CHEM32\1\DATA\CHECKOUT\13JULY20A 2020-07-13 08-12-49\ ->
Method name: C:\CHEM32\1\DATA\CHECKOUT\13JULY20A 2020-07-13 08-12-49\ ->
Injection date:: 13/07/2020
Sample Name:41462 FINAL

P13 Free-YSQGTFTSDYSAYLEEEAVRDFITWLLAG-amide

CAMBRIDGE RESEARCH BIOCHEMICALS

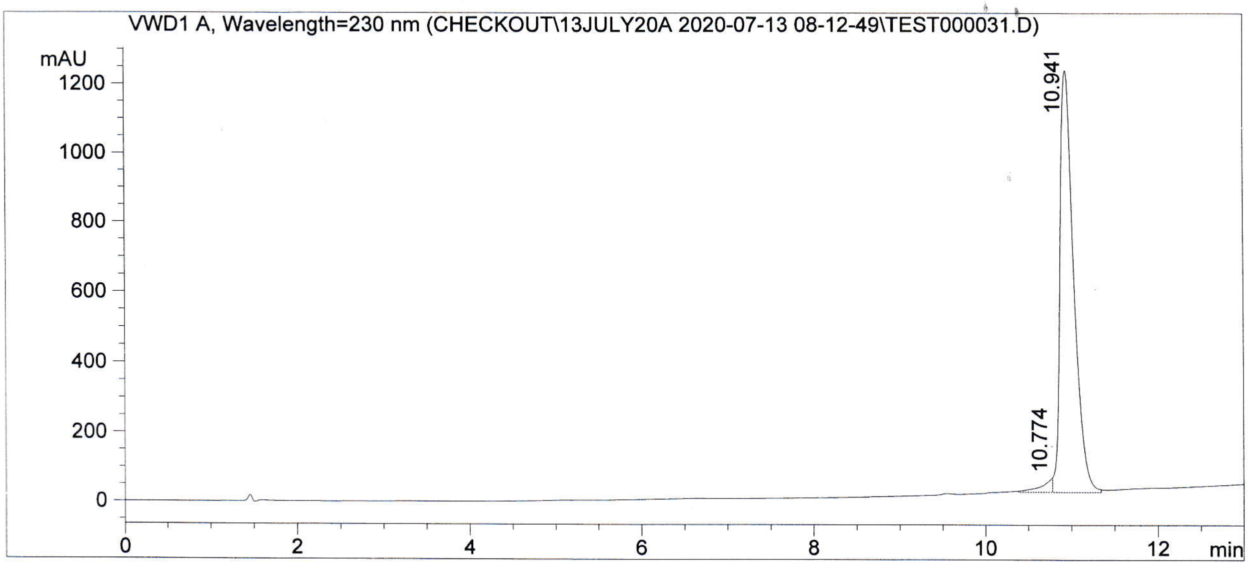

Column: ACE 3 C18-300 300A 150x2.1mm
Buffer A: 0.1 % TFA in H2O
Buffer B: 0.1 % TFA in MeCN
Flow rate: 0.35mL/min
Gradient: 2 to 70% B over 13 min

#	RT (Min)	Area	Height	Area %
1	10.774	362.474	40.464	2.6
2	10.941	13283.542	1211.260	97.3

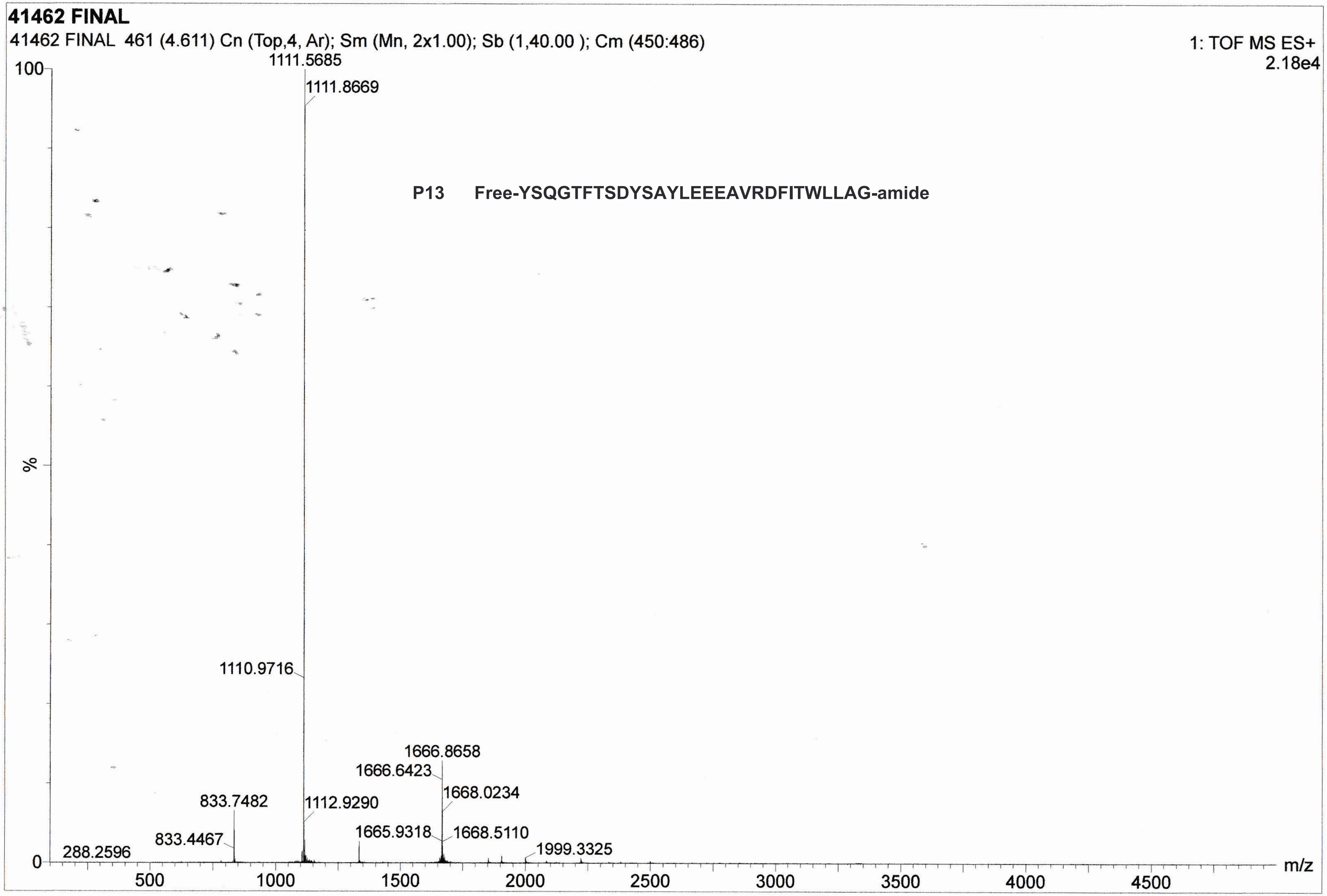
41462 FINAL
41462 FINAL 461 (4.611) Cn (Top,4, Ar); Sm (Mn, 2x1.00); Sb (1,40.00); Cm (450:486)
1: TOF MS ES+
2.18e4
P13 Free-YSQGTFTSDYSAYLEEEAVRDFITWLLAG-amide
1111.5685
1111.8669
1110.9716
1666.8658
1666.6423
1668.0234
833.7482
1112.9290
833.4467
1665.9318
1668.5110
288.2596
1999.3325
100
%
0
500
1000
1500
2000
2500
3000
3500
4000
4500
m/z

HPLC Analysis Report

Data File name:C:\CHEM32\1\DATA\15JULY20A 2020-07-15 07-16-24\TEST000025.D
Method name: C:\CHEM32\1\DATA\15JULY20A 2020-07-15 07-16-24\TEST000025.D\->
Injection date:: 15/07/2020
Sample Name:41463 FINAL

P14 Free-YSQGTFTSDYSAYLEEEAVRNFIWWLLAG-amide

CAMBRIDGE
RESEARCH BIOCHEMICALS

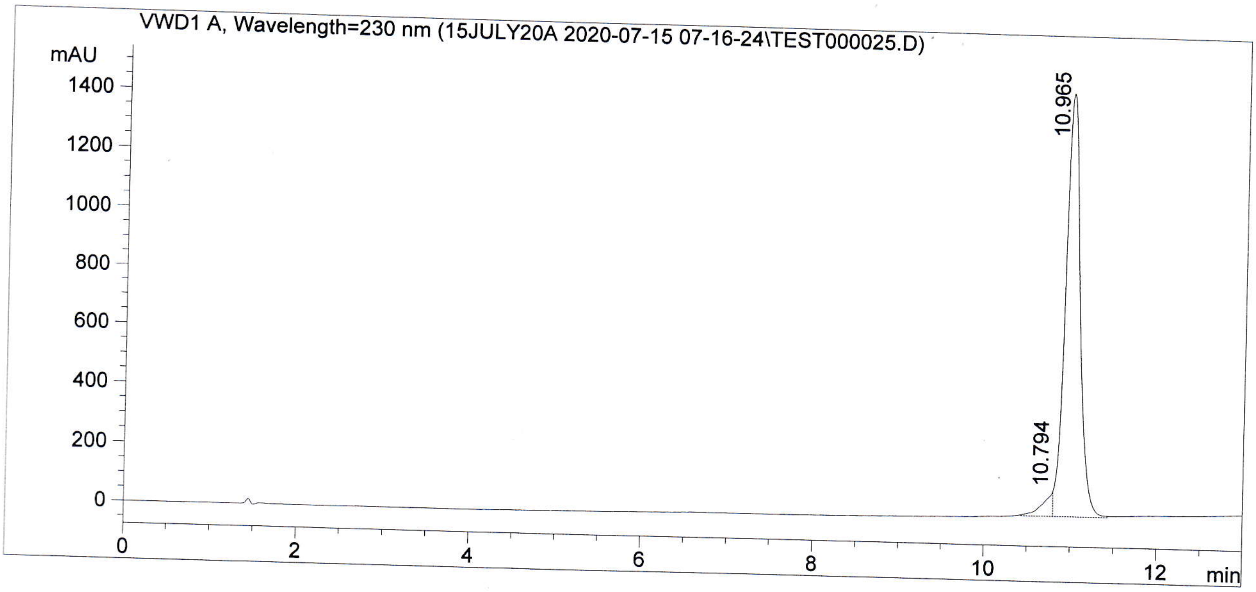

Column: ACE 3 C18-300 300A 150x2.1mm
Buffer A: 0.1 % TFA in H2O
Buffer B: 0.1 % TFA in MeCN
Flow rate: 0.35mL/min
Gradient: 2 to 70% B over 13 min

#	RT (Min)	Area	Height	Area %
1	10.794	686.785	82.292	3.9
2	10.965	16909.184	1441.107	96.0

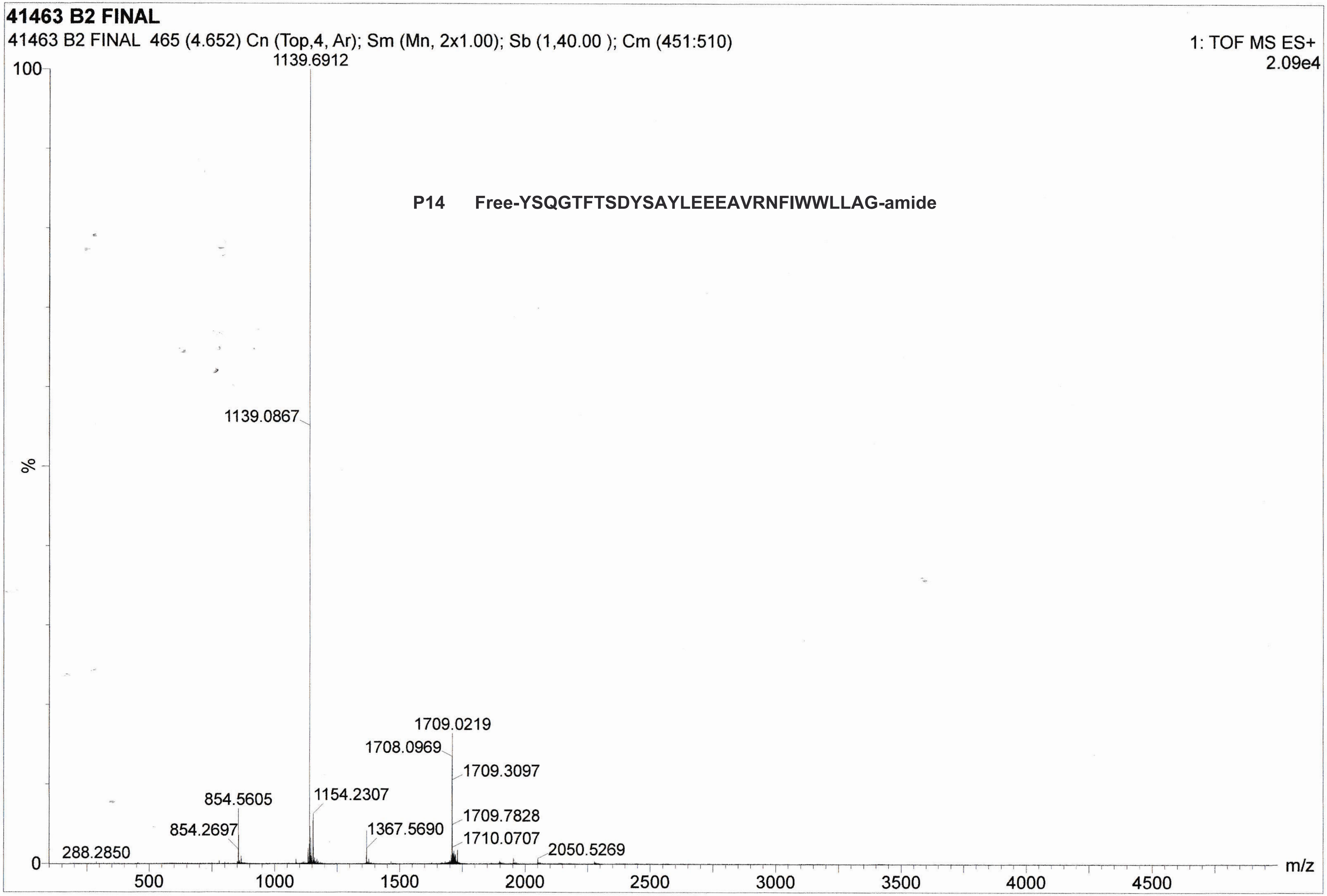

41463 B2 FINAL
41463 B2 FINAL 465 (4.652) Cn (Top,4, Ar); Sm (Mn, 2x1.00); Sb (1,40.00); Cm (451:510)
1: TOF MS ES+
2.09e4
100
%
0
1139.6912
1139.0867
1709.0219
1708.0969
1709.3097
854.5605
1154.2307
1709.7828
854.2697
1367.5690
1710.0707
288.2850
2050.5269
500
1000
1500
2000
2500
3000
3500
4000
4500
m/z
P14 Free-YSQGTFTSDYSAYLEEEAVRNFIWWLLAG-amide

HPLC Analysis Report

Data File name:C:\CHEM32\1\DATA\CHECKOUT\08JUL20A 2020-07-08 08-09-02\ ->
Method name: C:\CHEM32\1\DATA\CHECKOUT\08JUL20A 2020-07-08 08-09-02\ ->
Injection date:: 09/07/2020
Sample Name:41464 FINAL

P15 Free-YSQGTFTSDYSAYLEEEAVRNFIWWLLAG-amide

CAMBRIDGE RESEARCH BIOCHEMICALS

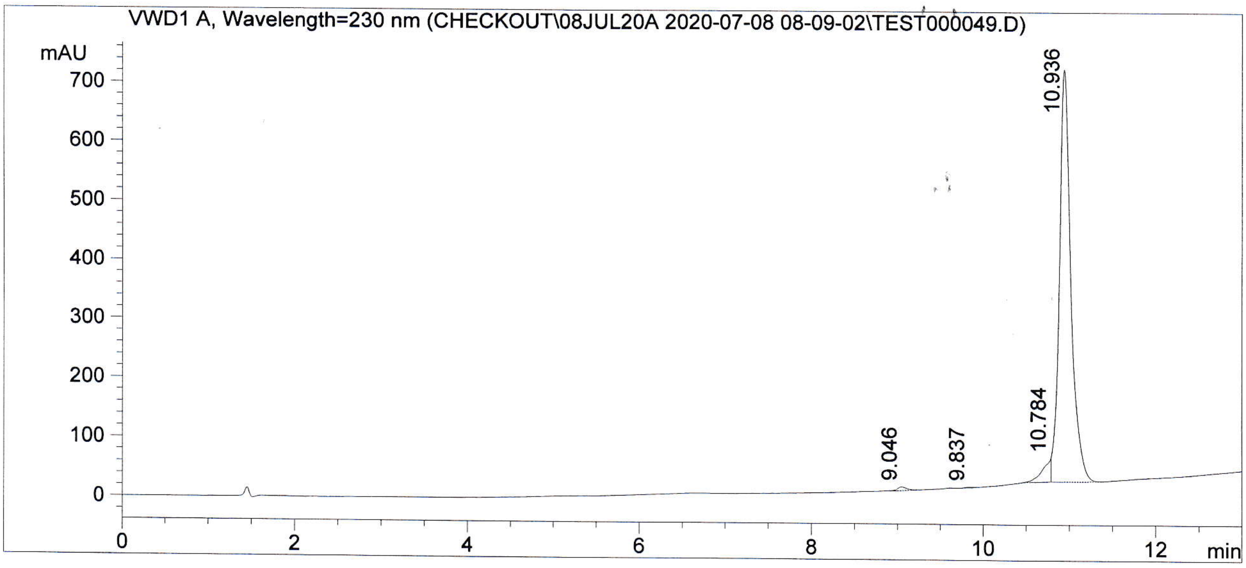

Column: ACE 3 C18-300 300A 150x2.1mm
Buffer A: 0.1 % TFA in H2O
Buffer B: 0.1 % TFA in MeCN
Flow rate: 0.35mL/min
Gradient: 2 to 70% B over 13 min

#	RT (Min)	Area	Height	Area %
1	9.046	52.526	6.479	0.7
2	9.837	10.983	1.475	0.1
3	10.784	270.906	39.064	4.0
4	10.936	6401.401	695.027	95.0

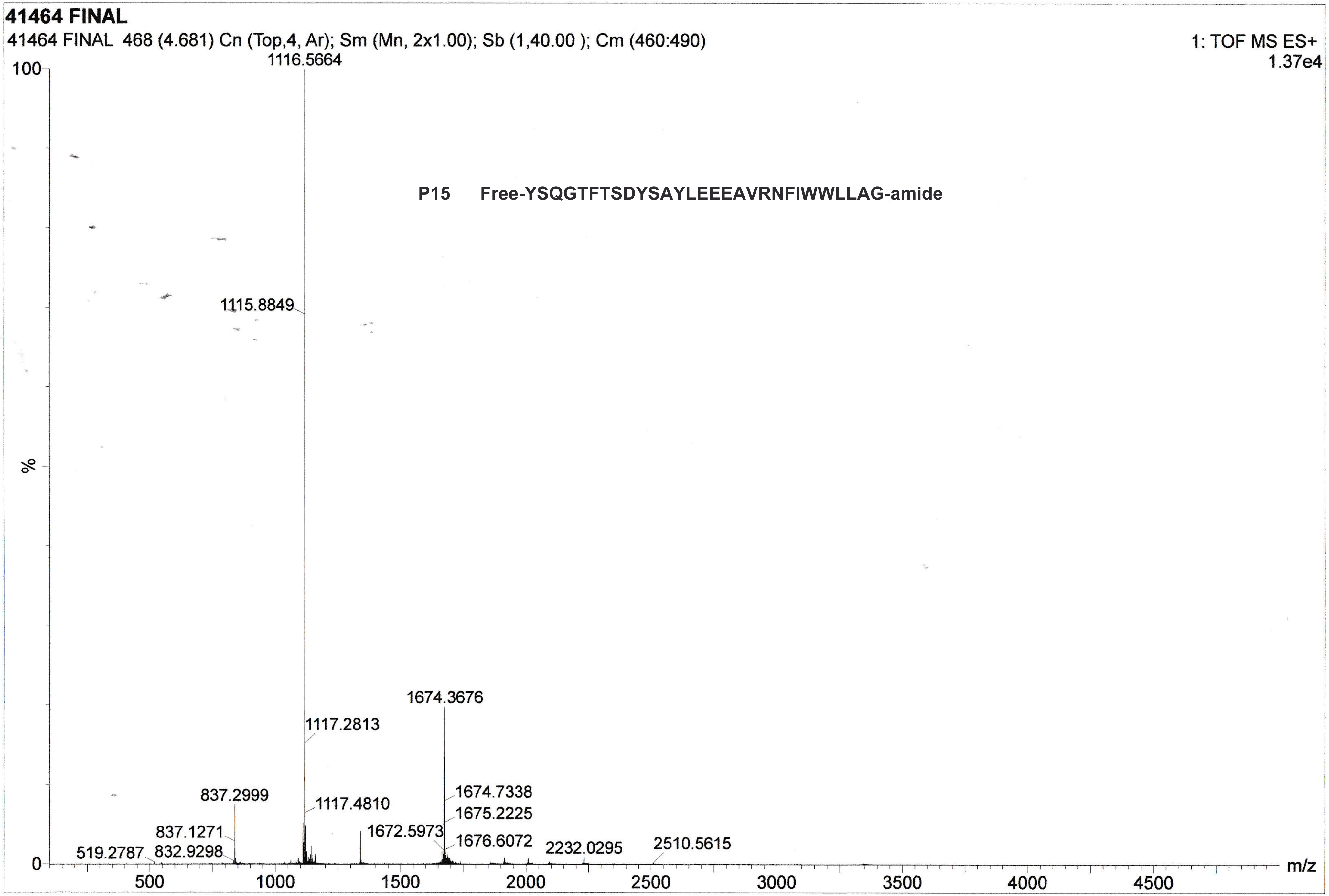

41464 FINAL
41464 FINAL 468 (4.681) Cn (Top,4, Ar); Sm (Mn, 2x1.00); Sb (1,40.00); Cm (460:490)
1: TOF MS ES+
1.37e4
P15 Free-YSQGTFTSDYSAYLEEEAVRNFIWWLLAG-amide
1116.5664
1115.8849
1674.3676
1117.2813
837.2999
1117.4810
1674.7338
1675.2225
837.1271
1672.5973
1676.6072
2232.0295
2510.5615
519.2787
832.9298
100
%
0
500
1000
1500
2000
2500
3000
3500
4000
4500
m/z
